# Extensive peripheral immunoglobulin repertoire analyses in people with multiple sclerosis reveal disease-specific signatures and distinct treatment effects of disease modifying drugs

**DOI:** 10.1186/s12974-026-03735-0

**Published:** 2026-04-14

**Authors:** Nicole Vasilenko, Christoph Ruschil, Josua Stadelmaier, Maria P. Tieck, Sonja Schembecker, Gregory P. Owens, Jeffrey L. Bennett, Achim Berthele, Ulf Ziemann, Sven Poli, Nicolas Snaidero, Sven Nahnsen, Mohamed A. Jarboui, Leon Bichmann, Gisela Gabernet, Markus C. Kowarik

**Affiliations:** 1 Hertie-Institute for Clinical Brain Research, Eberhard-Karls University of Tübingen, Tübingen, Germany; 2 Department of Neurology & Stroke, Eberhard-Karls University of Tübingen, Tübingen, Germany; 3 Department for Computer Science, Biomedical Data Science, Eberhard-Karls University of Tübingen, Tübingen, Germany; 4 Quantitative Biology Center (QBiC), Eberhard-Karls University of Tübingen, Tübingen, Germany; 5 Departments of Neurology, University of Colorado School of Medicine, Anschutz Medical Campus, Aurora, USA; 6 Departments of Neurology and Ophthalmology, Programs in Neuroscience and Immunology, University of Colorado School of Medicine, Anschutz Medical Campus, Aurora, USA; 7 Department of Neurology, School of Medicine and Health, Technical University of Munich, Munich, Germany; 8 Core Facility for Medical Bioanalytics (CFMB), Eberhard Karls University of Tübingen, Tübingen, Germany; 9 Center for Systems and Engineering Immunology, Yale University, New Haven, USA; 10 Department of Pathology, Yale School of Medicine, New Haven, USA

**Keywords:** Multiple sclerosis (MS), B cells, Immunoglobulin repertoire, Disease modifying drugs (DMDs), Immunoglobulin proteome, Epstein-Barr virus (EBV)

## Abstract

**Background:**

B cells are crucial players in the pathogenesis of multiple sclerosis (MS), however, limited information is available on peripheral immunoglobulin (Ig) repertoires of people with MS (pwMS) in comparison to healthy individuals and during different treatments.

**Methods:**

Next generation sequencing of Ig heavy chains (VH) originating from bulk-sorted B cell populations was performed in 33 pwMS and ten healthy controls. All 33 pwMS were examined longitudinally at baseline and six months after treatment with ozanimod, fingolimod, dimethyl fumarate, teriflunomide, cladribine or natalizumab. Ig peptides were obtained longitudinally in a subset of treated pwMS by Ig mass spectrometry, overlapped with VH transcriptomes through nf-core bioinformatics workflow analysis and additionally Ig serum level and anti-Epstein-Barr virus IgG level (EBNA1) were measured.

**Results:**

VH repertoires of treatment-naïve pwMS showed a significantly decreased diversity in the double negative B cells and different usage of IGHV genes when compared with healthy controls. Quantitative changes in B cell subsets during treatment were accompanied by qualitative changes in Ig repertoires with a significantly decreased diversity in the naive, memory B cells and plasmablasts during ozanimod treatment. A similar trend was noticeable for all other treatments except natalizumab. No qualitative change in Ig peptides overlapping with Ig transcriptome repertoires was observed.

**Conclusions:**

This study provides first evidence for an altered peripheral Ig repertoire in pwMS. In addition, various treatments seem to shift the composition of B cells towards an increased fraction and activation of the naive B cell pool and a reduced fraction of memory B cells with reduced clonal diversity.

**Graphical Abstract:**

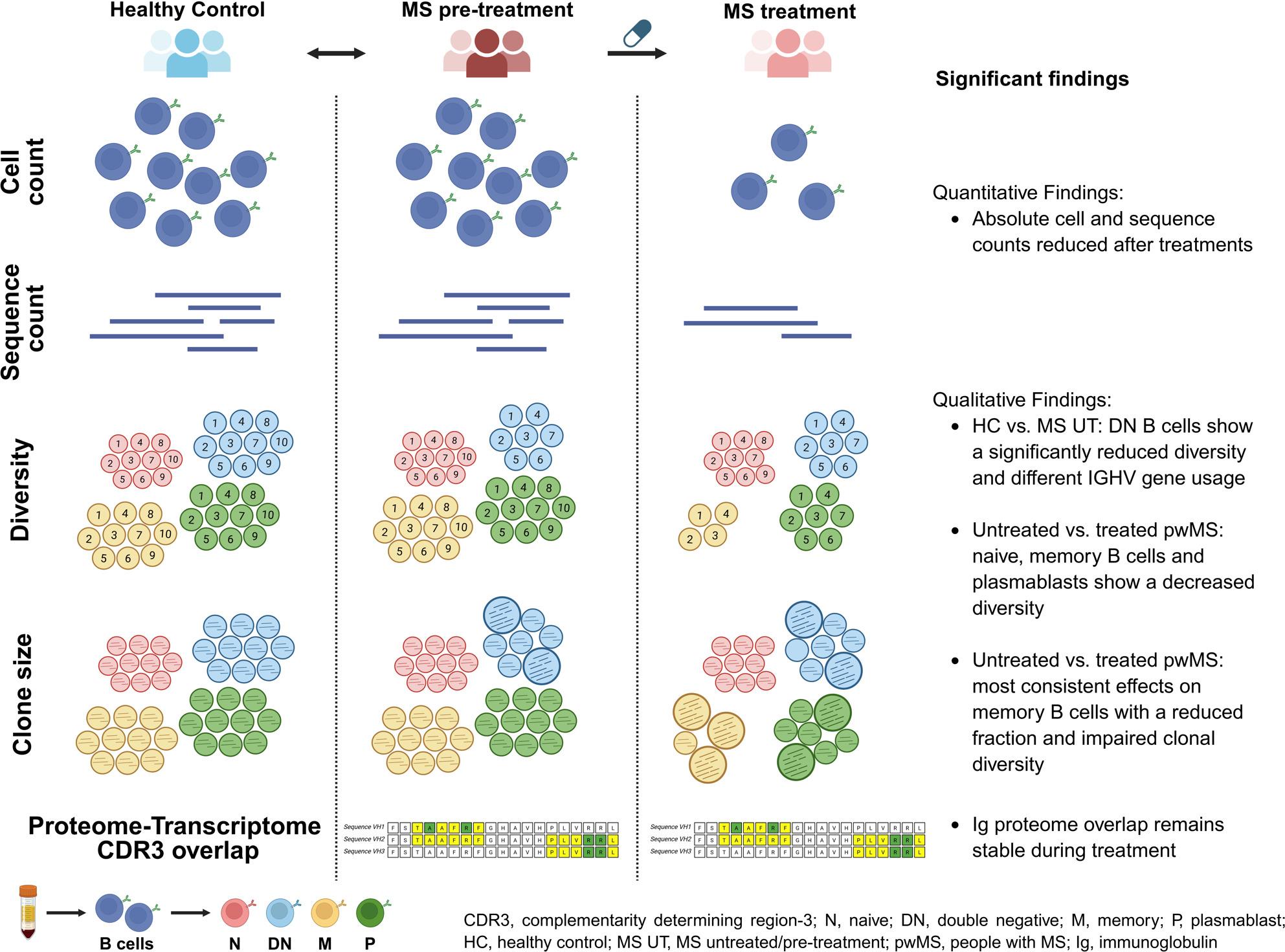

**Supplementary Information:**

The online version contains supplementary material available at 10.1186/s12974-026-03735-0.

## Background

Multiple sclerosis (MS) is an autoimmune disorder that results in acute and chronic inflammation of the central nervous system (CNS) involving both the innate and adaptive immune system [1]. B cells have been identified as pivotal contributors of the adaptive immune response to the pathogenesis of MS, as evidenced by the substantial efficacy of anti-CD20 B cell-depleting substances such as ocrelizumab, ofatumumab or ublituximab [2–5]. Regarding the pathophysiology of CNS inflammation, B cells have been identified in lesions and follicular structures within the meninges, and have been shown to produce cerebrospinal fluid (CSF) oligoclonal bands [6], which are implemented in the diagnostic criteria for MS [7]. Despite the low transfer of antibodies across the blood-brain barrier, anti-CD20 therapy has been shown to reduce CSF B cell numbers [8, 9]. Current MS dose regimens that include different antibody concentrations and application forms for anti-CD20 treatments all result in near-complete depletion of circulating B cells and good clinical efficacy [10]. However, the peripheral B-cell depletion may be associated with varying degrees of B-cell depletion in lymphoid tissues, the central nervous system, and the leptomeninges [10]. These findings, when considered in conjunction with the absence of strong beneficial effects observed with intrathecal administration of anti-CD20 treatments [11–13], indicate that a significant effect of anti-CD20 treatments may be associated with alterations in peripheral B cell populations.

Little information is available on the peripheral blood immunoglobulin (Ig) repertoire in untreated people with MS (pwMS). Quantitative, flow-cytometric analyses show only modest alterations in major peripheral B cell populations, including naive (N), memory B cells (M) and plasmablasts (P) [14, 15]. The prevalence of transitional/regulatory B cells was often lower in MS [16], while double negative (DN) B cells showed partial up-regulation [17, 18]. Early studies on qualitative Ig repertoire changes only included low patient numbers and primarily concentrated on the trafficking patterns of B cells between the periphery and the CSF showing a consistent bidirectional exchange of B cells across the blood-brain barrier [15, 19–21], However, the extant literature offers only limited analyses of Ig repertoires in pwMS when compared to healthy controls (HC) [22, 23].

Besides the targeted depletion of peripheral B cells through anti-CD20 treatment, several disease modifying drugs (DMDs) have been shown to exert differential effects on peripheral blood B cell subsets [14, 15]. Treatments such as fingolimod (FTY), dimethyl fumarate (DMF), teriflunomide (TER) and cladribine (CLAD) have been shown to reduce the overall number of circulating B cells while interferon (IFN) beta treatments and natalizumab (NAT) increased peripheral B cell numbers [14, 15]. Concerning the percentage distribution of the various B cell subsets (naive, double negative, memory B cells and plasmablasts) within the B cell fraction during different treatments, a consistent reduction of the memory B cell subset was observed for FTY, TER, IFN, CLAD and DMF treatments [14, 15]. A notable exception is observed with NAT treatment, which results in an increased peripheral memory B cell fraction due to the release of marginal zone B cells from the spleen via alpha-4 integrin blockade [21]. However, when interpreting these findings, it must be considered that in general B cells are inhibited by integrin blockage in their ability to cross the blood-brain barrier, despite its increased permeability in MS [21]. While these studies mostly relied on flow cytometry to quantify the different immune cell subsets, immunoglobulin repertoire analysis by next generation sequencing (NGS) sequencing approaches provide a unique opportunity to gain qualitative insights into peripheral B cells [24].

The available Ig repertoire data on treatments mostly studied the effects of different treatments within the CSF or B cell trafficking patterns between the periphery and the CNS [21, 25]. Although these studies provided informative data regarding the persistence of CSF clones during treatment, information is sparse detailing Ig repertoire changes in the peripheral blood during MS treatments. Our group recently reported detailed peripheral Ig repertoire data, demonstrating profound changes in the clonal composition of memory B cells following cladribine treatment [26]. These findings are consistent with another study, which also demonstrated significant alterations in the transcriptome profiles, including Ig transcripts, particularly within the memory B cell subset after treatment with alemtuzumab [27]. Despite the ambiguity surrounding the precise function of B cells and the ongoing debate on the production of cross-reactive antibodies via molecular mimicry or their potential role in antigen presentation, the redundant effects of different treatments on peripheral memory B cells are of significant interest in the context of Epstein-Barr virus (EBV) persistence within this B cell subset [28].

Besides the analysis of quantitative effects on B cell subsets, this study focused on assessing qualitative effects on Ig (heavy chain, VH) repertoires at the transcriptome level. Furthermore, an assessment of serum Ig proteins was conducted, by matching the identified peptides with the Ig transcriptomes. This approach was taken to obtain a comprehensive depiction of peripheral blood Ig repertoire alterations at the cellular and protein levels. The study encompassed a total of 33 pwMS who were evaluated at two distinct time points: at the initial baseline (BL) and subsequently six months after the initiation of treatment (follow-up six months, FUP6) regimens comprising ozanimod (OZA), fingolimod, dimethyl fumarate, teriflunomide and natalizumab, and for further comparisons cladribine. Additionally, ten individuals serving as controls were included in the study. By this approach we aimed to (I) further analyse MS specific repertoire changes when compared to healthy controls, (II) characterize treatment specific effects on B cell repertoires including treatment with OZA, FTY, DMF, TER, NAT and (III) examine how the observed transcriptome changes during treatments translate into effects on circulating Ig proteome.

## Methods

### Study approvals, ethics, registrations and patients

Patients with relapsing remitting multiple sclerosis (Table 1, Suppl. Table 1) were recruited at the neuroimmunological outpatients clinics of the Eberhard Karls University of Tübingen (TÜ; OZA1-9, DMF6-9, TER6-7, CLAD1-8, HC1-4), at the Technical University of Munich (TUM; TER1-3, HC5-10) and at the University of Colorado, School of Medicine (US; FTY1-4, NAT1-4) (Suppl. Table 2). Study inclusion criteria were as follows: (I) diagnosis of clinically definite MS according to revised McDonald Criteria (McDonald 2010 [29] or 2017 [7]); (II) age between 18 and 55 years; (III) the ability to give informed consent; (IV) expanded disability status scale (EDSS) ≤ 5.0; (V) treatment-naïve or pretreated patients with an interval to premedication > 5 half-life periods for previous treatment and normalization of differential blood values. The exclusion criteria included (I) prior immunomodulatory or immunosuppressant therapy with methotrexate, cyclophosphamide, mitoxantrone, rituximab, ocrelizumab, daclizumab, mycophenolate mofetil, laquinimod, S1P receptor modulators or total body irradiation; (II) vaccination < 4 weeks before sample collection; (III) diagnostic uncertainty; (IV) concomitant auto-immune diseases requiring immunomodulation and (V) chronic infectious diseases and (VI) no clinical relapse and / or steroid therapy within the last 30 days. Further criteria are available elsewhere [21, 26]. All medications were pre-determined by the patient’s treating physician before study inclusion.

### Peripheral blood collection, cell staining and B cell sorting

Peripheral blood (15 mL in EDTA tubes, 7.5 mL for serum) was collected from all patients and peripheral blood mononuclear cells (PBMCs, using a Ficoll gradient protocol, stored in liquid nitrogen) and serum were processed within two hours. Following antibodies were used for flow cytometric analyses: CD38 FITC (BD), IgD PE (Biozol), CD19 ECD (Beckman Coulter), CD3 PeCy7 (Beckman Coulter), CD45 VM450 (BD), CD27 APC (BD), CD20 APC Cy7 (BD). Gating strategy was applied as described previously [18]. B cells were subdivided into following four B cell subsets: naive B cells (CD19^+^ CD20^+^ CD27^−^ CD38^−/low^ IgD^+^), memory B cells (CD19^+^ CD20^+^ CD27^+^ CD38^low^), double negative B cells (CD19^+^ CD20^−/low^ CD27^−^ IgD^−^) and plasmablasts (CD19^+^ CD20^−/low^ CD27^+^ CD38^high^ IgD^−^ ).

### Library preparation, Ig sequencing and data analysis

NGS of Ig variable heavy chain (VH) transcripts was performed as described previously [21]. In brief, cryopreserved PBMCs were thawed at 37°C, washed in phosphate buffered saline (PBS), incubated with the B cell antibody panel described above (light protected, 30 min, 4°C), washed twice in PBS and finally resuspended in FACS buffer (PBS, 2% FCS, 2 mM EDTA, 0.01% NaN_3_). B cells were bulk sorted on a FACSAriaIII (BD bioscience) into the four target populations N, DN, M and P, thereby collected in FACS buffer. RNA was extracted immediately thereafter from each population (Qiagen RNeasy Plus Micro Kit or Macherey Nagel NucleoSpin RNA Plus XS, manufacturer’s instructions) and frozen at -80°C till cDNA synthesis. For cDNA synthesis the SMART-Seq^®^ v4 Ultra^®^ Low Input RNA Kit and the SeqAmp™ DNA Polymerase (Takara Bio) for Illumina sequencing were used (manufacturer’s instructions). The cDNA synthesis protocol was modified by additional external constant region primers for IgA, IgG, IgM and IgD. A subsequent nested polymerase chain reaction (PCR, expand high fidelity PCR system, Roche) was conducted to amplify the variable heavy chain domain. A pool of VH-family-specific (VH1-VH5) and isotype-specific (IgD, IgM, IgG, and IgA) primers was used in separate PCR reactions for each VH-family and isotype to avoid cross-priming / primer competition. The VH-family-specific primers contained an index-tag (six nucleotides) for labeling the cell subset, and a unique molecular identifier (UMIs) consisting of 8 or 12 random nucleotides for subsequent sequencing, controlling for sequence duplicates that originate from PCR amplification. VH-specific cDNA libraries were pooled (8 or 12 libraries per pool) and sequenced on the MiSeq sequencing system (Illumina). The raw sequencing data was subsequently processed using the nf-core/-airrflow pipeline (version 4.2.0, open source script available at http://github.com/nf-core/airrflow), which uses the Immcantation Framework toolset [30]. For sequence read quality control and sequence assembly FastQC and the pRESTO [31] toolset were used, respectively. Reads were filtered according to base quality (quality score threshold of 20). Consensus sequences were built by pairing forward and reverse reads with the same UMI based on a maximum mismatch error rate of 0.1 per read group. Unique V(D)J sequences were confirmed as valid when being represented by at least five sequences with the same UMI. Sequence copies were defined as identical sequences with different UMI barcodes. VH variable-diversity-joining [V(D)J] germline segments were assigned by blasting the processed sequences to the IMGT database with IgBLAST [32] and Change-O [33]. SCOPer [34, 35] was used to assign functional V(D)J sequences into clones based on (I) identical nucleotide complementarity determining region-3 (CDR3) sequence length, (II) same IGHV and IGHJ gene, and (III) single-linkage clustering of the junction nucleotide sequence. The junction sequence consists of the CDR3 sequence and the nucleotides encoding the two flanking amino acids (AA) in the 5’ and 3’ end. As a distance metric for single-linkage clustering, the length-normalized hamming distance of the nucleotide junction sequences is used. The threshold distance for defining clonal relationships was set to 0.1. The frequency of somatic hypermutations was computed with SHazaM [33]. Departing from the nf-core/airrflow output, further downstream analyses have been performed using Python and R scripts (available at https://github.com/qbic-projects/MS-treatment-study*).* These analyses include clonal abundance and diversity calculations using Alakazam [33] on a bootstrap sample of *n* = 126 (required *n* ≥ 100) unique sequences with 200 repetitions. Samples not passing the threshold of *n* = 100 sequences were excluded from the data set (Suppl. Table 3, outliers highlighted in light grey). Clonal overlaps between time points were estimated on the same bootstrap sampling procedure with a custom R script. Subsampling was implemented here to facilitate the comparison of the samples of different sizes.

### Ig purification from serum, mass spectrometry of Ig peptides and proteomic data analysis

Serum samples from the OZA, DMF and TER patient cohorts were analyzed on Ig peptides. Ig enrichment was conducted in Pierce™ spin columns by using CaptureSelect™ LambdaXP and KappaXP affinity beads (Thermo Fisher Scientific, manufacturer’s instructions). Ig’s were collected in glycine buffer (0.1 M; pH 2–3) and stored at -80 °C. The protein concentration was determined via Bradford assay. For protein collection, the methanol / chloroform precipitation method was applied. Similar amount of total proteins was used per sample for the in-solution digest with trypsin. Resulting Ig peptides were analyzed with liquid chromatography tandem mass spectrometry (LC-MS/MS) on a linear trap quadrupole (LTQ) Orbitrap Velos mass spectrometer (Thermo Fisher Scientific). Detailed methodology is described elsewhere [26, 36]. Samples were measured in data-dependent acquisition (DDA) mode in case of DMF and TER, and the OZA data set was measured in optimized data-independent acquisition (DIA) mode at a later stage due to method availability. We performed Ig extraction prior to mass spectrometry and consistently applied the same method for the longitudinal analysis of each patient subgroup to avoid bias from using these two slightly different methods. The raw mass spectra data was analyzed using the workflow nf-core/quantms [37] (version 1.0) selecting Comet [38] and Percolator [39] for DDA- and DIA-NN [40] for DIA-based library free peptide database search. A stringent peptide-level FDR cut-off of 0.1 for DDA and 0.01 for DIA was set. Patient-specific Ig sequence databases for the peptide search were generated from the translated AA sequence of the corresponding Ig transcriptomic data, thereby combining the BL and FUP6 sequences to one database for a more sensitive peptide identification. Data was analyzed as described previously [26]. Downstream analyses were performed with in-house R scripts that were adapted individually for each data set with considering the DIA and DDA modes (https://github.com/qbic-projects/MS-treatment-study). Ig sequences were considered a match if covered by unique peptides with ≥ 30% identity to the somatically mutated CDR3 cDNA sequence of the VH sequence. For the transcriptome-proteome alignments, transcriptome BCR sequences were considered across the repertoires before and after treatment to match with the proteome samples.

### Serum IgG antibody titer measurements against EBV and molecular mimicry peptides

Serum samples from the OZA patient cohort were additionally measured in an in-house multiplex-assay and analyzed on IgG antibodies targeting EBNA1 (full protein and p72) and specific EBNA1 peptides (AA386-405, AA393-412, AA425-444) as well as against potential molecular mimicry antigens including GlialCAM (AA370-389), CRYAB (AA2-21) and ANO2 (AA134-153) peptides. A detailed description on conduction and analysis was described previously [28].

### Ig subclass measurements

In the OZA cohort (OZA1-OZA9), levels of serum IgA, IgG, IgM and IgG subtypes 1–4 were measured via Atellica^®^ CH Ig test and NEPH 630 system according to manufacturer’s instructions, respectively, at all time points.

### Sex as biological variable

Various testings were applied in order to analyse on sex-specific differences. First, sub-analyses were performed by combining all treatment-naïve pwMS from the different treatment cohorts and hereby comparing male and female subjects (Suppl. Figure 1). No significant differences between male and female pwMS were found. Additionally, the effect of sex as a co-factor on group comparisons was analyzed using multiple linear regression. For none of the analyses a significant influence of the co-factor sex was observed (data not shown).

### Statistics

Statistical analyses and corresponding data visualization were performed with either GraphPad Prism (Version 10.2.2), IBM SPSS Statistics (Version 30.0.0) or R software (Version 4.3.2). In addition, the graphical abstract was created using BioRender. When performing longitudinal analyses on treatment response the Wilcoxon Rank-Sum paired test was performed. For cross-sectional comparisons the Mann-Whitney test was used for comparing two independent groups and the Kruskal-Wallis test with Dunn’s correction was performed in case of multiple group comparisons. For clonal overlap analyses the Kruskal-Wallis test with Benjamini-Hochberg correction was applied. Given that the various data sets were assessed in different batches and Ig repertoire sequencing is prone to method-immanent bias, we performed further sub-analyses and various statistical methods to validate for robustness of the presented cross-sectional data and appropriate biological data interpretation. A detailed description of these sub-analyses and related supporting data can be found in supplementary methods (*Variance analyses and outlier detection*).

## Results

### Study cohort and clinical data

In this study, 33 patients with relapsing-remitting MS were enrolled according to our inclusion / exclusion criteria and analyzed longitudinally at baseline and after six months of DMD treatment. For cross-sectional analyses, we also included ten healthy controls who did not receive any vaccination four weeks prior to analysis and did not show any signs of recent infection. The MS study cohort was further subdivided according to the following treatment groups: ozanimod (OZA, *n* = 9), fingolimod (FTY, *n* = 3), dimethyl fumarate (DMF, *n* = 4), teriflunomide (TER, *n* = 5), natalizumab (NAT, *n* = 4) and cladribine (CLAD, *n* = 8). A previous analysis of MS patient groups treated with FTY, NAT, and CLAD has been published [21, 26], however, further development of the analysis pipeline has since occurred [30], prompting the reanalysis of these data sets using the updated bioinformatics pipeline to facilitate direct comparisons across all treatments. The clinical data is summarized in Table 1 for the main study cohort and in Suppl. Table 1 for all comparison groups. With the exception of patients OZA5, FTY3, FTY4, NAT3, and NAT4, all other patients had not received prior MS specific treatment (treatment-naïve). Relapses and concomitant methylprednisolone treatment were at least four weeks apart from the time of Ig repertoire analysis. During the initial six-month treatment period, 15 patients exhibited a stable disease course, four patients demonstrated MRI activity with or without worsening of EDSS, three patients experienced an EDSS worsening, and three patients demonstrated an improvement in their EDSS. An additional clinical follow-up after 12 months of treatment (FUP12, no Ig repertoire analyses performed except for CLAD, Suppl. Table 4) was assessed; nine patients exhibited a stable disease course, two patients demonstrated an EDSS worsening without MRI or relapse activity, and one patient discontinued therapy due to relapse and MRI activity. No cases of serious infections or serious adverse effects were reported.

**Table 1:** Patient characteristics of main study cohort

ID	Sex	Age BL	First reported relapse (y)	Date of diagnosis (m – y)	CSF status (OCB) at diagnosis	Previous therapies	Date study inclusion (d – m – y)	Disease duration (m)	EDSS BL	EDSS FUP6	Disease activity during 6 m of treatment
OZA1	f	37	2021	Apr – 2021	positive	-	08 – Jul – 2021	3	1.5	1.5	stable
OZA2	m	30	2021	Jun – 2021	positive	-	08 – Jul – 2021	1	1.0	1.5	EDSS↑, MRI activity
OZA3	m	26	2021	May – 2021	negative	-	14 – Jul – 2021	3	1.0	1.0	stable
OZA4	f	20	2021	Jul – 2021	positive	-	24 – Aug – 2021	2	0.0	0.0	stable
OZA5	m	41	2010	n.a. – 2010	n.a.	INF	24 –Jun – 2022	138	1.5	1.5	stable
OZA6	f	43	2022	Mar – 2022	positive	-	29 –Jun – 2022	4	1.5	1.5	stable
OZA7	f	19	2022	Jul – 2022	positive	-	12 – Jul – 2022	< 1	2.0	1.0	EDSS ↓
OZA8	f	24	2022	Jul – 2022	negative	-	12 – Aug – 2022	1	0.0	0.0	stable
OZA9	m	27	2023	Feb – 2023	positive	-	03 – Feb – 2023	2	1.0	1.0	MRI activity
FTY1	f	52	n.a.	n.a.	positive	-	2013	53	1.0	1.0	stable
FTY3	f	57	n.a.	n.a.	negative	GLAT	2013	98	1.5	1.5	stable
FTY4	f	53	n.a.	n.a.	positive	INF, GLAT, NAT, DMF	2014	168	3	3	stable
DMF6	f	41	2019	Aug – 2019	positive	-	07 – Aug – 2019	< 1	2.0	1.5	EDSS ↓
DMF7	f	34	2019	Dec – 2019	positive	-	13 – Jan – 2020	1	3.0	3.5	EDSS ↑
DMF8	f	22	2019	Dec – 2019	positive	-	30 – Jan – 2020	4	1.5	0.0	EDSS ↓, MRI activity
DMF9	f	48	2018	Jan – 2020	positive	-	05 – Mar – 2020	1	2.5	2.5	stable
TER1	m	32	2012	Feb – 2012	n.a.	-	13 – Oct – 2016	56	2.0	n.a.	n.a.
TER2	f	38	2011	Apr – 2015	n.a.	-	13 – Oct – 2016	18	2.0	n.a.	n.a.
TER3	m	52	2006	n.a. – 2006	n.a.	-	28 – Nov – 2016	132	2.5	n.a.	n.a.
TER6	m	47	2019	Sep – 2019	positive	-	08 – Nov – 2019	1	2.0	2.5	EDSS ↑, relapse and MRI activity
TER7	m	57	2018	Sep – 2019	positive	-	07 – Nov – 2019	3	2.0	2.0	stable
NAT1	f	20	n.a.	n.a.	positive	-	2013	1	2.5	2.5	stable
NAT2	m	35	n.a.	n.a.	positive	-	2013	4	2.5	2.5	stable
NAT3	f	22	n.a.	n.a.	positive	GLAT	2013	115	1.5	1.5	stable
NAT4	f	39	n.a.	n.a.	positive	GLAT, INF, DMF	2014	42	2.5	2.5	stable

MRI activity as defined by either Gadolinium enhancing lesions, new or enlarging T2-lesions or new T1 hypointensive lesions

*BL* baseline, *FUP6* follow-up six months, *EDSS* expanded disability status scale, *OCB* oligoclonal bands, *d* day, *y* year, *m* month, *OZA* ozanimod, *FTY* fingolimod, *DMF* dimethyl fumarate, *TER* teriflunomide, *NAT* natalizumab, *n.a*. not available, *INF* interferon, *GLAT* glatiramer acetate

↑ worsening, ↓ improved

### Distribution of sorted B cells and basic repertoire properties: no quantitative differences between MS patients and healthy controls but distinct treatment specific changes during various therapies

**Fig. 1:**
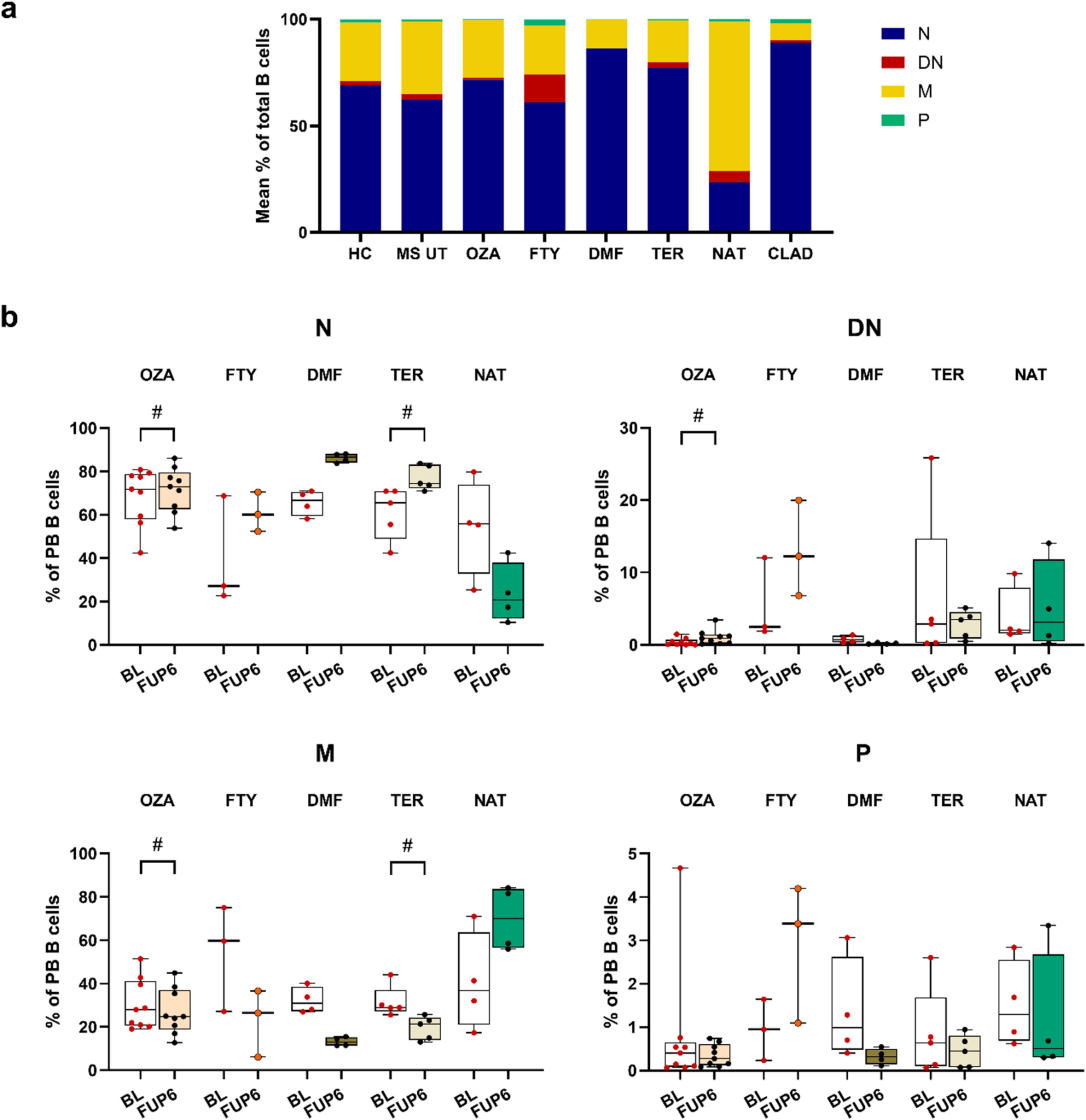
B cell subset percentages measured by flow cytometry. **a** Mean percentages of circulating B cell subsets including naive, double negative, memory B cells and plasmablasts compared across healthy controls, treatment-naïve pwMS and pwMS treated with ozanimod, fingolimod, dimethyl fumarate, teriflunomide, natalizumab and cladribine for six months. **b** Longitudinal analyses of proportional changes in B cell subsets (N, DN, M, P) within the total count of sorted peripheral blood B cells. For statistical analyses the Wilcoxon Rank-Sum paired test was performed (^#^*p* < 0.1). N, naive B cells; DN, double negative B cells; M, memory B cells; P, plasmablasts; HC, healthy control; MS UT, treatment-naïve pwMS; OZA, ozanimod; FTY, fingolimod; DMF, dimethyl fumarate; TER, teriflunomide; NAT, natalizumab; CLAD, cladribine; BL, baseline; FUP6, follow-up six months; PB, peripheral blood

The percentage distribution of naive, double negative, memory B cells, and plasmablasts relative to the total sorted amount of B cells was determined and compared across the HC group, treatment-naïve pwMS (MS UT) and the different treatment groups (Fig. 1a). A comparison of the MS UT group with the HC group showed reduced proportions of naive B cells but elevated proportions of memory B cells. Similar observations were described in literature despite the absence of statistical significance due to small sample sizes [41]. Treatment-specific effects on the distribution of B cell subsets were analyzed longitudinally (Fig. 1b). Although the statistical power was limited, we observed trends towards elevated percentages of naive B cells and decreased percentages in the memory B cell subsets following OZA and TER treatment. A similar tendency towards decreased memory B cell percentages was observed for the other treatments except NAT, which is in line with the literature [42]. A statistical trend towards elevated DN B cells was observed for OZA-treated patients.

Representative Ig repertoires were generated for all patients included in the present study with detailed data on sequence numbers and B cells provided in Suppl. Table 3. The mean number of all Ig sequences per patient was 2,860,189 (range 733,620–5,351,319) at BL, 2,590,894 (range 391,060 − 5,405,623) at FUP6, and 2,906,550 (range 143,840–6,810,474) per healthy control subject. Although we aimed to analyse equal blood volumes at each time point in the longitudinal analysis of each patient, the qualitative analysis of sorted B cells and corresponding Ig repertoire sequences was biased by differences in the initial blood volume and leukocyte counts from which we started Ig library preparation. Nevertheless, when looking at the longitudinal Ig repertoires, we observed significantly fewer Ig sequences following treatment with OZA (Suppl. Table 3). To overcome the potential bias in peripheral blood starting material, we performed extensive sub-sampling in the subsequent analyses to obtain a representative picture of Ig repertoire changes in multiple sclerosis and across different DMDs.

### Cross-sectional comparisons between healthy controls and treatment-naïve pwMS show significant qualitative repertoire changes within the DN B cells

**Fig. 2:**
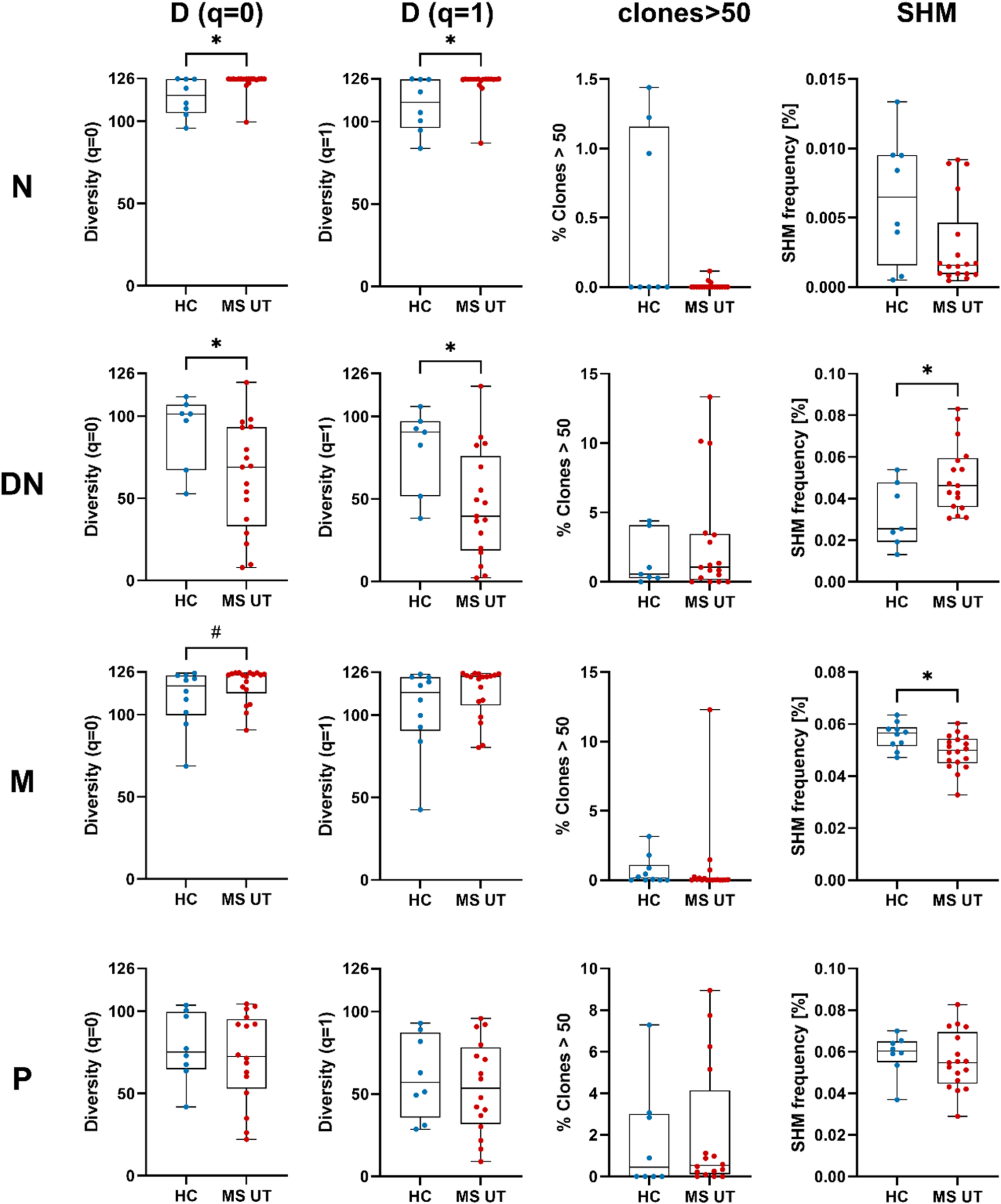
Clonal analysis for the cross-sectional comparison between healthy controls and treatment-naïve pwMS. Each column represents one metric including clonal diversity expressed as Hill numbers for q = 0 and q = 1, percentage of clones comprising more than 50 unique sequences per clone, and, somatic hypermutation frequency. Comparison data are shown per row for one B cell subset (N, DN, M, P). For statistical analyses the Mann Whitney test was performed (^#^*p* < 0.1; *p < 0.05). N, naive B cells; DN, double negative B cells; M, memory B cells; P, plasmablasts; HC, healthy control; MS UT, treatment-naïve pwMS; D, diversity; SHM, somatic hypermutation

In order to investigate whether peripheral VH repertoires exhibit differences between pwMS (*n* = 33) and healthy controls (*n* = 10), we first selected treatment-naïve pwMS (*n* = 21) out of our MS collective for further comparisons. Based on the detailed batch and outlier analysis (methods section), the FTY and NAT datasets were subsequently removed for cross-sectional comparisons due to slight differences in library generation. In the following, 18 treatment-naïve pwMS and the healthy control cohort were compared regarding the number of unique clones (diversity q = 0), the diversity of clones weighted by the clone size (diversity q = 1), the percentage of clones with more than 50 members, the frequency of somatic hypermutations (SHM) (Fig. 2), the IGHV family usage and Ig subclass distribution (Suppl. Figures 2–3). Significant differences were observed within the DN B cell population showing a lower diversity (q = 0 and q = 1), which indicates a pronounced clonal expansion within DN B cells in MS (Fig. 2, Suppl. Figures 4–5). A significant increase in diversity, although only marginal, was observed within the N B cell subset (q = 0 and q = 1), and, a trend of increased diversity was found within the M B cells (q = 0). Significantly increased somatic hypermutations were present within the DN B cell subset in pwMS. Significantly less somatic hypermutations were observed within the memory B cell subsets in pwMS which points towards less maturation processes. MS patients also showed a significantly increased IGHV2 usage in the naive B cell subset and decreased IGHV2 usage in the DN B cell subset (Suppl. Figure 2). Plasmablasts exhibited a substantially diminished IGHV2 usage, while IGHV3 usage showed a significant increase, accompanied by a further tendency toward reduced IGHV5 usage when compared to healthy controls. No significant differences were observed regarding the other B cell subsets and clonal metrics (Suppl. Figure 3).

Additional sub-analyses were conducted to validate the observed effects. No gender-specific differences were observed within the MS patient group (data not shown). However, a notable difference emerged when comparing treatment-naïve pwMS with pretreated pwMS who had previously undergone a wash-out phase prior to the initiation of the new treatment. In pretreated pwMS, clonal analysis showed a statistical trend towards reduced diversity (q = 0, q = 1) in the naive and memory B cell subset (Suppl. Figure 6). Significant differences and trends were also observed regarding IGHV family distribution (Suppl. Figure 7) and Ig subclasses (Suppl. Figure 8).

### Various treatments show qualitative effects on peripheral Ig repertoires in pwMS with a consistent reduction of clonal diversity

#### Longitudinal analysis

**Fig. 3:**
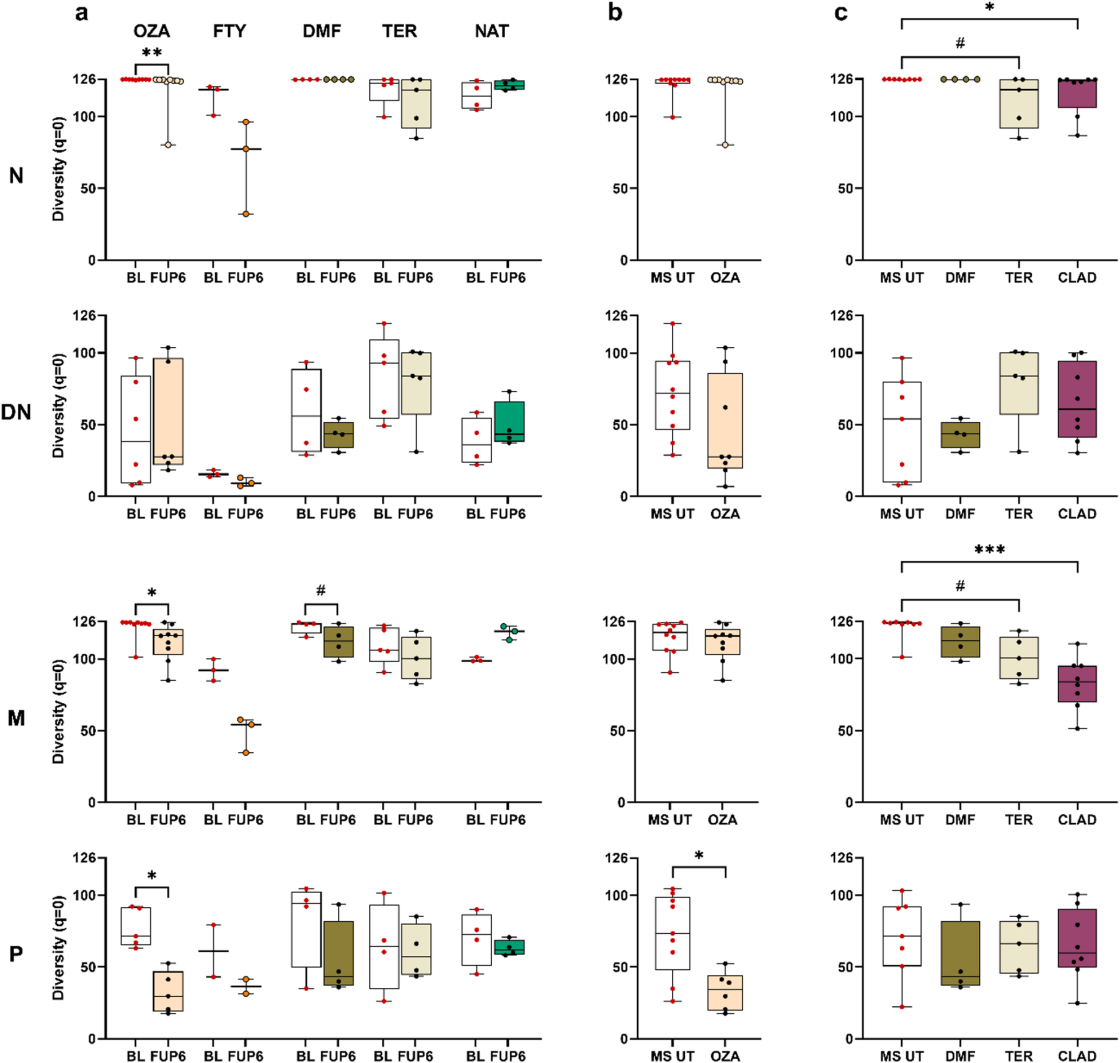
Clonal diversity (q = 0) comparison. **a** The clonal diversity is shown for each subject per treatment group (OZA, FTY, DMF, TER, NAT) and B cell subset (N, DN, M, P) and compared longitudinally between the time points (BL, FUP6). **b** Clonal diversity compared cross-sectionally between OZA-treated patients (following 6 months of treatment) and treatment-naïve pwMS (MS UT) from the other treatments (baseline). **c** Clonal diversity compared cross-sectionally between DMF-, TER- and CLAD-treated patients (following 6 months of treatment) and treatment-naïve patients (MS UT) from the OZA cohort (baseline). For statistical analyses the Wilcoxon Rank-Sum paired test in (**a**), the Mann-Whitney test in (**b**) and the Kruskal-Wallis test with Dunn’s correction in (**c**) were performed (^#^*p* < 0.1; *p < 0.05; **p < 0.01; ***p < 0.001). OZA, ozanimod; FTY, fingolimod; DMF, dimethyl fumarate; TER, teriflunomide; NAT, natalizumab; CLAD, cladribine; MS UT, treatment-naïve pwMS; BL, baseline; FUP6, follow-up six months; N, naive B cells; M, memory B cells; DN, double negative B cells; P, plasmablasts

Because the different immunomodulatory treatment regimens exert quantitative effects on the distribution of immune cells, our subsequent investigation focused on the qualitative effects of OZA, FTY, DMF, TER and NAT treatments on peripheral Ig (VH) repertoires within the different B cell subsets. To this end, longitudinal analyses were performed between baseline and following six months of treatment, encompassing the diversity at q = 0 (Fig. 3) and q = 1, the percentage of clones > 50 members, the frequency of SHM, the IGHV family usage, Ig subclass distribution, and clonal overlaps. As the statistical analyses were limited in some patient groups, the OZA-treated MS patient group was first examined, followed by the remaining treatment cohorts.

In the context of longitudinal OZA-related treatment effects, a marked decrease in diversity (q = 0, Fig. 3a) and a concomitant reduction in clonal diversity at q = 1 were observed within the naive, memory B cells, and plasmablasts (Suppl. Figures 9–11). Furthermore, we observed a significant increase in the percentage of clones > 50 clone members within the memory B cell and plasmablast populations (Suppl. Figure 12). This finding suggests activation within the naive B cell population with a decline in the overall diversity, while memory B cells and plasmablasts show a reduction in the overall number of sampled clones with certain larger, more expanded clones persisting during treatment. No significant differences were observed in the percentage of SHM (Suppl. Figure 13). Furthermore, OZA treatment resulted in a substantial decrease in the utilization of the IGHV1, 2 and 5 gene families within the memory B cell subset, while IGHV3 usage exhibited a significant increase. Conversely, the plasmablast subset exhibited an opposing effect, marked by a significant increase in IGHV2 usage and a substantial decrease in IGHV3 usage (Suppl. Figure 14). With respect to the Ig subclasses, OZA treatment resulted in a significant decrease in IgD usage and an increase in IgM usage within the naive B cell subset (Suppl. Figure 15). No significant differences were observed for the other B cell populations.

A subsequent examination of the different longitudinal treatments including FTY, DMF, TER, and NAT revealed the absence of statistically significant effects on diversity measurements for the various B cell subsets. This finding is most likely attributable to the limited sample size, particularly in the FTY and NAT treatment groups. However, consistent peripheral effects were observed across treatments with the exception of NAT. When compared to OZA, FTY treatment also demonstrated a reduction in the diversity of the naive, memory B cell, and plasmablast subsets but not in the DN B cell subsets. No discernible trends were observed in the naive and DN B cell subsets for the other treatment groups. The memory B cell population exhibited a statistical trend towards reduced diversity in patients treated with DMF; a similar tendency was also observed for TER (q = 0, Fig. 3a and q = 1, Suppl. Figure 9). A tendency towards a reduced diversity was observed in the plasmablast subset during FTY, DMF, and NAT treatment (q = 0, Fig. 3a and q = 1, Suppl. Figure 9), although this did not reach statistical significance. Additionally, the metrics encompassing somatic hypermutations, clones comprising more than 50 clone members, IGHV usage, and Ig subclass distributions exhibited statistical trends without reaching statistical significance. Further details can be found in Suppl. Figures 10–15.

#### Cross-sectional analysis

Cross-sectional Ig repertoire comparisons between treatment-naïve pwMS and healthy controls showed substantial disparities including lower diversity within the DN B cells and higher diversity within the N and M B cell subsets in pwMS as well as significant differences in IGHV gene family usage (N: IGHV2↑; DN: IGHV2↓; P: IGHV2↓, IGHV3↑, IGHV5↓ in pwMS). Given the observation of these disparities in specific Ig repertoire characteristics, cross-sectional analyses were also conducted between the different treatment groups and treatment-naïve pwMS. According to the defined patient groups we performed an analysis between OZA-treated patients (following six months of treatment) and treatment-naïve pwMS from the other treatments (baseline), and, -vice versa-, compared patients treated with DMF, TER and CLAD (following six months of treatment) to treatment-naïve patients from the OZA patient group (baseline). The significant reduction of clonal diversity (q = 0) in the longitudinal data following OZA treatment were also confirmed in the cross-sectional data set for the plasmablast subset with a trend in the naive B cell population (Fig. 3b); similar results were observed for the clonal diversity at q = 1 (Suppl. Figure 16a). No significant effects were observed for clones > 50 sequences or SHM (Suppl. Figure 16b-c). A significant underrepresentation of the IGHV5 family was observed in the memory B cells (Suppl. Figure 17). Furthermore, IgA usage was significantly increased and IgG and IgM decreased in the DN B cells, whereas IgG usage significantly increased and IgM decreased in the memory population (Suppl. Figure 18).

When looking at the cross-sectional analysis between treatment-naïve pwMS and DMF, TER and CLAD patients following six months of treatment, a significantly decreased clonality (q = 0 and q = 1) was observed in the naive and memory B cell subset in CLAD-treated patients (Fig. 3c, Suppl. Figure 19a). In contrast, the DN B cell population showed a significantly increased diversity (q = 0, q = 1) following TER treatment. Regarding bigger clones with > 50 sequences, CLAD-treated pwMS showed a significantly increased frequency in the memory B cells while statistical trends towards these larger, more expanded clones were observed in the naive and memory B cells following TER and within the plasmablast subset during DMF treatment (Suppl. Figure 19b). The usage of IGHV gene families exhibited significant disparities within the memory B cells, with a marked decline in the utilization of the IGHV1 family during DMF treatment, a significant reduction in the IGHV2 family in the TER patient group, and a notable decrease in the IGHV4 family in both TER- and CLAD-treated patients. Conversely, the expression of the IGHV3 family was considerably augmented in the memory subset subsequent to DMF and TER treatment. In the plasmablast subset, a significantly increased usage of the IGHV5 gene segments and a trend towards increased usage of the IGHV2 family was observed in the CLAD patient group (Suppl. Figure 20). A substantial impact was observed in relation to Ig subclass utilization, with a considerable decline in IgG usage during CLAD therapy, and a comparable tendency was noted in DMF-treated patients within the memory B cell subset. A decline in the utilization of the IgA subclass was also observed in the DN B cells following TER treatment (Suppl. Figure 21).

#### Clonal overlaps

**Fig. 4:**
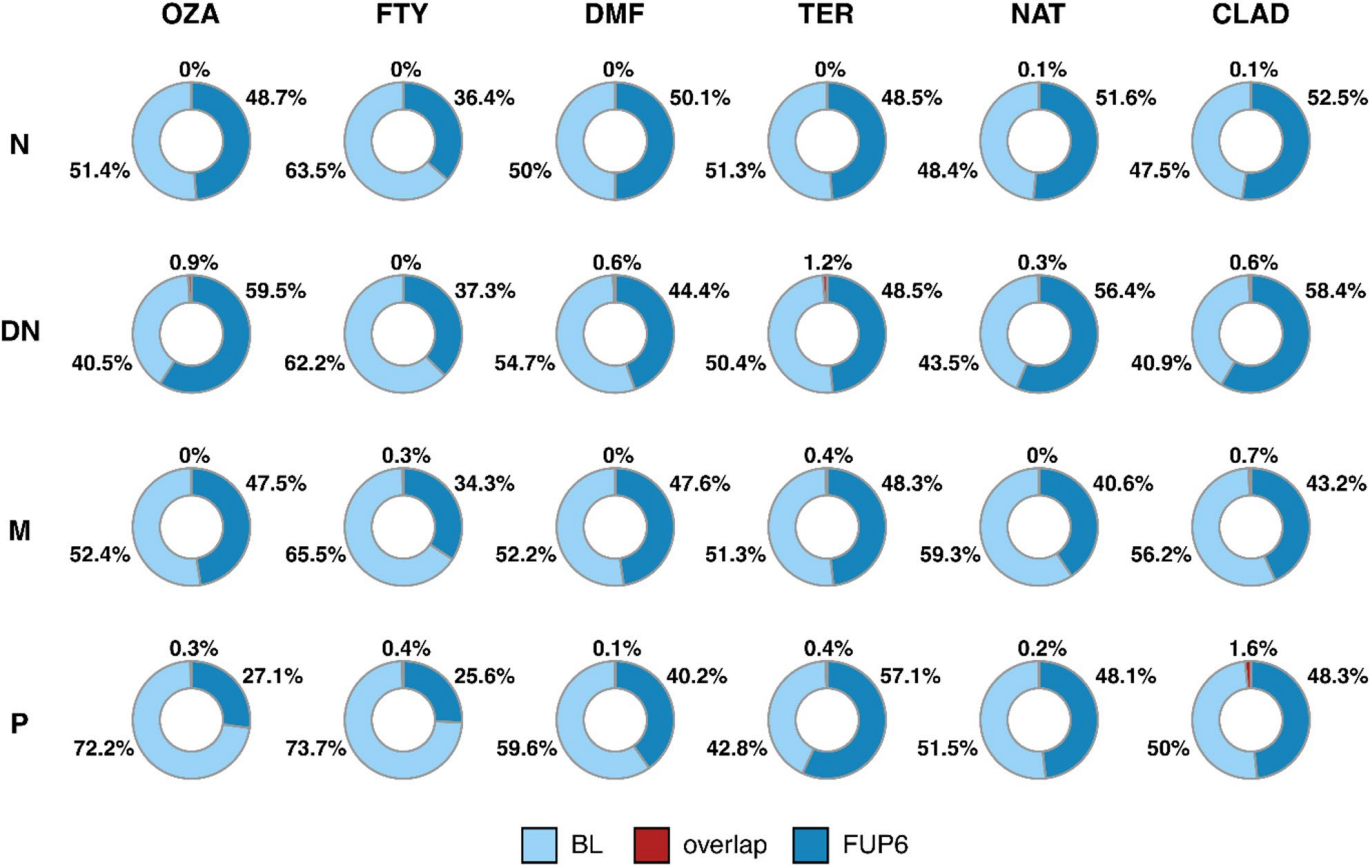
Clonal overlaps analysis. The proportion of shared clones between the baseline time point and following six months of a certain treatment is given as the mean across all subjects per individual treatment group (OZA, FTY, DMF, TER, NAT, CLAD) and for each B cell population (N, DN, M, P). The Kruskal-Wallis test with Benjamini-Hochberg correction was performed to test for a difference in clonal overlap distributions between treatments. OZA, ozanimod; FTY, fingolimod; DMF, dimethyl fumarate; TER, teriflunomide; NAT, natalizumab; CLAD, cladribine; BL, baseline; FUP6, follow-up six months; N, naïve B cells; DN, double negative B cells; M, memory B cells; P, plasmablasts

In order to further understand the clonal dynamics within the different B cell subsets during the different treatments, clonal overlaps between both time points were analyzed (Fig. 4). The extent of clonal overlap within the B cell subset between the different time-points remained limited, with less than 3% of clonal overlap observed. No statistically significant differences were observed regarding clonal overlaps across different DMD treatments.

### Normalization of IGHV gene usage following different treatments

**Fig. 5:**
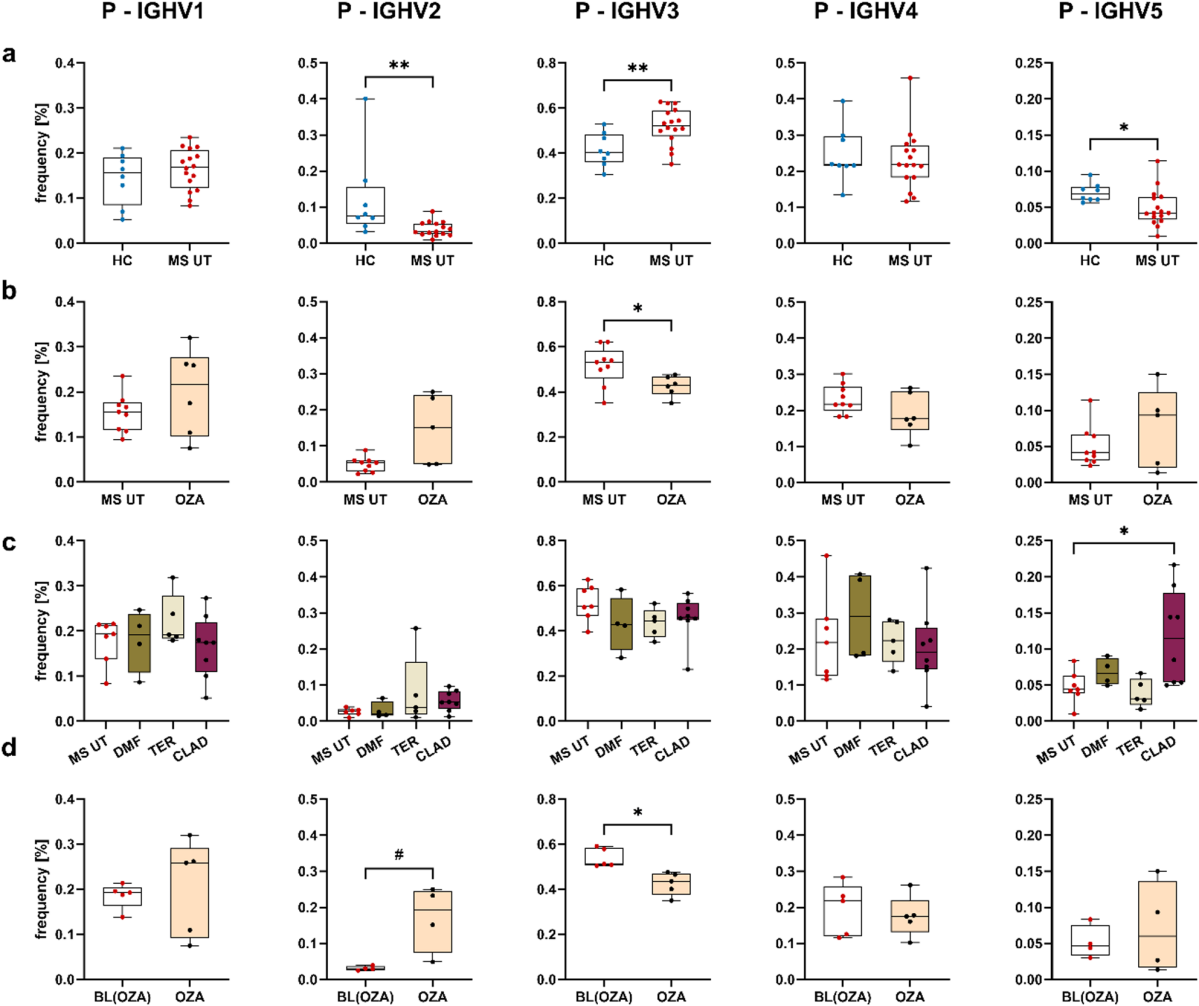
Reversal of the change in gene usage within the plasmablast population after treatment. Gene usage frequency compared cross-sectionally between **a** healthy controls and treatment-naïve pwMS, **b** OZA-treated patients (following six months of treatment) and treatment-naïve pwMS, **c** DMF, TER and CLAD-treated patients (following six months of treatment) and treatment-naïve patients from the OZA cohort (baseline) and (**d**) longitudinal comparison of gene usage frequency after six months of OZA treatment. For statistical analyses the Mann-Whitney test in (**a**) and (**b**), the Kruskal-Wallis test with Dunn’s correction in (**c**) and the Wilcoxon Rank-Sum paired test in (**d**) were performed (^#^*p* < 0.1; *p < 0.05; **p < 0.01). P, plasmablasts; HC, healthy control; MS UT, treatment-naïve pwMS; OZA, ozanimod; DMF, dimethyl fumarate; TER, teriflunomide; CLAD, cladribine; BL, baseline

Since significant Ig repertoire changes were observed in the comparison of untreated pwMS and healthy controls, we were interested whether the different treatments reverse alterations in the untreated MS Ig repertoire. The treatments did not significantly alter the clonal composition of DN B cells as seen in the untreated MS group but significantly changed the composition of IGHV usage. Most pronounced effects in treatment-naïve pwMS were seen in the plasmablast population with a decreased percentage of IGHV2 and IGHV5 families while the IGHV3 gene family was overrepresented (Fig. 5a). These alterations were partially reversed during OZA and CLAD treatment showing a statistical trend in the cross-sectional data (Fig. 5b and c), and more pronounced in the longitudinal data set. In particular, OZA treatment led to a higher percentage of IGHV2 usage and significant reduction of IGHV3 usage (Fig. 5d). Comparable trends were observed for DMF- and TER-treated patients (Fig. 5c, Suppl. Figure 14). Correlation analyses between the VH family genes and the EDSS as clinical metric showed significantly positive correlations between EDSS and IGHV2 or IGHV5 and a negative correlation with IGHV3 for the plasmablast population and the BL timepoint (Suppl. Figure 22).

### Immunoglobulin proteome analyses

**Fig. 6:**
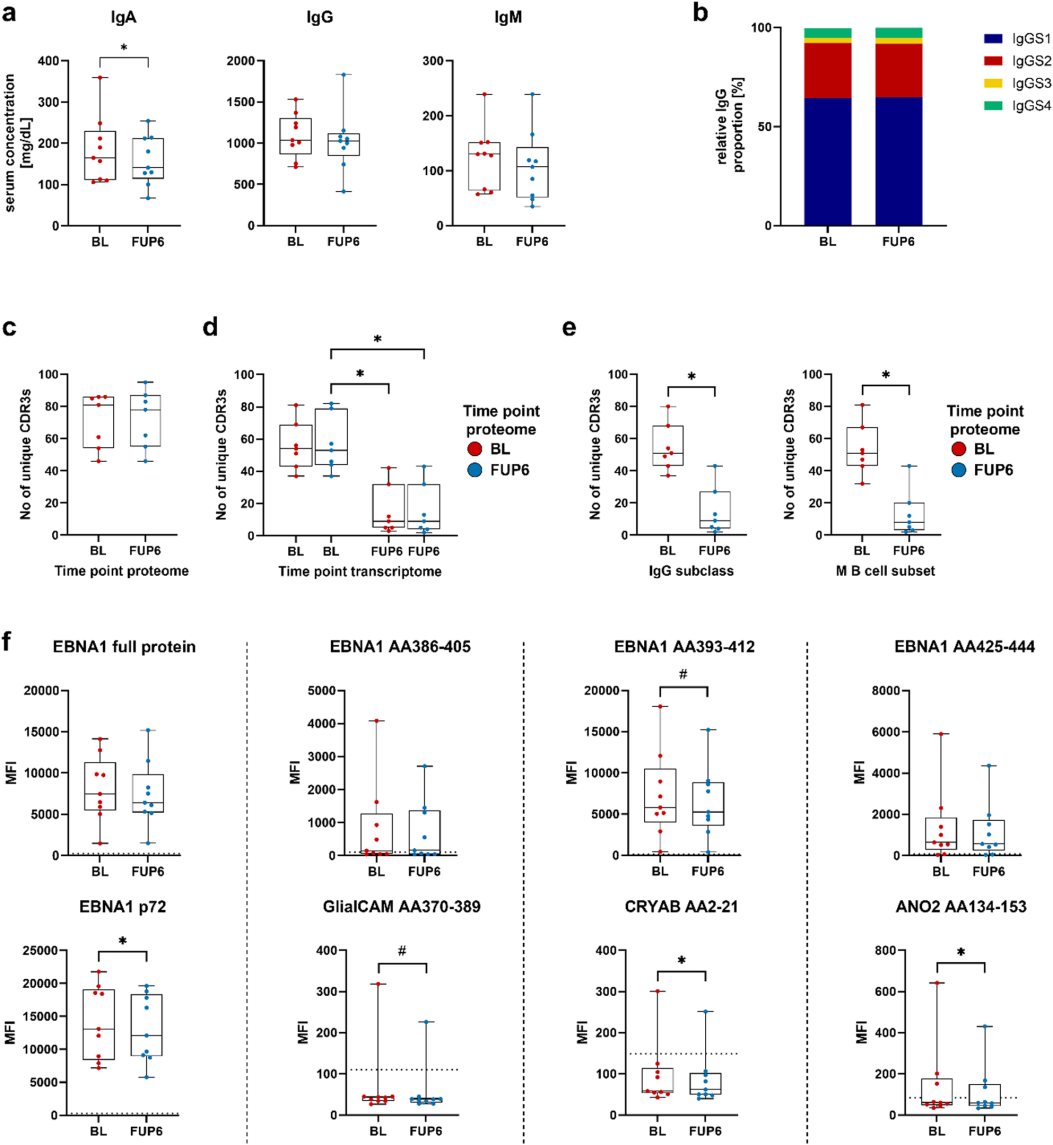
Longitudinal immunoglobulin proteome analysis for the ozanimod (OZA) patient cohort. Quantitative effects of OZA treatment on (**a**) serum IgA, IgG and IgM levels and on (**b**) IgG1-4 subclass distribution. Alignment of Ig proteome sequences to the Ig transcriptome libraries with emphasis on (**c**) the total number of recovered unique CDR3 sequences per time point (BL, FUP6), **d** matching Ig sequences within one time point and between both time points, **e** matching Ig sequences within the IgG isotype and memory B cell population. **f** Serum IgG antibody levels at baseline and after six months of OZA treatment were measured against EBNA1 (full protein, p72), specific EBNA1-peptides (AA386-405, AA393-412, AA425-444) and their respective molecular mimicry peptides GlialCAM (AA370-389), CRYAB (AA2-21) and ANO2 (AA134-153). The horizontal dotted line indicates the cut-off for IgG seropositivity. For statistical analyses the Wilcoxon Rank-Sum paired test and the Friedman-test with Dunn’s correction were performed (#*p* < 0.1; *p < 0.05). BL, baseline; FUP6, follow-up six months; CDR3, complementarity determining region-3; EBNA1, EBV nuclear antigen 1; MFI, mean fluorescence intensity

In order to understand how changes on the cellular level translate into changes of the circulating immunoglobulin repertoire we performed quantitative measurements on Ig levels and specific EBV antibody levels and also assessed the serum Ig proteome. Ig proteome-derived peptides were identified by translating each patient’s Ig transcriptome sequences into their respective protein sequence and matching peptides resulting from the tryptic digest to mass spectra. Qualitative changes within the Ig proteome repertoire were determined by aligning those peptides with the -for each B cell clone highly individual- CDR3 region, assuming that a considerable proportion of the Ig proteome in the periphery originates from a continuum of peripheral B cells migrating toward the bone marrow. Looking at the quantitative effects of OZA treatment on Ig serum levels, we observed a significant decrease in IgA levels, while IgG and IgM serum levels did not show significant changes (Fig. 6a). The distribution of IgG1-4 subclasses also demonstrated stability following OZA treatment (Fig. 6b). The total number of specific Ig proteomic peptides associated with the Ig transcriptome libraries demonstrated a stable proportion of specific Ig peptides at baseline and after six months of OZA treatment (Fig. 6c). However, when the Ig proteome peptide libraries from each time point were further aligned individually to the corresponding Ig transcriptome at baseline or six months of treatment, the number of specific Ig proteome peptides remained stable for each time point. Nevertheless, the number of specific alignments to the six-month Ig transcriptome significantly decreased, as the overall number of recovered Ig transcriptome sequences was lower after OZA treatment (Fig. 6d). This effect was most likely due to a significant decrease in the number of Ig transcriptome sequences in the memory B cell subset, as a lower number of specific Ig proteome peptides were aligned to the IgG and memory B cell sequences at the transcriptome level (Fig. 6e). Given the hypothesized role of molecular mimicry in MS and recent findings that suggest that anti-EBV nuclear antigen 1 (EBNA1) antibodies are cross-reactive against GlialCAM, CRYAB and ANO2 [43–45], we also analyzed the specific antibody response against these targets during treatment. There was a significant reduction in antibody titers against EBNA1 (p72), CRYAB and ANO2 and a statistical trend towards lower antibody titers against GlialCAM and EBNA1 (AA393-412) following OZA treatment (Fig. 6f). These longitudinal data complement the anti-EBV antibody measurements in pwMS and control groups in our recently published article [28].

Although statistical analysis was limited by the smaller number of patients, we also observed an overall stable number of unique Ig peptides aligned to the transcriptome libraries in patients treated with DMF or TER (Suppl. Figure 23a). As was the case with the OZA patient group, further alignment of the Ig proteome peptide libraries from each time point to each Ig transcriptome at baseline or after six months of treatment revealed a consistent number of Ig peptides for each time point. However, a reduced number of specific peptides were aligned to the six-month Ig transcriptome due to the reduced number of Ig transcripts following treatments (Suppl. Figure 23b). No significant effects were observed when analyzing the memory B cell subset (Suppl. Figure 23c). Furthermore, TER treatment resulted in a significantly lower antibody titer against EBNA1 (p72) and a statistical trend towards lower EBNA1 (AA425-444) titers, with no effect on the other EBV-related antibodies. The DMF treatment did not result in significant changes in antibody titers (Suppl. Figure 23d).

## Discussion

In recent years, mounting evidence has clearly established the pivotal role of B cells in the pathophysiology of multiple sclerosis [46]. The remarkable efficacy of treatments that target CD20-positive circulating B cells has unequivocally demonstrated the substantial impact of peripheral B cells on autoimmune processes in MS within the adaptive immune system [46]. Furthermore, numerous studies have demonstrated that different treatments also influence peripheral B cells [14, 47]. However, the precise characteristics of B cell repertoires and the specific mechanisms by which treatments exert their effects remain to be fully elucidated [48]. The objective of this study was to characterize the differences in the peripheral B cell repertoire between pwMS and healthy controls and to further examine the detailed effects of various treatments on the peripheral Ig repertoire on both, the transcriptome and proteome level. Despite the limited number of patients in some treatment groups, our data set contains the most comprehensive Ig transcriptomic characterization of pwMS, with more than 196,100,000 sequences analyzed in this project. We implemented bioinformatics analyses controlling for different sequencing depths to enable robust cross-sample comparisons and to provide a comprehensive picture of the changes in the immune repertoire in pwMS and the reshaped Ig repertoire properties during different MS treatments.

We observed no substantial or striking alterations in the overall composition of peripheral B cell subsets in pwMS compared to the HC group. Our detailed investigation of the Ig repertoire however revealed some significant qualitative disparities in the diversity of DN B cells, and utilization of specific IGHV gene families in the naive B cell, DN and plasmablast populations. Although these findings always have to be interpreted with caution due to artificial shifts in IGHV gene usage caused by library generation and small sample sizes, the diverse technical analyses, including strict filters for calculating repertoire metrics, and the numerous statistical methods must however be considered, as they enable robustness and comparability of the data and thus allow for biological interpretation. While the IGHV2 gene family was overrepresented in the naive B cells in pwMS, this gene family was underrepresented in the antigen-experienced DN B cell and plasmablast subsets. In plasmablasts, an overrepresentation of the IGHV3 family was detectable which is in line with the literature [23]. An overrepresentation of IGHV2 and especially IGHV4 gene segments has already been described in the analyses of CNS and CSF B cell repertoires in MS, however, the exact implications of this finding for MS pathophysiology remain unclear [6, 49–52]. In contrast, a recent study demonstrated a peripheral underrepresentation of the IGHV4-34 gene in pwMS when compared to healthy individuals, but did not differentiate between B-cell populations [23]. Interestingly, the IGHV2 and IGHV4 germlines that are also overrepresented in CSF Ig repertoires of clinically isolated syndrome (CIS) subjects, are closely related based on nucleotide sequence homology in the framework 1 and framework 3 intervals [53]. The B cells analyzed in CSF and MS lesions with the dominant IGHV4 gene usage profile are highly expanded [50, 52, 54] and are clonally related to peripheral switched memory B cells, plasmablasts, and DN B cells. In our dataset, none of these populations showed significant clonal expansion, and gene usage was evenly distributed, which is largely consistent with the literature [19]. However, the distinction from HC based on the significant differences in individual IGHV genes in plasmablasts and DN B cells, as well as the reduced diversity with increased SHM profile in DN B cells, suggests increased activity of these B-cell clones. The DN B cell subset has been shown to be involved in various autoimmune diseases such as NMOSD and systemic lupus erythematosus [55, 56] but the exact role in MS remains ambiguous [18]. It is hypothesized that DN B cells constitute an antigen experienced B cell subset evolving during extra follicular maturation processes also showing properties of antibody secreting cells [18, 56, 57]. While a clear up-regulation of DN B cells and clonal connection to CSF B cells was observed in NMOSD, the percentage of DN B cells was not significantly altered in MS in previous studies although some patients showed a tendency towards elevated values [17, 18]. The results of this study indicate that altered repertoire properties of DN B cells are present in MS, and could be indicative of a distinct autoimmune predisposition in pwMS in terms of tolerance mechanisms [15]. The study by Pérez-Saldívar and colleagues already showed that patients with multiple sclerosis, especially those experiencing a relapse, exhibited significantly reduced B cell repertoire diversity and a higher proportion of expanded clones compared to healthy controls [23]. However, it does not indicate whether, and if so, which B-cell subpopulation exhibits the significant difference. In contrast the results obtained here demonstrate unequivocally that a defined alteration in the Ig repertoire is evident, as indicated by discrepancies in clonal expansion within the DN B cell subset and the gene usage of specific B cell populations. These alterations represent a significant novel finding and may prove to be of considerable importance in the future diagnosis of multiple sclerosis, in the pathophysiological understanding of the disease, and also with regard to the efficacy of treatments.

Regarding the specific treatment effects of OZA, FTY, DMF, TER and NAT, significant qualitative changes were observed in the Ig repertoires which to a certain degree also showed redundant effects. Quantitative analyses revealed a shift toward increased frequencies of naive B cells and decreased frequencies of memory B cells, which was observed across all treatments except for NAT and is in line with previous studies [14]. Counteracting treatment effects were observed in NAT treated patients, which demonstrated lower frequencies of naive B cells and elevated frequencies of memory B cells compared to healthy controls. Ig repertoires also showed significant differences or at least a trend in the naive and memory B cell populations with an increased diversity in pwMS compared to HC, even though the differences were small. After all treatments, except for NAT, significant changes in the naive and memory B cell populations with a decreased diversity and thus reverse to the findings between HC and MS UT were observed. Although naive B cells per definition should not show somatic hypermutations, activated B cells with a naive phenotype and evidence of clonal expansion to a certain degree have been described in autoimmune diseases [58]. The lower diversity in the naive B cell pool following different treatments indicates that treatments might change the naive B cell subset towards a more “activated” naive B cell population with a certain degree of clonality in this subset, possibly through a higher proliferative activity which goes along with an increased quantitative fraction of naive B cells [59]. Memory B cells also showed a lower diversity with a tendency towards expanded clones with more than 50 members pointing towards an overall reduction of clones in the memory B cell pool. The aforementioned effects were observed in the longitudinal and cross-sectional analyses for all treatment groups, with the exception of the NAT group which highlights an important role of memory B cells in the pathogenesis of multiple sclerosis. A review by Longbrake and Cross concludes that, due to the depleting effects of DMTs on memory B cells, the B-cell repertoire is remodeled toward an immature and regulatory B-cell repertoire, which in turn has a protective effect [60]. This hypothesis can be extended by the fact that the targeted depletion of antigen-experienced B cell clones is directly related to therapeutic efficacy. A comparable effect was also observed in relation to the plasmablast population, with a reduced number of clones following diverse treatment modalities. This suggests that B cell maturation and persistence may be impeded by all treatments, with the exception of NAT, resulting in an altered antigen-experienced B cell pool. Of note, even the pretreated cohort displayed persisting changes of clonal diversity in the N and M subsets. This implies that the effects of previous DMDs on the repertoire level might outlast far beyond a 30 day washout period or the observed “normalization” of standard blood count parameters. With regard to the impact on IGHV family usage, particularly OZA, but also other treatments like for example CLAD, there was a reversal of the effects on the IGHV2 and 3 families towards patterns seen in healthy individuals. It remains uncertain whether these observed neutralizing effects result from treatment-induced neutralization or represent a generic immune reconstitution. CLAD induces pronounced depletion of peripheral B cells, particularly within the memory B-cell compartment [26, 46], whereas the peripheral B-cell reduction under OZA is primarily due to the retention of approximately 60–70% of lymphocytes within lymph nodes. After these treatments, new naive B cells are generated through immune reconstitution and subsequently released into the periphery, where they can be activated and expanded upon antigen exposure. Given that CLAD does not achieve complete B-cell depletion, and S1PR modulation during OZA therapy affects B-cell subsets differentially, the peripheral B-cell pool likely comprises a heterogeneous mix of persistent pre-existing memory or plasmablast populations alongside newly generated B cells. The limited clonal overlap observed, potentially influenced by a restrictive analytical pipeline, hampers the identification of shared clones pre- and post-therapy, suggesting that normalization of gene usage following OZA or CLAD therapy predominantly reflects generic immune reconstitution processes [46, 61]. However, if any clonal overlap is detected, it is mainly found among activated B-cell subsets, particularly plasmablasts, with the most pronounced effect under CLAD, indicating a small fraction of pre-existing B cells with a preference for IGHV2 and IGHV5 genes, but not IGHV3, that were not affected by depletion or S1PR modulation. In summary, the examined treatments have been demonstrated to exert distinct quantitative and qualitative effects on the peripheral B cell repertoire. These effects include activation within the naive B cell pool, as well as a reduction of clones in antigen-experienced cells, with a particular focus on the memory B cell population. In this context, it is also essential from a translational perspective to determine whether the clonal dynamics observed before or after therapy are mirrored in clinical outcomes. Correlation analyses between the EDSS and the clonal metrics did show significant correlations between EDSS and VH germline usage in some cases which were not maintained after multiple testing correction; this was likely due to the limited number of patients (Suppl. Figure 22).

In addition to the distinct transcriptomic alterations, it was of particular interest how these changes translate into alterations with regard to the Ig proteome. Consequently, the Ig peptides were overlapped with the corresponding Ig transcriptome sequences for each patient and time point, in order to identify specific overlaps in the CDR3 region. An overall stable overlap between the Ig proteome and transcriptome was observed during longitudinal analyses of patients treated with OZA, DMF and TER. This finding is consistent with previous studies on CLAD-treated patients [26]. The constant overlap of specific peptides after six months of treatment with baseline Ig transcripts indicates that no significant qualitative changes in the Ig proteome occur during a short-term treatment phase. However, when looking at the quantitative changes we did observe significantly lower Ig levels for some subtypes indicating that less Ig’s are produced under certain treatments which is in line with other studies [62]. Although the exact role of EBV in MS has not been fully established yet, longitudinal antibody titers were studied against EBNA1 peptides and potentially cross-reactive antibodies against GlialCAM, ANO2 and CRYAB [43–45]. We also observed lower antibody titers against certain EBNA1 peptides and also GlialCAM, ANO2 and CRYAB following treatment with OZA and in part TER which also highlights quantitative treatment effects. The decline in these molecular mimicry-related IgG antibody titers and the parallel decrease of Ig proteome-transcriptome matches, which can be attributed to reduced IgG transcripts in the memory B cell population after therapy, suggest that the various immunomodulatory therapies lead to a selective quantitative suppression of antibody titers in serum. However, it should be noted that the pathophysiological role of these antibodies remains to be demonstrated. Consequently, their relevance in multiple sclerosis is currently uncertain, especially in light of conflicting results [28]. To summarize, quantitative effects on Ig levels were evident in the longitudinal analysis while the qualitative composition of Ig peptides remained unchanged at the Ig proteome level.

Our study has certain implications for the pathophysiological role of peripheral B cells in MS. The data clearly indicate that treatment-naïve pwMS show certain alterations in terms of clonal expansion of B cell subsets (DN B cells) and also IGHV gene usage. Although the exact relevance of these findings for autoimmune processes remains unclear, these repertoire changes can be interpreted in the context of a misguided immune response during MS. The DN B cell population has been postulated to evolve through extra-follicular maturation processes thus possibly escaping certain regulatory mechanisms in germinal center reactions. In this context, polyreactive naive B cells have been postulated in MS that might evolve by altered tolerance mechanisms [63]. Since it has been shown that activated naive B cells develop into antigen presenting cells via a double negative B cell phenotype [58], our findings on DN B cells could be interpreted in the context of a misguided B cell maturation. The preferential usage of certain IGHV gene families has also been reported in certain autoimmune diseases (e.g. VH4 overrepresentation in systemic lupus [64]) and might be associated with preferential binding of the BCR to certain antigens [18]. The overrepresentation of IGHV2 in the naive B cell subset might be associated with such mechanisms although CNS/CSF B cells showed an overrepresentation of mainly IGHV4 and in second place IGHV2 [6]. However, the underrepresentation of the IGHV2 gene in DN B cell subsets and the plasma cell population does not necessarily support this explanation. Alternatively, it could indicate the preferential maturation and homing of these B cell clonotypes within the CNS niche. The frequent usage of IGHV3 gene segments may indicate a distinct role in the preferential recognition of specific antigens, as previously observed within the framework of molecular mimicry between EBNA1 and GlialCAM [43]. Although the precise nature of this association remains to be fully elucidated and has been questioned, IGHV3 gene segments are potentially implicated in the development of autoimmune reactivity and/or in the persistence of EBV infection in multiple sclerosis patients [65]. Indirect effects on MS pathophysiology can be also drawn from the treatment effects on the peripheral B cell repertoire. This study in line with previous studies shows that not only the number of memory B cells and the percentage distribution of memory B cells is significantly reduced under various treatments [14, 47, 66–69] but also the number of clones is significantly reduced in this B cell subset [26, 70]. Although certain changes in the frequency and Ig repertoire properties were also seen for the plasmablasts (especially in the OZA group) the effects on the memory B cell population seem to be more pronounced. Given that the Epstein-Barr virus establishes latency primarily in the memory B cell population [15] it can be hypothesized that the memory B cell pool and not the plasmablast B cell subset as the major antibody producing effector population might be of special importance for MS pathophysiology. In their role of antigen presenting cells, memory B cells could possibly stimulate and prime autoreactive T cells and thus feed autoimmune circuits [15, 71].

Several limitations of our study have to be discussed. Despite the fact that the patient collective of 33 pwMS measured at two time points appears to represent the most extensive and detailed peripheral B cell repertoire data in MS, the inherent heterogeneity of human samples and the low number of patients receiving certain treatments constrained the statistical analysis in some groups. This also implies certain limitations regarding the number of corrections that can be applied in multiple tests, as statistical significance may be reduced or even lost, as observed in our dataset (data not shown). Next-generation sequencing continues to be the preferred method for the assessment of extensive repertoires of bulk-sorted B cells [72]. However, it should be noted that Ig repertoire analyses merely provide a snapshot of the B cell repertoires at a specific time point [73]. Furthermore, the results may be subject to bias due to the process of repertoire reconstruction, as the efficiency of sequencing differs between populations. Despite the implementation of safeguards such as high-fidelity PCR and UMIs, the presence of sequencing errors and over-amplification of transcripts (particularly in DN B cells and plasmablasts) may lead to the creation of artificial, over-represented B cell clones. In order to overcome the aforementioned bias, a rigorous bioinformatics protocol was employed, with five consensus count sequences required to represent one Ig sequence to address the issue, and, 100 unique Ig sequences per cell type and sample necessary to represent a clonal repertoire. This became particularly clear when looking at the SHM in the naive B cell population, as sequencing errors could be significantly excluded using a stricter filter. Conversely, the rigidity of the definition of the transcriptome resulted in a restriction of the overlap between the repertoires of the Ig proteome and the transcriptome. Moreover, a substantial subsampling strategy (200 repetitions) was implemented [30] enabling the comparison of clonal parameters that are not affected by different sequencing depths and also allows for proper inter-cohort comparison. From a practical perspective this additionally addresses the fact that, whilst it is preferable to use the same amount of starting material, this is not always feasible during sample collection and processing. Another relevant aspect in the context of overlap analyses between transcriptome and proteome is the difficulty to identify and align those proteome peptides with the transcriptome that actually originate from peripheral B cells. In fact, the majority of serum Ig’s are released from long-lived plasma cells resident in the bone marrow and a transient proportion of serum antibodies is produced by short-lived plasma cells or plasmablasts in the periphery [74, 75]. Thus, the targeted proteome signal from peripheral B cells could be obscured by the proteome signal from bone marrow plasma cells. However, there is a continuum of constantly maturing B cells in the peripheral blood system from memory B cells developing into plasmablasts, which migrate and secrete antibodies into the periphery, and can differentiate into long-lived plasma cells [74, 75]. Given the minimally invasive sampling method, an overlap between the blood cell transcriptome and serum proteome represents the best possible approximation for transferring changes from the transcriptome level to the proteome level. But, in order to characterize these changes at the proteome level even more distinctly, it would have also been beneficial to analyze data from other timepoints beyond six months. It is also important to acknowledge that, despite the utilization of an equivalent experimental workup and repertoire generation protocol, batch effects between different patient cohorts were evident during the statistical analysis of the samples. To minimize this problem already at the computational methods stage, techniques such as a subsampling strategy and the stringent filters mentioned above were used for the clonal calculations. To further counteract the batch effect issue, we applied several statistical methods such as outlier detection [76] and variance analyses with and without controlling for co-factors and variables, but could not confirm and thus not exclude any samples as technical outliers. Nevertheless, we decided to exclude the FTY and NAT due to deviations in library generation protocol in order to ensure for cross-sectional analysis of samples with equal conditions. Validating our data using independent cohorts to corroborate our findings would have been meaningful, but it was not feasible in the context of this work and presents technical hurdles such as different B cell sorting strategies or other sequencing protocols, which in turn complicates comparison. Data acquisition in technical replicates would be another strategy for this. Within this project technical triplicates were included for proteome analysis; however, mass sequencing samples did not undergo technical replicates due to feasibility constraints. In this particular context, batch effects may represent a challenge that is not fully appreciated in a variety of multi-center and big data projects. Nevertheless, we were able to assess representative Ig transcriptome and proteome libraries from all patients and observed consistent results throughout the different treatment groups.

## Conclusions

In conclusion, the present study provides compelling initial evidence for a significantly altered peripheral blood Ig repertoire in people with MS. The study strengthens the notion that peripheral B-cell organization mirrors immune activity within the CNS, and that the therapeutic efficacy of DMTs may partly arise from remodeling peripheral clonal networks. This aligns conceptually with findings from anti-CD20 therapies and investigations of B-cell trafficking. Nevertheless, the practical significance of these observations remains uncertain. But, these findings could be of significant importance with regard to diagnostic tests and could prompt machine learning approaches aimed at discriminating peripheral MS B cell repertoires from those of healthy controls. The findings from the various treatment groups once more demonstrate a consistent effect on memory B cells during different treatments and also demonstrate a certain normalization of Ig repertoires, especially in the plasmablast B cell subset. Further studies are required to achieve a comprehensive understanding of the distinct changes in peripheral B cell repertoires, define specific disease-driving B cell subsets and to elucidate the mechanisms by which disease-driving B cells contribute to MS pathogenesis - ultimately paving the way for more targeted therapeutic strategies.

## Supplementary Information

Supplementary Material 1.

## Data Availability

The data represented in the study will be made available upon publication. R and Python scripts used for bioinformatical downstream analyses are available at https:/github.com/qbic-projects/MS-treatment-study. Additional data can be found in supplementary information files. Raw sequencing data will be uploaded to the German Human Genome-Phenome Archive (GHGA) and the proteomics raw data will be available via proteomics identifications database (PRIDE), both upon publication. The mass spectrometry proteomics data have been deposited to the ProteomeXchange Consortium via the PRIDE partner repository with the dataset identifier PXD075641. The data generated in this study are available via controlled access in the German Human Genome-Phenome Archive (GHGA, data.ghga.de) under the GHGA Accession https://urldefense.com/v3/__https://data.ghga.de/study/GHGAS33650434537165__;!!NLFGqXoFfo8MMQ!pXUWpegDXrrYUtkm2SgcWwMPi0NI_of8cMJleR7_ZstVy4rIelHnmx6PL7Bun1145ib1oGCcVQ1SRK3QnF_R75Ca-crm4zDR_lMT8gtfXQ$. Further details, including the data access policy for the study, can be found there.

## Abbreviations

AA: Amino acid
ANO2: Anoctamin 2
BL: Baseline
CDR3: Complementarity determining region-3
CIS: Clinically isolated syndrome
CLAD: Cladribine
CNS: Central nervous system
CRYAB: Alpha-crystallin B
CSF: Cerebrospinal fluid
DDA: Data-dependent acquisition
DIA: Data-independent acquisition
DMD: Disease modifying drug
DMF: Dimethyl fumarate
DN: Double negative
EBNA1: EBV nuclear antigen 1
EBV: Epstein-Barr virus
EDSS: Expanded disability status scale
FTY: Fingolimod
FUP6: Follow-up six months
FUP12: Follow-up 12 months
GlialCAM: Glial cell adhesion molecule
HC: Healthy control
IFN: Interferon
Ig: Immunoglobulin
LC-MS/MS: Liquid chromatography tandem mass spectrometry
M: Memory
MS: Multiple sclerosis
MS UT: Treatment-naïve / untreated pwMS
N: Naive
NAT: Natalizumab
NGS: Next generation sequencing
OZA: Ozanimod
P: Plasmablast
PB: Peripheral blood
PBMC: Peripheral blood mononuclear cell
PBS: Phosphate buffered saline
PCR: Polymerase chain reaction
SHM: Somatic hypermutation frequency
TER: Teriflunomide
TUM: Technical University of Munich
TÜ: Tübingen
UMI: Unique molecular identifier
US: United States
V(D)J: Variable-diversity-joining
VH: Variable heavy chain
