# Supplementary material for: Extensive peripheral immunoglobulin repertoire analyses in people with multiple sclerosis reveal disease-specific signatures and distinct treatment effects of disease modifying drugs
Source: J Neuroinflammation. 2026 Apr 14;23:128. doi: 10.1186/s12974-026-03735-0 (PMC13088472; doi:10.1186/s12974-026-03735-0)
Supplement: Supplementary file 1 — Supplementary Material 1. [file 12974_2026_3735_MOESM1_ESM.docx]

**Supplementary Materials**

**Supplementary Methods, Supplementary Tables & Supplementary Figures**

**Supplementary Methods:**

*Variance analyses and outlier detection*

Numerous technical sub-analyses were implemented to consider co-factors and technical bias as far as possible for the main analyses. Multiple linear regression was performed by merging the data (variables: diversity q = 0 and q = 1, clones > 50 sequences, SHM, IGHV1-5 gene family usage, IgD and IgM or IgA, IgG and IgM isotype frequencies) for all BL pwMS and HC subjects but analyzing each cell subset (N, DN, M, P) separately (data not shown). Variance analysis was based on adding information on the co-factors for each subject: sex (male, female), location (TÜ, TUM or US) and condition (MS treatment-naïve, MS pretreated or healthy control). Location as a co-factor showed an impact on selected group comparisons, although not consistent between the cell subsets. The co-factor condition showed a stronger impact, especially the data between treatment-naïve and pretreated pwMS differed significantly. This was confirmed when performing group comparisons for all data variables between treatment-naïve and pretreated pwMS (Suppl. Figures 6-8). Based on this data, the pretreated pwMS were excluded from the untreated MS patient cohort (MS UT, ≙ treatment-naïve) for following cross-sectional group comparisons. Outlier analysis was performed on the merged data sets per cell subset to investigate whether the found variances were attributable to the entire batch or only affected individual patient/ subject samples. In the first step, the ROUT method was used to search for outliers in each population and for each variable (Suppl. Table 5). The largest bias was measured within the naive B-cell population. For each subject, the number of times identified as outliers was determined and the samples with an outlier count ≥ 3 were preselected (FTY1, FTY3, FTY4, NAT1, NAT2, TER1, CLAD2, CLAD6, HC1, HC5, HC9, HC10). In the next steps, variance analysis using principal component analysis (PCA, Suppl. Figure 24) and hierarchical clustering (Suppl. Figure 25) were performed for each B-cell population and finally the preselected outliers were compared between the three methods. From the preselected samples the patient/ subject samples FTY4, NAT1, NAT2, TER1, CLAD2, CLAD6, HC5, HC9 and HC10 showed the most common hits as outliers, though not consistent between all B cell subsets. Besides, further outliers for PCA and hierarchical clustering method were present, but not consistent between all variance analysis methods. Based on these inconsistent results no outliers were confirmed, however it was decided to exclude at least the US data set (FTY, NAT) from all cross-sectional analyses due to adjusted protocols when generating the TUM and TÜ data in order to keep the group conditions as consistent as possible. Since it was not possible to confirm the remaining preselected outliers (TER1, CLAD2, CLAD6, HC5, HC9 and HC10) the group comparison HC vs. MS UT was used to examine how robust the results are if outliers (healthy control or treatment-naïve: TER1, HC5, HC9 and HC10) are excluded or not (Figure 2, Suppl. Figure 26). Excluding outliers did not affect the outcome substantially which is why these samples were kept when performing the final cross-sectional analyses. To still consider location as a co-factor for this group comparison an analysis of covariance (ANCOVA) was performed in case of all significant changes or at least trends in change (corrected p-values in Suppl. Table 6) and a simple visualization by discriminating between TUM and TÜ processed samples was added (Suppl. Figure 27). No samples or cohorts were excluded when performing longitudinal analyses. A summary of all included samples per group comparison can be found in supplementary data (Suppl. Tables 7-11).

**Supplementary Tables:**

**Suppl. Table 1: Clinical data for additional comparison groups**

| ID | Sex | Age  BL | First reported relapse (Y) | Date of diagnosis (M – Y) | CSF status (OCB) at diagnosis | Previous therapies | Date study inclusion  (D – M – Y) | Disease duration (M) | EDSS  BL | EDSS  FUP6 | Disease activity during 6 M of treatment | EDSS FUP12 | Disease activity during 12M of treatment |
| --- | --- | --- | --- | --- | --- | --- | --- | --- | --- | --- | --- | --- | --- |
| CLAD1 | f | 36 | 2010 | n.a.-2010 | positive | GLAT, FTY | 07 – Feb – 2018 | 97 | 3.5 | 3 | EDSS improved | 2.5 | EDSS improved |
| CLAD2 | f | 39 | 1994 | n.a.-1996 | n.a. | IFN, GLAT, AZA, FTY | 04 –Jun – 2018 | 269 | 2 | 2 | stable | 3.5 | EDSS ↑ |
| CLAD3 | f | 29 | 2007 | n.a.-2007 | n.a. | IFN, NAT, DMF, FTY, NAT | 27 – Nov – 2018 | 144 | 3.5 | 3.5 | stable | 3.5 | stable |
| CLAD4 | f | 23 | 2014 | Sep-14 | positive | DMF | 14 – Jan – 2019 | 52 | 2 | 2 | stable | 2 | stable |
| CLAD5 | f | 24 | 2016 | n.a.-2016 | positive | DMF | 13 – May – 2019 | 40 | 2 | 2.5 | EDSS ↑ | 2.5 | stable |
| CLAD6 | f | 29 | 2017 | May-18 | positive | GLAT | 18 –Jun – 2019 | 14 | 2 | 1 | EDSS ↓ | 1 | stable |
| CLAD7 | f | 27 | 2016 | Apr-16 | positive | IFN | 02 – Jul – 2019 | 40 | 1.5 | 1.5 | stable | 1.5 | stable |
| CLAD8 | m | 21 | 2018 | May-18 | positive | - | 07 – Jul – 2019 | 15 | 2 | 1.5 | EDSS ↓ | 1.5 | stable |
| HC1 | m | 32 | - | - | - | - | 04 – Dec – 2018 | - | - | - | - | - | - |
| HC2 | f | 55 | - | - | - | - | 07 – Oct – 2019 | - | - | - | - | - | - |
| HC3 | f | 32 | - | - | - | - | 11 – Nov – 2020 | - | - | - | - | - | - |
| HC4 | m | 46 | - | - | - | - | 28 – May – 2020 | - | - | - | - | - | - |
| HC5 | m | 33 | - | - | - | - | 2017 | - | - | - | - | - | - |
| HC6 | m | 35 | - | - | - | - | 2017 | - | - | - | - | - | - |
| HC7 | f | 33 | - | - | - | - | 2016 | - | - | - | - | - | - |
| HC8 | m | 33 | - | - | - | - | 2016 | - | - | - | - | - | - |
| HC9 | m | 36 | - | - | - | - | 2016 | - | - | - | - | - | - |
| HC10 | m | 36 | - | - | - | - | 2016 | - | - | - | - | - | - |

BL, baseline; FUP6, follow-up six months; EDSS, expanded disability status scale; CSF, cerebrospinal fluid; OCB, oligoclonal bands; D, day; Y, year; M, month; FUP12, follow-up 12 months; CLAD, cladribine; HC, healthy control; n.a., not available; GLAT, glatirameracetate; FTY, fingolimod; INF, interferon; AZA, azathioprine; NAT, natalizumab; DMF, dimethyl fumarate; ↑, worsening; ↓, improved.

**Suppl. Table 2: Overview of transcriptome and proteome data acquisition**

| Subject | Location | Sort Data | Ig transcriptome | Ig proteome | Serum Ig levels | Anti-EBV serum IgG levels | Additional information |
| --- | --- | --- | --- | --- | --- | --- | --- |
| OZA1 | TÜ | available | available | available | available | available |  |
| OZA2 | TÜ | available | available | available | available | available |  |
| OZA3 | TÜ | available | available | available | available | available |  |
| OZA4 | TÜ | available | available | available | available | available |  |
| OZA5 | TÜ | available | available | available | available | available |  |
| OZA6 | TÜ | available | available | available | available | available |  |
| OZA7 | TÜ | available | available | available | available | available |  |
| OZA8 | TÜ | available | available | available | available | available |  |
| OZA9 | TÜ | available | available | available | available | available |  |
| FTY1 | US | available | available | n.a. | n.a. | n.a. | Re-analysed with new pipeline |
| FTY3 | US | available | available | n.a. | n.a. | n.a. | Re-analysed with new pipeline |
| FTY4 | US | available | available | n.a. | n.a. | n.a. | Re-analysed with new pipeline |
| DMF6 | TÜ | available | available | available | n.a. | available |  |
| DMF7 | TÜ | available | available | available | n.a. | available |  |
| DMF8 | TÜ | available | available | available | n.a. | available |  |
| DMF9 | TÜ | available | available | available | n.a. | available |  |
| TER1 | TUM | available | available | available | n.a. | available |  |
| TER2 | TUM | available | available | available | n.a. | available |  |
| TER3 | TUM | available | available | available | n.a. | available |  |
| TER6 | TÜ | available | available | available | n.a. | available |  |
| TER7 | TÜ | available | available | available | n.a. | available |  |
| NAT1 | US | available | available | n.a. | n.a. | n.a. | Re-analysed with new pipeline |
| NAT2 | US | available | available | n.a. | n.a. | n.a. | Re-analysed with new pipeline |
| NAT3 | US | available | available | n.a. | n.a. | n.a. | Re-analysed with new pipeline |
| NAT4 | US | available | available | n.a. | n.a. | n.a. | Re-analysed with new pipeline |
| CLAD1 | TÜ | available | available | available | n.a. | n.a. | Re-analysed with new pipeline |
| CLAD2 | TÜ | available | available | available | n.a. | n.a. | Re-analysed with new pipeline |
| CLAD3 | TÜ | available | available | available | n.a. | n.a. | Re-analysed with new pipeline |
| CLAD4 | TÜ | available | available | available | n.a. | n.a. | Re-analysed with new pipeline |
| CLAD5 | TÜ | available | available | available | n.a. | n.a. | Re-analysed with new pipeline |
| CLAD6 | TÜ | available | available | available | n.a. | n.a. | Re-analysed with new pipeline |
| CLAD7 | TÜ | available | available | available | n.a. | n.a. | Re-analysed with new pipeline |
| CLAD8 | TÜ | available | available | available | n.a. | n.a. | Re-analysed with new pipeline |
| HC1 | TÜ | available | available | n.a. | n.a. | n.a. |  |
| HC2 | TÜ | available | available | n.a. | n.a. | n.a. |  |
| HC3 | TÜ | available | available | n.a. | n.a. | n.a. |  |
| HC4 | TÜ | available | available | n.a. | n.a. | n.a. |  |
| HC5 | TUM | available | available | n.a. | n.a. | n.a. |  |
| HC6 | TUM | available | available | n.a. | n.a. | n.a. |  |
| HC7 | TUM | available | available | n.a. | n.a. | n.a. |  |
| HC8 | TUM | available | available | n.a. | n.a. | n.a. |  |
| HC9 | TUM | available | available | n.a. | n.a. | n.a. |  |
| HC10 | TUM | available | available | n.a. | n.a. | n.a. |  |

OZA, ozanimod; FTY, fingolimod; DMF, dimethyl fumarate; TER, teriflunomide; NAT, natalizumab; CLAD, cladribine; HC, healthy control; TÜ, Tübingen; TUM, Technical University of Munich; US, United States; Ig, immunoglobulin; EBV, Epstein-Barr virus; n.a., not available.

**Suppl. Table 3: Sorted cells and sequence count**

| Subject | Time point | Cell Count | | | | | Sequence Count | | | | |
| --- | --- | --- | --- | --- | --- | --- | --- | --- | --- | --- | --- |
|  |  | **N** | **DN** | **M** | **P** | **Total Count** | **N** | **DN** | **M** | **P** | **Total Count** |
| OZA1 | BL | 140243 | 4808 | 170004 | 15433 | 330488 | 11700 | 962 | 8616 | 2797 | 24075 |
|  | FUP6 | 23725 | 262 | 19835 | 295 | 44117 | 10862 | 357 | 5738 | 682 | 17639 |
| OZA2 | BL | 632366 | 594 | 257844 | 4849 | 895653 | 12005 | 364 | 9330 | 238 | 21937 |
|  | FUP6 | 15332 | 739 | 5350 | 90 | 21511 | 10028 | 849 | 2356 | 417 | 13650 |
| OZA3 | BL | 277382 | 773 | 74603 | 2693 | 355451 | 33331 | 496 | 14169 | 810 | 48806 |
|  | FUP6 | 63101 | 826 | 9334 | 62 | 73323 | 19857 | 1428 | 6832 | 1272 | 29389 |
| OZA4 | BL | 158165 | 151 | 40143 | 805 | 199264 | 41109 | 257 | 3312 | 242 | 44920 |
|  | FUP6 | 33086 | 550 | 11316 | 334 | 45286 | 11712 | 663 | 3640 | 796 | 16811 |
| OZA5 | BL | 352271 | 2013 | 100045 | 671 | 455000 | 49863 | 1 | 10937 | 694 | 61495 |
|  | FUP6 | 34786 | 712 | 9319 | 246 | 45063 | 2149 | 358 | 923 | 728 | 4158 |
| OZA6 | BL | 500079 | 7552 | 333258 | 879 | 841768 | 28574 | 563 | 11868 | 370 | 41375 |
|  | FUP6 | 37175 | 93 | 23382 | 97 | 60747 | 6687 | 167 | 2125 | 40 | 9019 |
| OZA7 | BL | 404214 | 127 | 157531 | 411 | 562283 | 13206 | 146 | 3304 | 497 | 17153 |
|  | FUP6 | 81099 | 126 | 25737 | 92 | 107054 | 16738 | 378 | 172 | 55 | 17343 |
| OZA8 | BL | 197174 | 1143 | 149578 | 1867 | 349762 | 7608 | 198 | 2871 | 242 | 10919 |
|  | FUP6 | 87661 | 324 | 48500 | 373 | 136858 | 6621 | 63 | 3340 |  | 10024 |
| OZA9 | BL | 236279 | 52 | 55803 | 191 | 292325 | 24280 | 7 | 5038 | 57 | 29382 |
|  | FUP6 | 111949 | 1206 | 23141 | 221 | 136517 | 7577 | 379 | 466 | 126 | 8548 |
| FTY1 | BL | 47500 | 3870 | 156000 | 477 | 207847 | 13043 | 2757 | 1317 | 3360 | 20477 |
|  | FUP6 | 3459 | 451 | 2420 | 277 | 6607 | 7194 | 327 | 14070 | 5010 | 26601 |
| FTY3 | BL | 134000 | 4905 | 53000 | 3200 | 195105 | 14311 | 2514 | 15008 | 1821 | 33654 |
|  | FUP6 | 3520 | 715 | 1550 | 64 | 5849 | 27495 | 1583 | 12490 | 4319 | 45887 |
| FTY4 | BL | 8250 | 3630 | 18000 | 287 | 30167 | 6002 | 2835 | 21855 | 2416 | 33108 |
|  | FUP6 | 12786 | 3624 | 1111 | 614 | 18135 | 2427 | 1643 | 1572 | 25 | 5667 |
| DMF6 | BL | 272623 | 6416 | 188239 | 1899 | 469177 | 31278 | 653 | 19547 | 4316 | 55794 |
|  | FUP6 | 607825 | 1569 | 102740 | 807 | 712941 | 22044 | 2262 | 17395 | 3340 | 45041 |
| DMF7 | BL | 556325 | 2275 | 220448 | 5496 | 784544 | 23174 | 3160 | 12757 | 2387 | 41478 |
|  | FUP6 | 646305 | 959 | 120033 | 4183 | 771480 | 21801 | 2924 | 9971 | 1874 | 36570 |
| DMF8 | BL | 202855 | 1341 | 79021 | 8948 | 292165 | 15978 | 3168 | 10286 | 4206 | 33638 |
|  | FUP6 | 491253 | 1793 | 64156 | 2111 | 559313 | 13620 | 1802 | 10280 | 2555 | 28257 |
| DMF9 | BL | 284426 | 4075 | 150349 | 5684 | 444534 | 26120 | 1676 | 18113 | 2875 | 48784 |
|  | FUP6 | 231545 | 277 | 30306 | 662 | 262790 | 20700 | 4312 | 17754 | 4208 | 46974 |
| TER1 | BL | 120000 | 73000 | 82000 | 7341 | 282341 | 3991 | 4367 | 13454 | 2491 | 24303 |
|  | FUP6 | 74000 | 5336 | 24000 | 983 | 104319 | 21510 | 2004 | 11548 | 4061 | 39123 |
| TER2 | BL | 63000 | 3400 | 29000 | 743 | 96143 | 13593 | 938 | 7223 | 1209 | 22963 |
|  | FUP6 | 60000 | 2830 | 9521 | 322 | 72673 | 8936 | 2216 | 13464 | 4111 | 28727 |
| TER3 | BL | 180000 | 7294 | 65000 | 1621 | 253915 | 13216 | 2093 | 3114 | 1 | 18424 |
|  | FUP6 | 150000 | 7000 | 43000 | 1352 | 201352 | 22222 | 4434 | 10330 | 7208 | 44194 |
| TER6 | BL | 79215 | 415 | 62823 | 198 | 142651 | 11389 | 3655 | 10978 | 3273 | 29295 |
|  | FUP6 | 386582 | 5881 | 69245 | 329 | 462037 | 24862 | 1520 | 9807 | 3124 | 39313 |
| TER7 | BL | 275181 | 850 | 111700 | 257 | 387988 | 21070 | 3050 | 14087 | 3363 | 41570 |
|  | FUP6 | 368869 | 2490 | 128793 | 421 | 500573 | 6918 | 9811 | 2297 | 9445 | 28471 |
| NAT1 | BL | 80000 | 2100 | 58800 | 1270 | 142170 | 8810 | 6610 | 11107 | 10529 | 37056 |
|  | FUP6 | 75704 | 968 | 353570 | 2958 | 433200 | 24127 | 7803 | 23430 | 4897 | 60257 |
| NAT2 | BL | 38400 | 6810 | 22250 | 1970 | 69430 | 18632 | 5748 | 29512 | 1924 | 55816 |
|  | FUP6 | 189000 | 5670 | 249000 | 1327 | 444997 | 23507 | 3040 | n.a. | 4718 | 31265 |
| NAT3 | BL | 27000 | 2010 | 75400 | 1790 | 106200 | 2241 | 14319 | 11240 | 34731 | 62531 |
|  | FUP6 | 52200 | 24900 | 421200 | 1650 | 499950 | 4973 | 3621 | 33156 | 2754 | 44504 |
| NAT4 | BL | 129000 | 3570 | 28000 | 1000 | 161570 | 16590 | 614 | 20097 | 1791 | 39092 |
|  | FUP6 | 14400 | 8400 | 35000 | 2000 | 59800 | 6355 | 15097 | 24874 | 6110 | 52436 |
| CLAD1 | BL | 151185 | 1892 | 37849 | 3065 | 193991 | 2144 | 2013 | 3639 | 2258 | 10054 |
|  | FUP6 | 247218 | 2818 | 10897 | 7194 | 268127 | 615 | 2900 | 1626 | 3493 | 8634 |
| CLAD2 | BL | 186422 | 3527 | 16627 | 1460 | 208036 | 707 | 5647 | 10117 | 9834 | 26305 |
|  | FUP6 | 415031 | 2300 | 5567 | 2106 | 425004 | 18382 | 3684 | 7379 | 7574 | 37019 |
| CLAD3 | BL | 47169 | 288 | 21773 | 1026 | 70256 | 7637 | 6431 | 8045 | 16885 | 38998 |
|  | FUP6 | 168184 | 1102 | 8720 | 1544 | 179550 | 11707 | 313 | 4801 | 2731 | 19552 |
| CLAD4 | BL | 10096 | 169 | 18165 | 283 | 28713 | 3189 | 3680 | 1834 | 1378 | 10081 |
|  | FUP6 | 18485 | 345 | 2503 | 1004 | 22337 | 307 | 4097 | 1851 | 2725 | 8980 |
| CLAD5 | BL | 248067 | 6269 | 50850 | 3033 | 308219 | 6568 | 3393 | 4511 | 5711 | 20183 |
|  | FUP6 | 185307 | 1687 | 6012 | 1333 | 194339 | 7948 | 4433 | 4547 | 5666 | 22594 |
| CLAD6 | BL | 141796 | 5766 | 52661 | 389 | 200612 | 3196 | 5799 | 6008 | 4590 | 19593 |
|  | FUP6 | 126949 | 1906 | 8915 | 313 | 138083 | 11172 | 7506 | 5280 | 3392 | 27350 |
| CLAD7 | BL | 105149 | 2824 | 13813 | 1373 | 123159 | 7091 | 4891 | 7937 | 6497 | 26416 |
|  | FUP6 | 61801 | 626 | 2107 | 440 | 64974 | 5659 | 5406 | 6419 | 1804 | 19288 |
| CLAD8 | BL | 194662 | 3111 | 93591 | 3235 | 294599 | 11548 | 4237 | 7320 | 9532 | 32637 |
|  | FUP6 | 68023 | 2731 | 30990 | 5887 | 107631 | 3069 | 5521 | 4123 | 4989 | 17702 |
| HC1 | BL | 416985 | 5171 | 237784 | 14212 | 674152 | 27 | 63 | 1232 | 260 | 1582 |
| HC2 | BL | 195567 | 451 | 38990 | 260 | 235268 | 2886 | 16 | 530 | 4 | 3436 |
| HC3 | BL | 724771 | 8969 | 345303 | 4345 | 1083388 | 7235 | 469 | 3911 | 310 | 11925 |
| HC4 | BL | 1244469 | 4669 | 281699 | 996 | 1531833 | 9 | 9 | 6895 | 132 | 7045 |
| HC5 | BL | 710000 | 12000 | 130000 | 11000 | 863000 | 16625 | 13081 | 7293 | 551 | 37550 |
| HC6 | BL | 270000 | 14000 | 150000 | 4414 | 438414 | 31252 | 11820 | 8342 | 18 | 51432 |
| HC7 | BL | 530000 | 23000 | 270000 | 40000 | 863000 | 2724 | 9578 | 10423 | 2255 | 24980 |
| HC8 | BL | 65000 | 916 | 15000 | 1009 | 81925 | 2213 | 21769 | 1787 | 2959 | 28728 |
| HC9 | BL | 36745 | 1225 | 29465 | 1246 | 68681 | 18094 | 7464 | 21837 | 8267 | 55662 |
| HC10 | BL | 44505 | 4147 | 23063 | 2081 | 73796 | 12131 | 9728 | 26185 | 11004 | 59048 |

N, naive B cells; DN, double-negative B cells; M, memory B cells; P, plasmablasts; OZA, ozanimod; FTY, fingolimod; DMF, dimethyl fumarate; TER, teriflunomide; NAT, natalizumab; CLAD, cladribine; HC, healthy control; BL, baseline; FUP6, follow-up six months; n.a., not available. Sequence counts in light grey indicate samples/ B cell subsets that were excluded from the analyses. **Suppl. Table 4: Assessment of clinical follow-up after 12 months of treatment**

| **ID** | **EDSS FUP12** | **Disease activity during 12m of treatment** |
| --- | --- | --- |
| **OZA1** | 1.5 | stable |
| **OZA2** | 1.5 | stable |
| **OZA3** | 1.0 | stable |
| **OZA4** | 0.0 | stable |
| **OZA5** | n.a. | n.a. |
| **OZA6** | 1.5 | stable |
| **OZA7** | 1.5 | EDSS↑ |
| **OZA8** | n.a. | n.a. |
| **OZA9** | n.a. | Relapse and MRI activity |
| **FTY1** | n.a. | n.a. |
| **FTY3** | n.a. | n.a. |
| **FTY4** | n.a. | n.a. |
| **DMF6** | 1.5 | stable |
| **DMF7** | 3.5 | stable |
| **DMF8** | 0.0 | stable |
| **DMF9** | 2.5 | stable |
| **TER1** | n.a. | n.a. |
| **TER2** | n.a. | n.a. |
| **TER3** | n.a. | n.a. |
| **TER6** | n.a. | n.a. |
| **TER7** | 2.5 | EDSS ↑ |
| **NAT1** | n.a. | n.a. |
| **NAT2** | n.a. | n.a. |
| **NAT3** | n.a. | n.a. |
| **NAT4** | n.a. | n.a. |

EDSS, expanded disability status scale; m, month; FUP12, follow-up 12 months; OZA, ozanimod; FTY, fingolimod; DMF, dimethyl fumarate; TER, teriflunomide; NAT, natalizumab; n.a., not available; ↑, worsening; ↓, improved; MRI activity as defined by either Gadolinum enhancing lesions, new or enlarging T2-lesions or new T1 hypointensive lesions.

**Suppl. Table 5: Outlier Identification using the ROUT method**

| ID | Condition | location | sex | d q0 | d q1 | Clones > 50 sequences (%) | IGHV1 (%) | IGHV2 (%) | IGHV4 (%) | IGHV5 (%) | IgD (%) | IgM (%) | IgA (%) | Outlier count |
| --- | --- | --- | --- | --- | --- | --- | --- | --- | --- | --- | --- | --- | --- | --- |
| OZA1 | MS UT | TÜ | f | - | - | - | - | - | - | - | - | - | - | **0** |
| OZA2 | MS UT | TÜ | m | - | - | - | - | - | - | - | - | - | - | **0** |
| OZA3 | MS UT | TÜ | m | - | - | - | - | - | - | - | - | - | - | **0** |
| OZA4 | MS UT | TÜ | f | - | - | - | - | - | - | - | - | - | - | **0** |
| OZA5 | MS pre-TR | TÜ | m | - | - | - | - | - | - | - | - | - | - | **0** |
| OZA6 | MS UT | TÜ | f | - | - | - | - | - | - | - | - | - | - | **0** |
| OZA7 | MS UT | TÜ | f | - | - | - | - | N: 0.29 | - | - | - | - | - | **1** |
| OZA8 | MS UT | TÜ | f | - | - | - | - | - | - | - | - | - | - | **0** |
| OZA9 | MS UT | TÜ | m | - | - | - | - | - | - | - | - | - | - | **0** |
| FTY1 | MS UT | US | f | - | - | N: 0.27; DN: 31.25 | - | M: 0.19 | - | - | - | - | - | **3** |
| FTY3 | MS pre-TR | US | f | - | - | N: 0.29; M: 1.98 | - | - | - | DN: 0.36 | - | - | - | **3** |
| FTY4 | MS pre-TR | US | f | N: 100.61 | N: 89.78 | N: 0.83; M: 2.64; P: 9.52 | - | - | - | DN: 0.33 | - | - | - | **6** |
| NAT1 | MS UT | US | f | N: 108.13 | N: 98.50 | N: 0.21; DN: 24.14 | - | - | - | - | - | - | - | **4** |
| NAT2 | MS UT | US | m | N: 104.72 | N: 95.21 | N: 0.70; M: 2.56 | - | - | - | - | - | - | - | **4** |
| NAT3 | MS pre-TR | US | f | - | - | P: 11.03 | - | - | - |  | - | - | - | **1** |
| NAT4 | MS pre-TR | US | f | - | - | M: 1.83 | - | - | - | DN: 0.50 | - | - | - | **2** |
| DMF6 | MS UT | TÜ | f | - | - | P: 8.95 | - | - | - | - | - | - | - | **1** |
| DMF7 | MS UT | TÜ | f | - | - | - | - | - | - | - | - | - | - | **0** |
| DMF8 | MS UT | TÜ | f | - | - | - | - | - | - | - | - | - | - | **0** |
| DMF9 | MS UT | TÜ | f | - | - | - | - | - | - | - | - | - | - | **0** |
| TER1 | MS UT | TUM | m | N: 99.59 | N: 87.02 | N: 0.12; M: 12.29 |  | - | - | - | - | - | - | **4** |
| TER2 | MS UT | TUM | f | - | - | N: 0.05 |  | - | - | - | - | - | - | **1** |
| TER3 | MS UT | TUM | m | - | - | - | - | - | - | - | - | M: 0.61 | - | **1** |
| TER6 | MS UT | TÜ | m | - | - | - | - | - | - | - | - | - | - | **0** |
| TER7 | MS UT | TÜ | m | - | - | M: 1.46 |  | - | - | - | - | - | - | **1** |
| CLAD1 | MS pre-TR | TÜ | f | - | - | - | - | - | - | - | - | - | - | **0** |
| CLAD2 | MS pre-TR | TÜ | f | N: 69.51 | N: 20.60 | N: 0.27 | - | - | N: 0.72 | - | N: 0.00 | N: 1.00 | - | **6** |
| CLAD3 | MS pre-TR | TÜ | f | - | - | - | - | - | - | - | - | - | - | **0** |
| CLAD4 | MS pre-TR | TÜ | f | - | - | - | - | N: 0.25 | - | - | - | - | - | **1** |
| CLAD5 | MS pre-TR | TÜ | f | - | - | - | - | - | - | - | - | - | M: 0.09 | **1** |
| CLAD6 | MS pre-TR | TÜ | f | N: 55.01 | N: 36.65 | N: 3.68; P: 9.68 | - | - | - | P: 0.21 | - | - | - | **5** |
| CLAD7 | MS pre-TR | TÜ | f | - | - | P: 11.02 | - | - | - | - | - | - | - | **1** |
| CLAD8 | MS UT | TÜ | m | - | - | - | - | - | - | N: 0.12 | - | - | - | **1** |
| HC1 | HC | TÜ | m | - | - | - | M: 0.54 | M: 0.19; P: 0.17 | - | - | - | - | - | **3** |
| HC2 | HC | TÜ | f | - | - | - | - | M: 0.36 | - | - | - | - | - | **1** |
| HC3 | HC | TÜ | f | - | - | - | - | - | - | - | - | - | M: 0.64 | **1** |
| HC4 | HC | TÜ | m | - | - | - | - | - | - | - | - | - |  | **0** |
| HC5 | HC | TUM | m | N: 107.80 | N: 100.52 | N: 0.96 | - | - | - | - | - | - | P: 1.00 | **5** |
| HC6 | HC | TUM | m | - | - | - | - | - | - | - | - | - | - | **0** |
| HC7 | HC | TUM | f | - | - | - | - | - | - | - | - | - | - | **0** |
| HC8 | HC | TUM | m | - | - | - | - | P: 0.40 | - | - | - | - | - | **1** |
| HC9 | HC | TUM | m | N: 104.14 | N: 94.78 | N: 1.22 M: 1.80 | - | - | - | - | - | - | - | **4** |
| HC10 | HC | TUM | m | N: 95.99 | N: 83.81 | N: 1.44 M: 3.15 | - | - | - | - | - | - | - | **4** |

OZA, ozanimod; FTY, fingolimod; NAT, natalizumab; DMF, dimethyl fumarate; TER, teriflunomide; CLAD, cladribine; HC, healthy control; MS UT, MS untreated/treatment-naïve; MS pre-TR, MS pretreated; TÜ, Tübingen; US, United States; TUM, Technical University of Munich; f, female; m, male; d, diversity; N, naive B cells; DN, double negative B-cells, M, memory B-cells; P, plasmablasts. Empty cells (-) indicate, that the measured/calculated sample value of the specific category lies within the normal range. Samples for which explicit values are given were identified as outliers in the specific categories via the ROUT method. Samples with an increased outlier count (≥ 3) are highlighted in orange.**Suppl. Table 6: Comparison of p-values from statistical testings before and after correction for co-factors for the comparison HC vs. MS UT**

|  | q0 | q1 | SHM | IGHV2 | IGHV3 | IGHV5 | correction |
| --- | --- | --- | --- | --- | --- | --- | --- |
| N | 0,026 | 0,026 | 0,129 | 0,008 | 0,115 | 0,807 | no |
|  | 0,469 | 0,521 |  | 0,122 |  |  | condition+location |
|  | 0,503 | 0,554 |  | 0,097 |  |  | condition+location+condition*location |
| DN | 0,034 | 0,016 | 0,034 | <0,001 | 0,455 | 0,175 | no |
|  | 0,125 | 0,071 | 0,021 | 0,006 |  |  | condition+location |
|  | 0,093 | 0,048 | 0,016 | 0,017 |  |  | condition+location+condition*location |
| M | 0,099 | 0,133 | 0,018 | 0,824 | 0,689 | 0,724 | no |
|  | 0,817 |  | 0,028 |  |  |  | condition+location |
|  | 0,606 |  | 0,034 |  |  |  | condition+location+condition*location |
| P | 0,61 | 0,569 | 0,49 | 0,002 | 0,007 | 0,019 | no |
|  | 0,536 |  |  | 0,096 | 0,004 | 0,019 | condition+location |
|  | 0,743 |  |  | 0,115 | 0,01 | 0,02 | condition+location+condition*location |

For the cross-sectional comparison HC vs. MS UT the Mann Whitney test was performed. Before correcting for the co-factors condition and location the metrics diversity q = 0 and q = 1, SHM frequency, and, IGHV2, IGHV3 and IGHV5 gene usage showed significant differences between the comparison groups within at least one B cell subset (N, DN, M, P). For all these metrics p-values are shown for each B cell subset before correcting for the co-factors condition ad location. Corrected p-values after analysis of covariance with either correcting for the single co-factors (condition + location) or in addition their interaction (condition + location + condition * location) are shown for all metrics with a significant p-value before test correction and in addition for all diversity q = 0 results. HC, healthy control; MS UT, treatment-naïve pwMS; N, naive B cells; DN, double negative B-cells, M, memory B-cells; P, plasmablasts.

**Suppl. Table 7: Overview on the sample number for the cross-sectional comparison HC vs. MS UT**

| Group | N | DN | M | P |
| --- | --- | --- | --- | --- |
| HC | **n = 8**  **(+2 didn’t pass n > 100 sequences)**  CTRL4 ≙ HC2  CTRL5 ≙ HC3  Patient1 ≙ HC5  Patient2 ≙ HC6  Patient3 ≙ HC7  Patient4 ≙ HC8  Patient5 ≙ HC9  Patient6 ≙ HC10 | **n = 7**  **(+3 didn’t pass n > 100 sequences)**  CTRL5 ≙ HC3  Patient1 ≙ HC5  Patient2 ≙ HC6  Patient3 ≙ HC7  Patient4 ≙ HC8  Patient5 ≙ HC9  Patient6 ≙ HC10 | **n = 10**  CTRL3 ≙ HC1  CTRL4 ≙ HC2  CTRL5 ≙ HC3  CTRL6 ≙ HC4  Patient1 ≙ HC5  Patient2 ≙ HC6  Patient3 ≙ HC7  Patient4 ≙ HC8  Patient5 ≙ HC9  Patient6 ≙ HC10 | **n = 8**  **(+2 didn’t pass n > 100 sequences)**  CTRL3 ≙ HC1  CTRL5 ≙ HC3  CTRL6 ≙ HC4  Patient1 ≙ HC5  Patient3 ≙ HC7  Patient4 ≙ HC8  Patient5 ≙ HC9  Patient6 ≙ HC10 |
| MS UT | **n = 18**  **(+3 outlier)**  OZA1  OZA2  OZA3  OZA4  OZA6  OZA7  OZA8  OZA9  DMF6  DMF7  DMF8  DMF9  TER1  TER2  TER3  TER6  TER7  CLAD8 | **n = 17**  **(+3 outlier, +1 didn’t pass n > 100 sequences)**  OZA1  OZA2  OZA3  OZA4  OZA6  OZA7  OZA8  DMF6  DMF7  DMF8  DMF9  TER1  TER2  TER3  TER6  TER7  CLAD8 | **n = 18**  **(+3 outlier)**  OZA1  OZA2  OZA3  OZA4  OZA6  OZA7  OZA8  OZA9  DMF6  DMF7  DMF8  DMF9  TER1  TER2  TER3  TER6  TER7  CLAD8 | **n = 16**  **(+3 outlier, +2 didn’t pass n > 100 sequences)**  OZA1  OZA2  OZA3  OZA4  OZA6  OZA7  OZA8  DMF6  DMF7  DMF8  DMF9  TER1  TER2  TER6  TER7  CLAD8 |

The samples FTY1, NAT1, NAT2 were removed as outliers. Samples not passing the threshold of minimum 100 sequences (n = 126) during bootstrapping approach used to calculate diversity within one B cell subset could not be considered for comparison within the respective B cell subset. HC, healthy control; MS UT, treatment-naïve pwMS; N, naive B cells; DN, double negative B-cells, M, memory B-cells; P, plasmablasts; CTRL, control; OZA, ozanimod; FTY, fingolimod; NAT, natalizumab; DMF, dimethyl fumarate; TER, teriflunomide; CLAD, cladribine.

**Suppl. Table 8: Overview on the sample number for the cross-sectional comparison MS UT vs. MS pre-TR**

| Group | N | DN | M | P |
| --- | --- | --- | --- | --- |
| MS UT | **n = 18**  **(+3 outlier)**  OZA1  OZA2  OZA3  OZA4  OZA6  OZA7  OZA8  OZA9  DMF6  DMF7  DMF8  DMF9  TER1  TER2  TER3  TER6  TER7  CLAD8 | **n = 17**  **(+3 outlier****, +1 didn’t pass n > 100 sequences)**  OZA1  OZA2  OZA3  OZA4  OZA6  OZA7  OZA8  DMF6  DMF7  DMF8  DMF9  TER1  TER2  TER3  TER6  TER7  CLAD8 | **n = 18**  **(+3 outlier)**  OZA1  OZA2  OZA3  OZA4  OZA6  OZA7  OZA8  OZA9  DMF6  DMF7  DMF8  DMF9  TER1  TER2  TER3  TER6  TER7  CLAD8 | **n = 16**  **(+3 outlier, +2 didn’t pass n > 100 sequences)**  OZA1  OZA2  OZA3  OZA4  OZA6  OZA7  OZA8  DMF6  DMF7  DMF8  DMF9  TER1  TER2  TER6  TER7  CLAD8 |
| MS pre-TR | **n = 8**  **(+4 outlier)**  CLAD1  CLAD2  CLAD3  CLAD4  CLAD5  CLAD6  CLAD7  OZA5 | **n = 7**  **(+4 outlier, +1 didn’t pass n > 100 sequences)**  CLAD1  CLAD2  CLAD3  CLAD4  CLAD5  CLAD6  CLAD7 | **n = 8**  **(+4 outlier)**  CLAD1  CLAD2  CLAD3  CLAD4  CLAD5  CLAD6  CLAD7  OZA5 | **n = 8**  **(+4 outlier)**  CLAD1  CLAD2  CLAD3  CLAD4  CLAD5  CLAD6  CLAD7  OZA5 |

The samples FTY1, NAT1, NAT2 were removed as outliers. Samples not passing the threshold of minimum 100 sequences (n = 126) during bootstrapping approach used to calculate diversity within one B cell subset could not be considered for comparison within the respective B cell subset. MS UT, treatment-naïve pwMS; MS pre-TR; pretreated pwMS; N, naive B cells; DN, double negative B-cells, M, memory B-cells; P, plasmablasts; OZA, ozanimod; FTY, fingolimod; NAT, natalizumab; DMF, dimethyl fumarate; TER, teriflunomide; CLAD, cladribine.

**Suppl. Table 9: Overview on the sample number for the cross-sectional comparison MS UT vs. OZA-treated**

| Group | N | DN | M | P |
| --- | --- | --- | --- | --- |
| MS UT  (BL DMF, TER, CLAD) | **n = 10**  **(+3 outlier)**  DMF6  DMF7  DMF8  DMF9  TER1  TER2  TER3  TER6  TER7  CLAD8 | **n = 10**  **(+3 outlier)**  DMF6  DMF7  DMF8  DMF9  TER1  TER2  TER3  TER6  TER7  CLAD8 | **n = 10**  **(+3 outlier)**  DMF6  DMF7  DMF8  DMF9  TER1  TER2  TER3  TER6  TER7  CLAD8 | **n = 9**  **(+3 outlier, +1 didn’t pass n > 100 sequences)**  DMF6  DMF7  DMF8  DMF9  TER1  TER2  TER6  TER7  CLAD8 |
| OZA | **n = 9**  OZA1  OZA2  OZA3  OZA4  OZA5  OZA6  OZA7  OZA8  OZA9 | **n = 8**  **(+1 didn’t pass n > 100 sequences)**  OZA1  OZA2  OZA3  OZA4  OZA5  OZA6  OZA7  OZA9 | **n = 9**  OZA1  OZA2  OZA3  OZA4  OZA5  OZA6  OZA7  OZA8  OZA9 | **n = 6**  **(+3 didn’t pass n > 100 sequences)**  OZA1  OZA2  OZA3  OZA4  OZA5  OZA9 |

The samples FTY1, NAT1, NAT2 were removed as outliers. All treatment-naïve pwMS from the OZA-cohort were removed for the MS UT cohort to avoid longitudinal comparison with the OZA-treated cohort. Samples not passing the threshold of minimum 100 sequences (n = 126) during bootstrapping approach used to calculate diversity within one B cell subset could not be considered for comparison within the respective B cell subset. FTY, fingolimod; NAT, natalizumab; MS UT, treatment-naïve pwMS; BL, baseline; DMF, dimethyl fumarate; TER, teriflunomide; CLAD, cladribine; OZA, ozanimod; N, naive B cells; DN, double negative B-cells; M, memory B-cells; P, plasmablasts.

**Suppl. Table 10: Overview on the sample number for the cross-sectional comparison MS UT vs. DMF-, TER- and CLAD-treated**

| Group | N | DN | M | P |
| --- | --- | --- | --- | --- |
| MS UT (BL OZA) | **n = 8**  OZA1  OZA2  OZA3  OZA4  OZA6  OZA7  OZA8  OZA9 | **n = 7**  **(+1 didn’t pass n > 100 sequences)**  OZA1  OZA2  OZA3  OZA4  OZA6  OZA7  OZA8 | **n = 8**  OZA1  OZA2  OZA3  OZA4  OZA6  OZA7  OZA8  OZA9 | **n = 7**  **(+1 didn’t pass n > 100 sequences)**  OZA1  OZA2  OZA3  OZA4  OZA6  OZA7  OZA8 |
| DMF | **n = 4**  DMF6  DMF7  DMF8  DMF9 | **n = 4**  DMF6  DMF7  DMF8  DMF9 | **n = 4**  DMF6  DMF7  DMF8  DMF9 | **n = 4**  DMF6  DMF7  DMF8  DMF9 |
| TER | **n = 5**  TER1  TER2  TER3  TER6  TER7 | **n = 5**  TER1  TER2  TER3  TER6  TER7 | **n = 5**  TER1  TER2  TER3  TER6  TER7 | **n = 5**  TER1  TER2  TER3  TER6  TER7 |
| CLAD | **n = 8**  CLAD1  CLAD2  CLAD3  CLAD4  CLAD5  CLAD6  CLAD7  CLAD8 | **n = 8**  CLAD1  CLAD2  CLAD3  CLAD4  CLAD5  CLAD6  CLAD7  CLAD8 | **n = 8**  CLAD1  CLAD2  CLAD3  CLAD4  CLAD5  CLAD6  CLAD7  CLAD8 | **n = 8**  CLAD1  CLAD2  CLAD3  CLAD4  CLAD5  CLAD6  CLAD7  CLAD8 |

Only all treatment-naïve pwMS from the OZA-cohort were included for the MS UT cohort to avoid longitudinal comparison with the DMF-, TER- and CLAD-treated cohorts. Samples not passing the threshold of minimum 100 sequences (n = 126) during bootstrapping approach used to calculate diversity within one B cell subset could not be considered for comparison within the respective B cell subset. MS UT, treatment-naïve pwMS; BL, baseline; OZA, ozanimod; BL, baseline; DMF, dimethyl fumarate; TER, teriflunomide; CLAD, cladribine; N, naive B cells; DN, double negative B-cells; M, memory B-cells; P, plasmablasts.

**Suppl. Table 11: Overview on the sample number for the longitudinal comparisons of the treatment cohorts OZA, FTY, DMF, TER and NAT**

| Group | N | DN | M | P |
| --- | --- | --- | --- | --- |
| OZA | **n = 9**  OZA1  OZA2  OZA3  OZA4  OZA5  OZA6  OZA7  OZA8  OZA9 | **n = 6**  **(+3 didn’t pass n > 100 sequences)**  OZA1  OZA2  OZA3  OZA4  OZA6  OZA7 | **n = 9**  OZA1  OZA2  OZA3  OZA4  OZA5  OZA6  OZA7  OZA8  OZA9 | **n = 5**  **(+4 didn’t pass n > 100 sequences)**  OZA1  OZA2  OZA3  OZA4  OZA5 |
| FTY | **n = 3**  FTY1  FTY3  FTY4 | **n = 3**  FTY1  FTY3  FTY4 | **n = 3**  FTY1  FTY3  FTY4 | **n = 2**  **(+1 didn’t pass n > 100 sequences)**  FTY1  FTY3 |
| DMF | **n = 4**  DMF6  DMF7  DMF8  DMF9 | **n = 4**  DMF6  DMF7  DMF8  DMF9 | **n = 4**  DMF6  DMF7  DMF8  DMF9 | **n = 4**  DMF6  DMF7  DMF8  DMF9 |
| TER | **n = 5**  TER1  TER2  TER3  TER6  TER7 | **n = 5**  TER1  TER2  TER3  TER6  TER7 | **5**  TER1  TER2  TER3  TER6  TER7 | **n = 4**  **(+1 didn’t pass n > 100 sequences)**  TER1  TER2  TER6  TER7 |
| NAT | **n = 4**  NAT1  NAT2  NAT3  NAT4 | **n = 4**  NAT1  NAT2  NAT3  NAT4 | **n = 3**  **(+1 didn’t pass n > 100 sequences)**  NAT1  NAT3  NAT4 | **n = 4**  NAT1  NAT2  NAT3  NAT4 |

Samples not passing the threshold of minimum 100 sequences (n = 126) during bootstrapping approach used to calculate diversity within one B cell subset could not be considered for comparison within the respective B cell subset. N, naive B cells; DN, double negative B-cells, M, memory B-cells; P, plasmablasts; OZA, ozanimod; FTY, fingolimod; NAT, natalizumab; DMF, dimethyl fumarate; TER, teriflunomide; CLAD, cladribine.

**Supplementary Figures:**

**
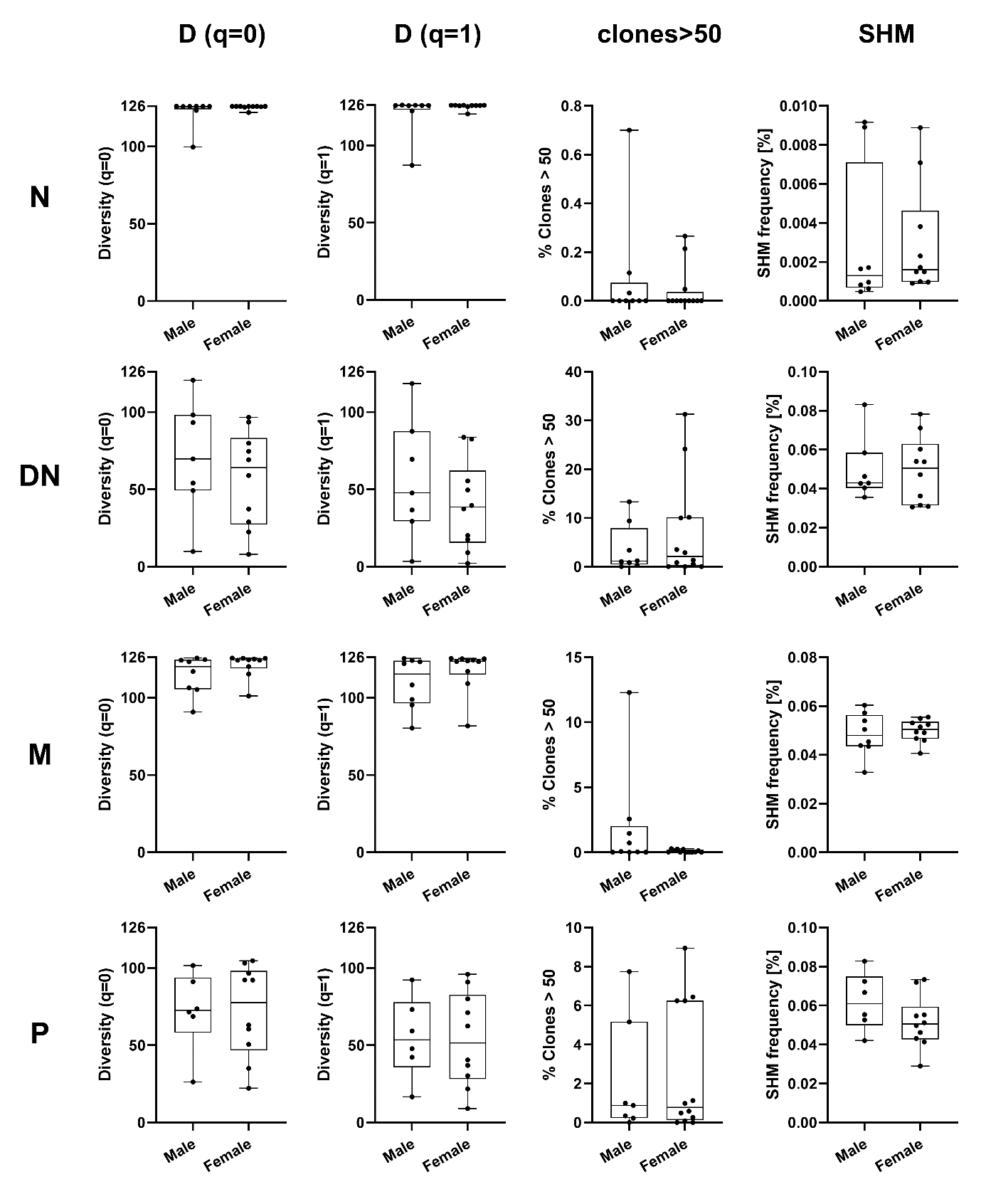
**

**Suppl. Figure 1:** Clonal analysis for the cross-sectional comparison between male and female pwMS from the treatment-naïve (MS UT) MS cohort. Each column represents one metric including clonal diversity expressed as Hill numbers for q = 0 and q = 1, accounting for the total number of clones and weighted by clone size, respectively, percentage of clones comprising more than 50 unique sequences per clone, and, somatic hypermutation frequency. Comparison data are shown per row for one B cell subset (N, DN, M, P). For statistical analyses the Mann Whitney test was performed. N, naive B cells; DN, double negative B cells; M, memory B cells; P, plasmablasts; MS UT, treatment-naïve pwMS; D, diversity; SHM, somatic hypermutation.

**
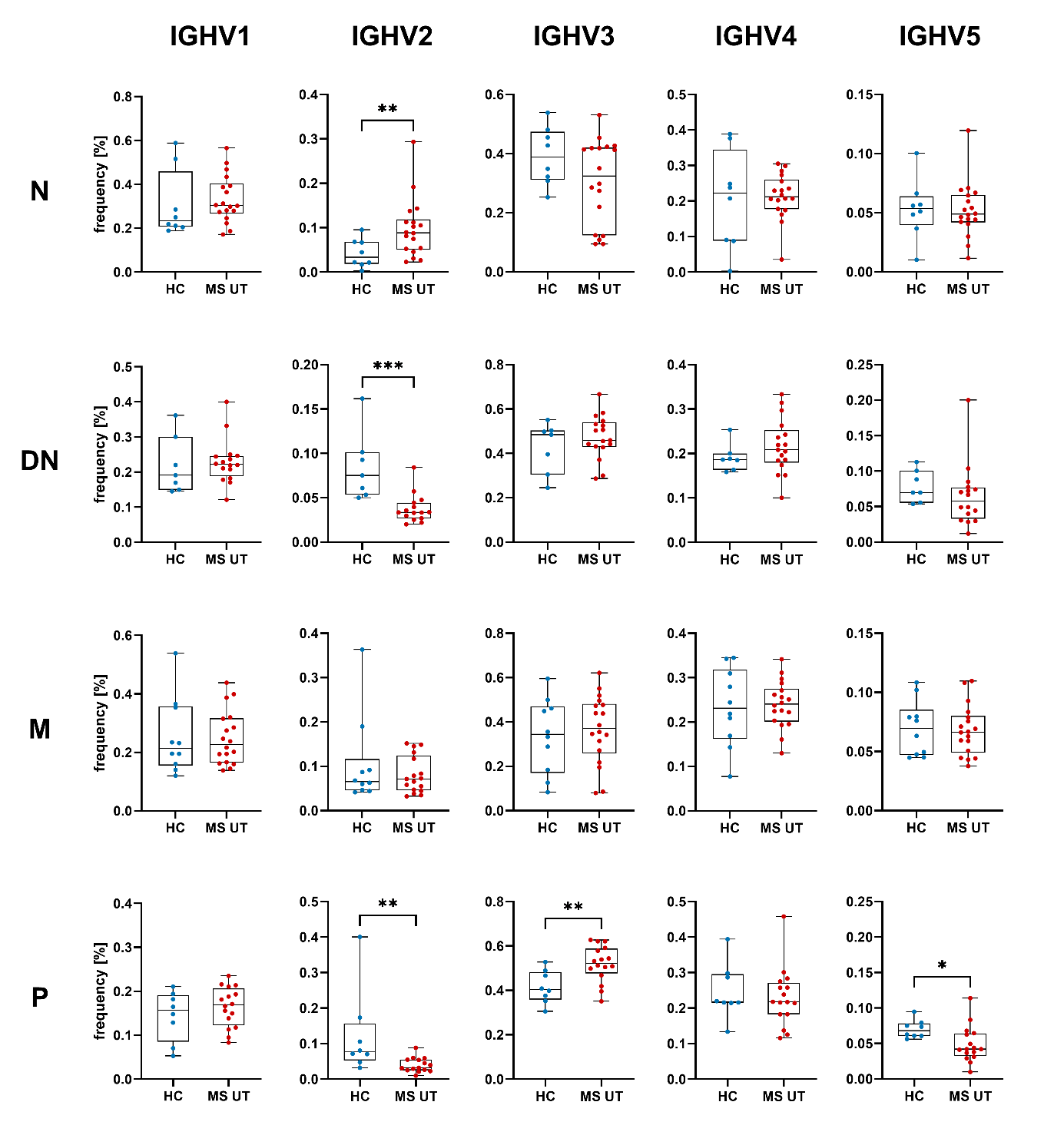
Suppl. Figure 2:** Gene usage compared cross-sectionally between healthy controls (HC) and treatment-naïve MS patients (MS UT). Comparisons are shown for the VH family genes IGHV1-IGHV5 for each B cell subset (N, DN, M, P). For statistical analyses the Mann Whitney test was performed (^#^p<0.1; *p<0.05; **p<0.01).

**
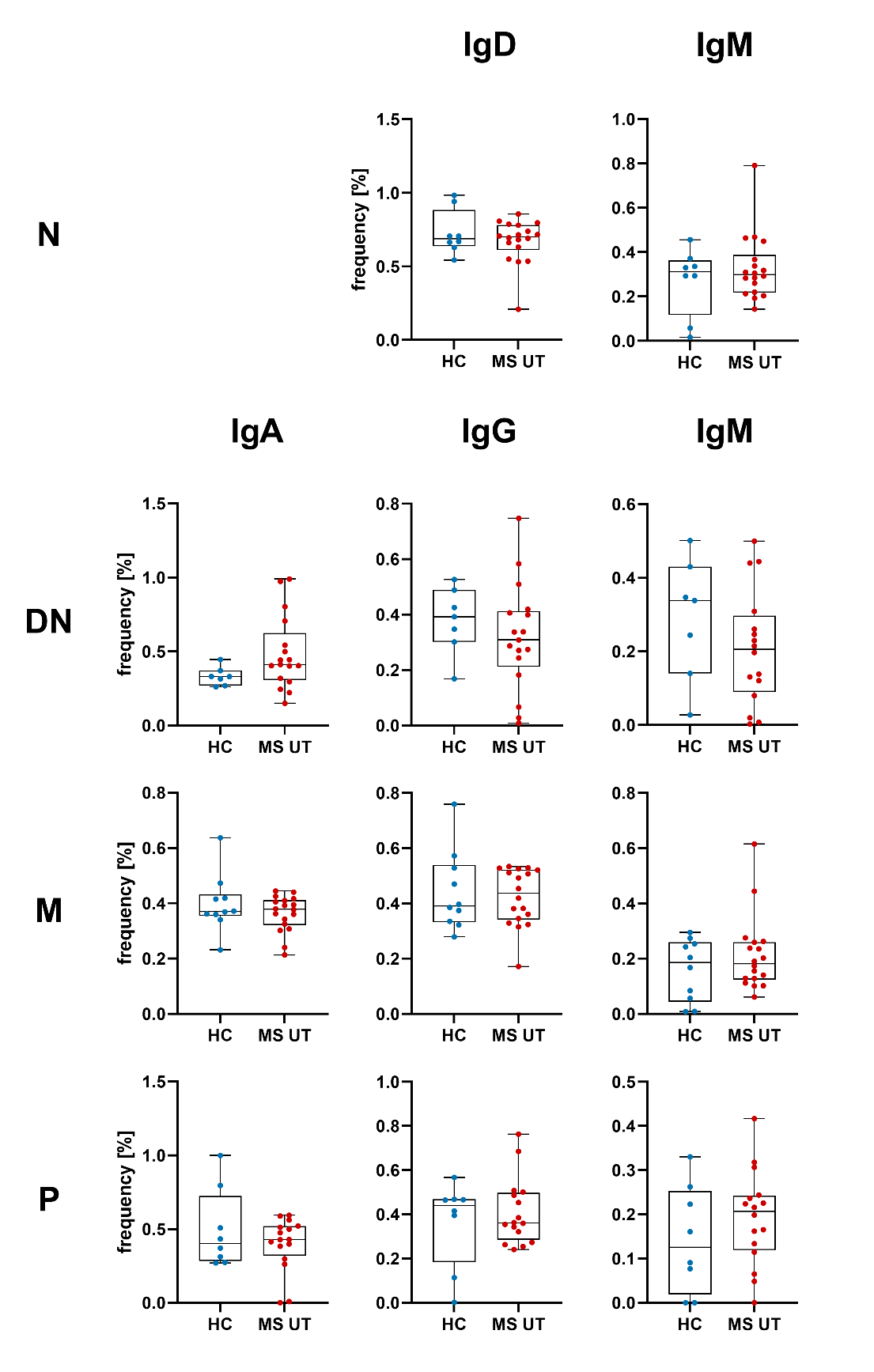
Suppl. Figure 3:** Isotype frequency compared cross-sectionally between healthy controls (HC) and treatment-naïve MS patients (MS UT). Comparisons are shown for the isotypes IgD and IgM for the naive B cells and for the isotypes IgA, IgG and IgM for the remaining B cell subsets (DN, M, P). For statistical analyses the Mann Whitney test was performed.

**
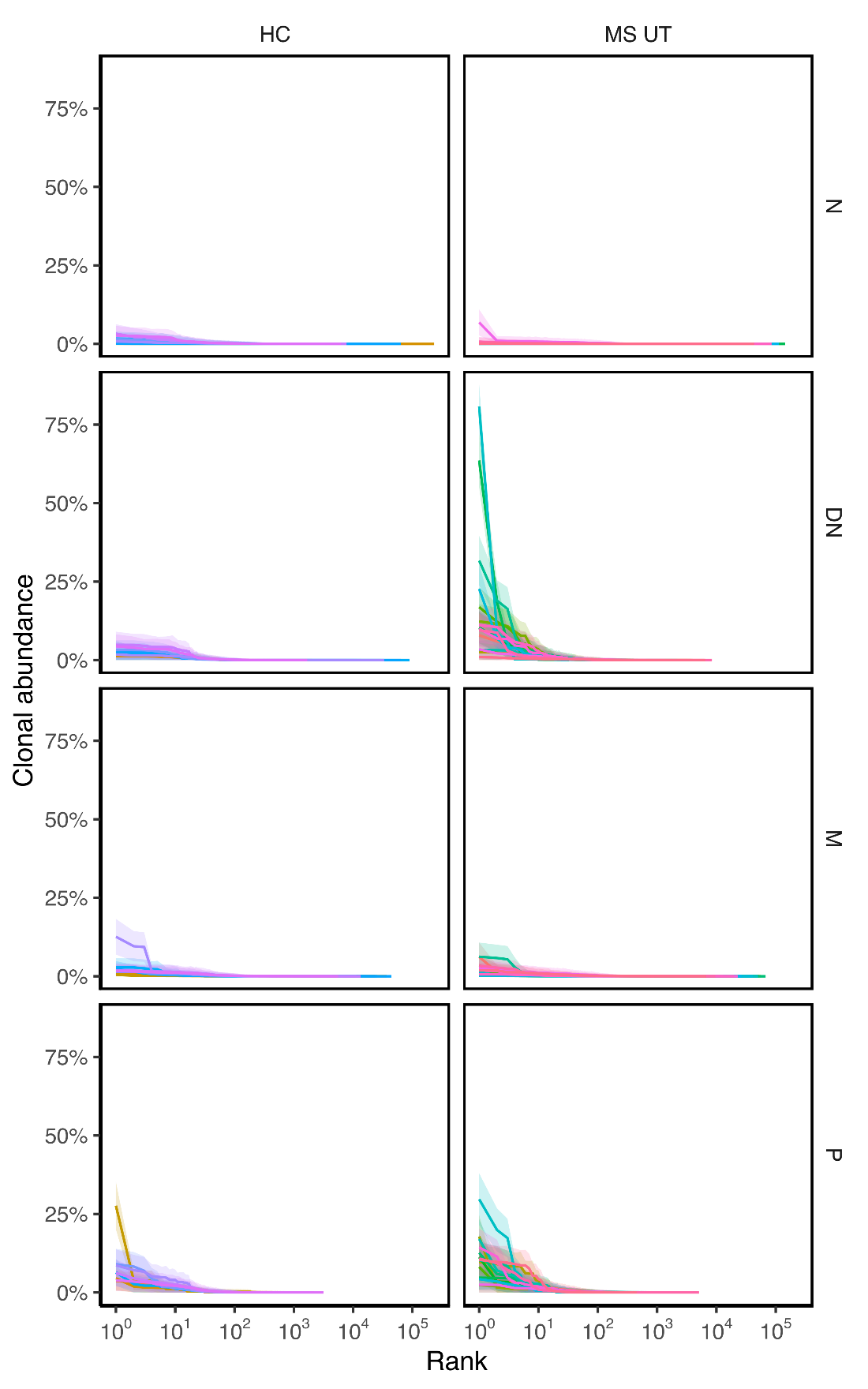
Suppl. Figure 4:** Clonal abundance curves of B cell receptor sequences compared cross-sectionally between healthy controls (HC) and treatment-naïve MS patients (MS UT). Clonal abundance curves are shown for each subject and B cell subset (N, DN, M, P).

**Suppl. Figure 5:** Diversity curves compared cross-sectionally between healthy controls (HC) and treatment-naïve MS patients (MS UT).
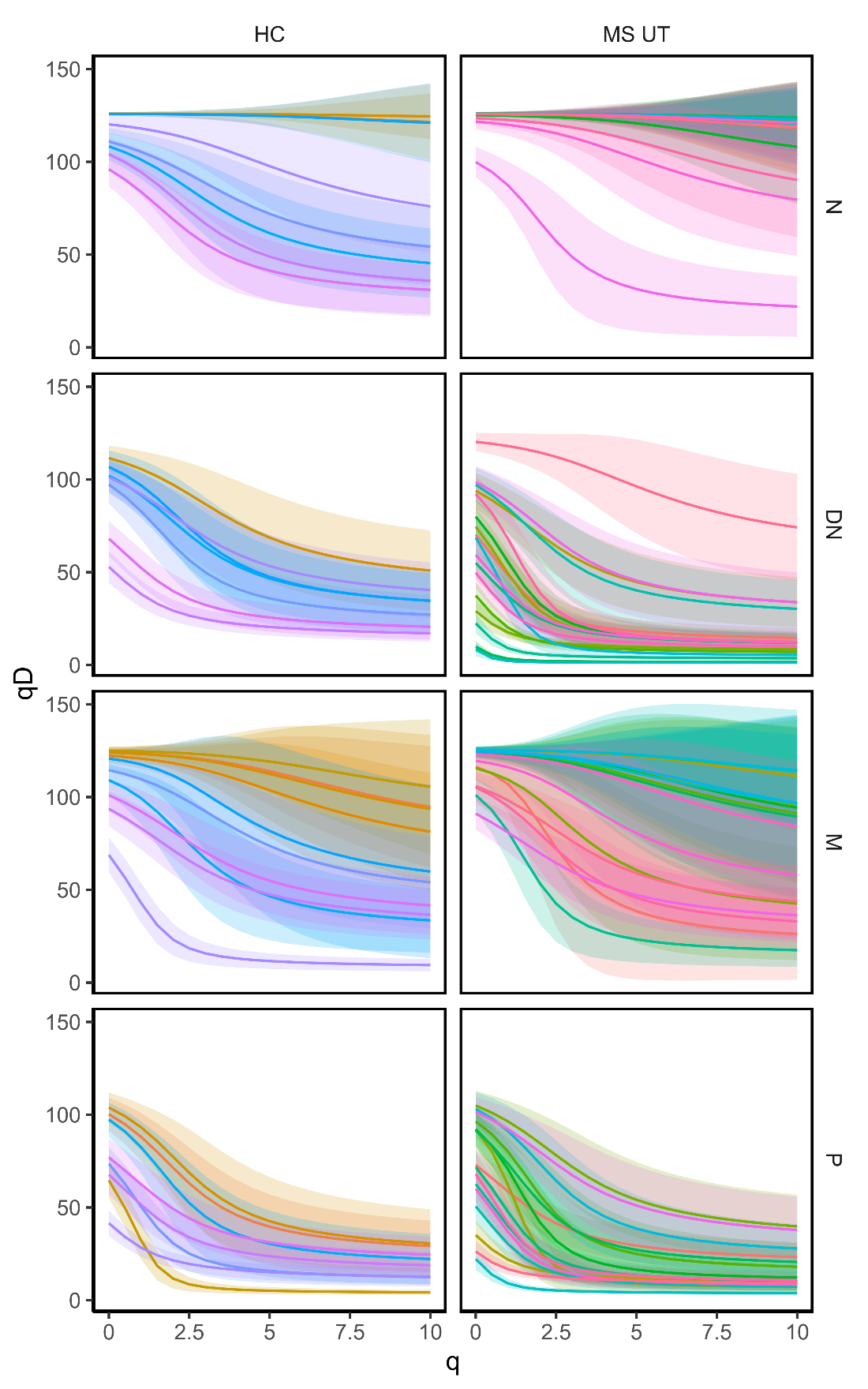
 Diversity values were obtained on a bootstrap sample of n = 126 sequences with 200 repetitions. Diversity curves were plotted as function of the Hill number q for each subject and B cell subset (N, DN, M, P).

**
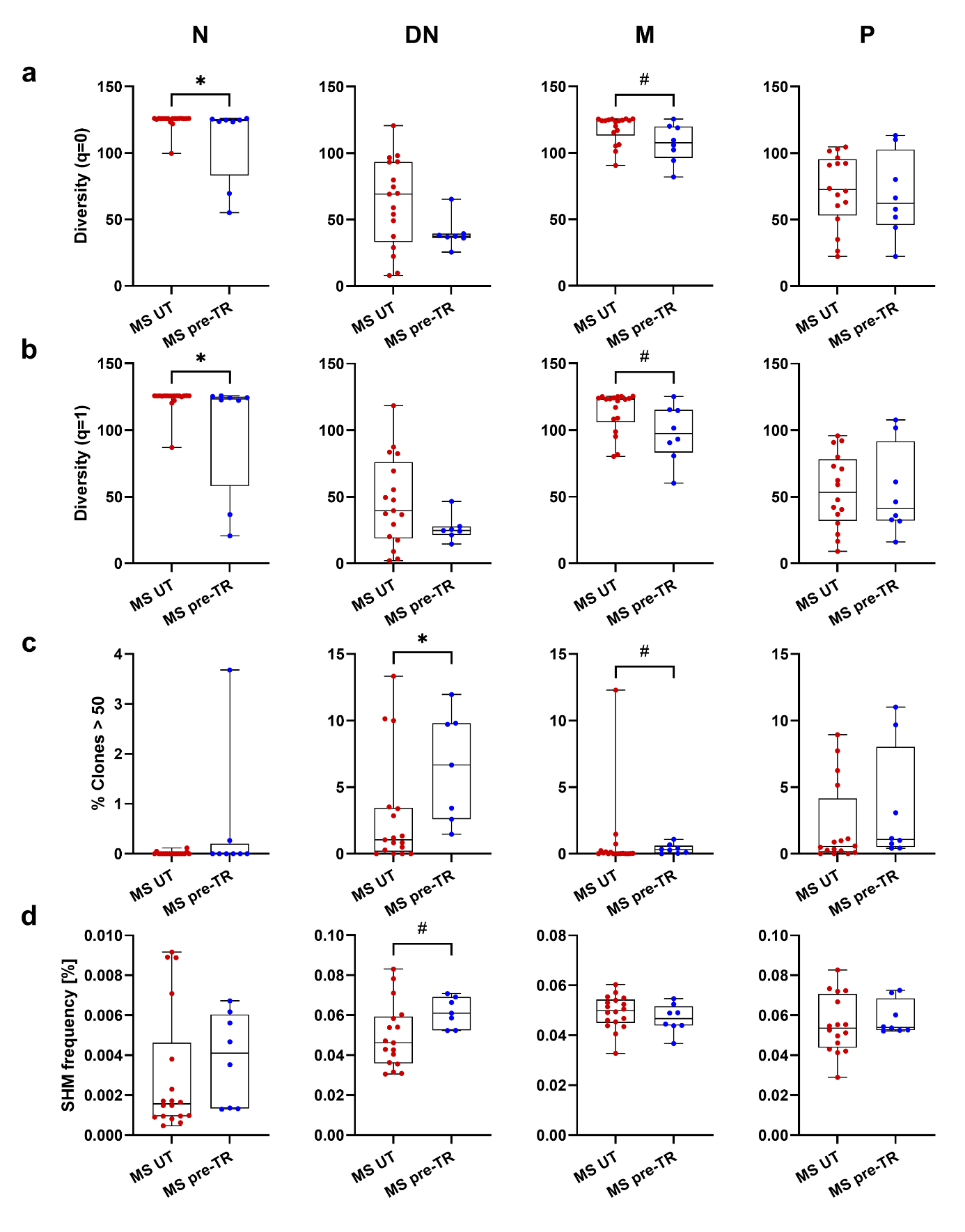
Suppl. Figure 6:** Clonal analysis for the cross-sectional comparison between treatment-naïve MS patients (MS UT) and pretreated MS patients (MS pre-TR). **(a)** Clonal diversity expressed as Hill numbers for q = 0, accounting for the total number of clones and **(b)** clonal diversity expressed as Hill numbers for q = 1, accounting for the total number of clones weighted by clone size. **(c)** Percentage of large clones comprising more than 50 unique sequences per clone. Percentage values should be treated with caution as they were not obtained on a bootstrapping approach and thus do not account for sequencing depth to avoid a down sampling of the sequence count. **(d)** Somatic hypermutation frequency (SHM). Per metric, data is shown for each subject per comparison group and B cell subset (N, DN, M, P). For statistical analyses the Mann Whitney test was performed (^#^p<0.1).

**
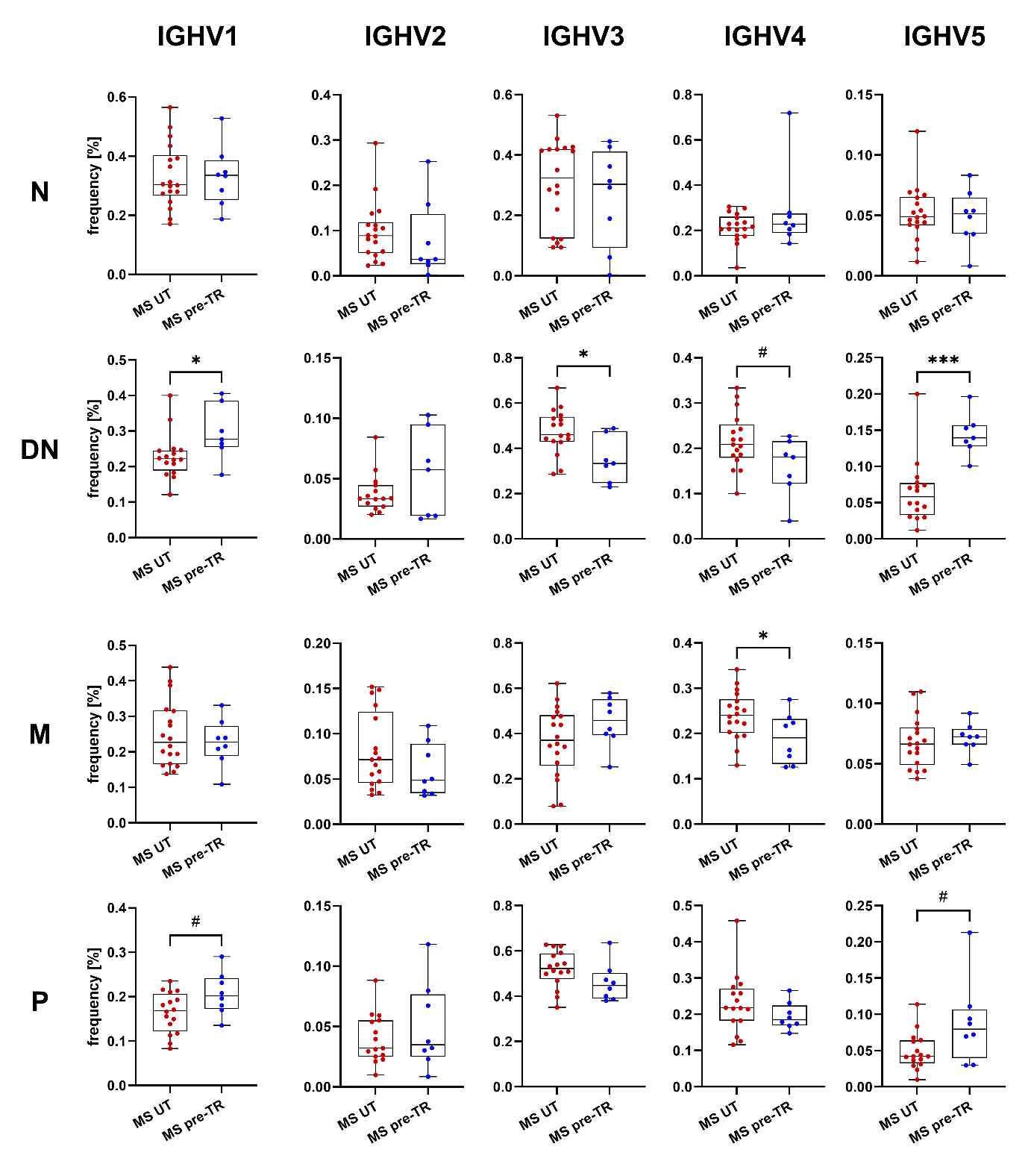
Suppl. Figure 7:** Gene usage compared cross-sectionally between treatment-naïve MS patients (MS UT) and pretreated MS patients (MS pre-TR). Comparisons are shown for the VH family genes IGHV1-IGHV5 for each B cell subset (N, DN, M, P). For statistical analyses the Mann Whitney test was performed (^#^p<0.1; *p<0.05; **p<0.01).

**
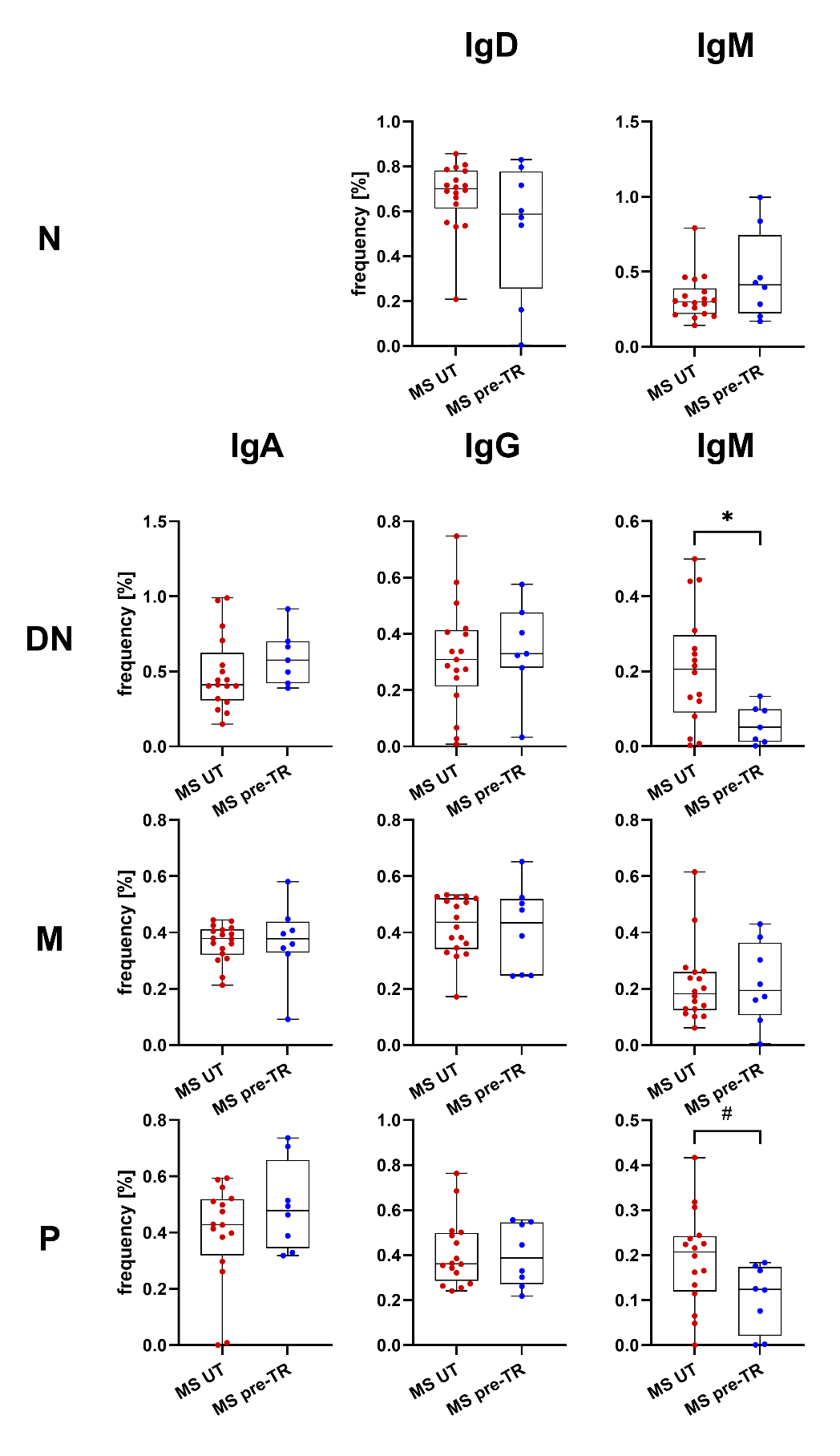
Suppl. Figure 8:** Isotype frequency compared cross-sectionally between treatment-naïve MS patients (MS UT) and pretreated MS patients (MS pre-TR). Comparisons are shown for the isotypes IgD and IgM for the naive B cells and for the isotypes IgA, IgG and IgM for the remaining B cell subsets (DN, M, P). For statistical analyses the Mann Whitney test was performed (#p<0.1).

**
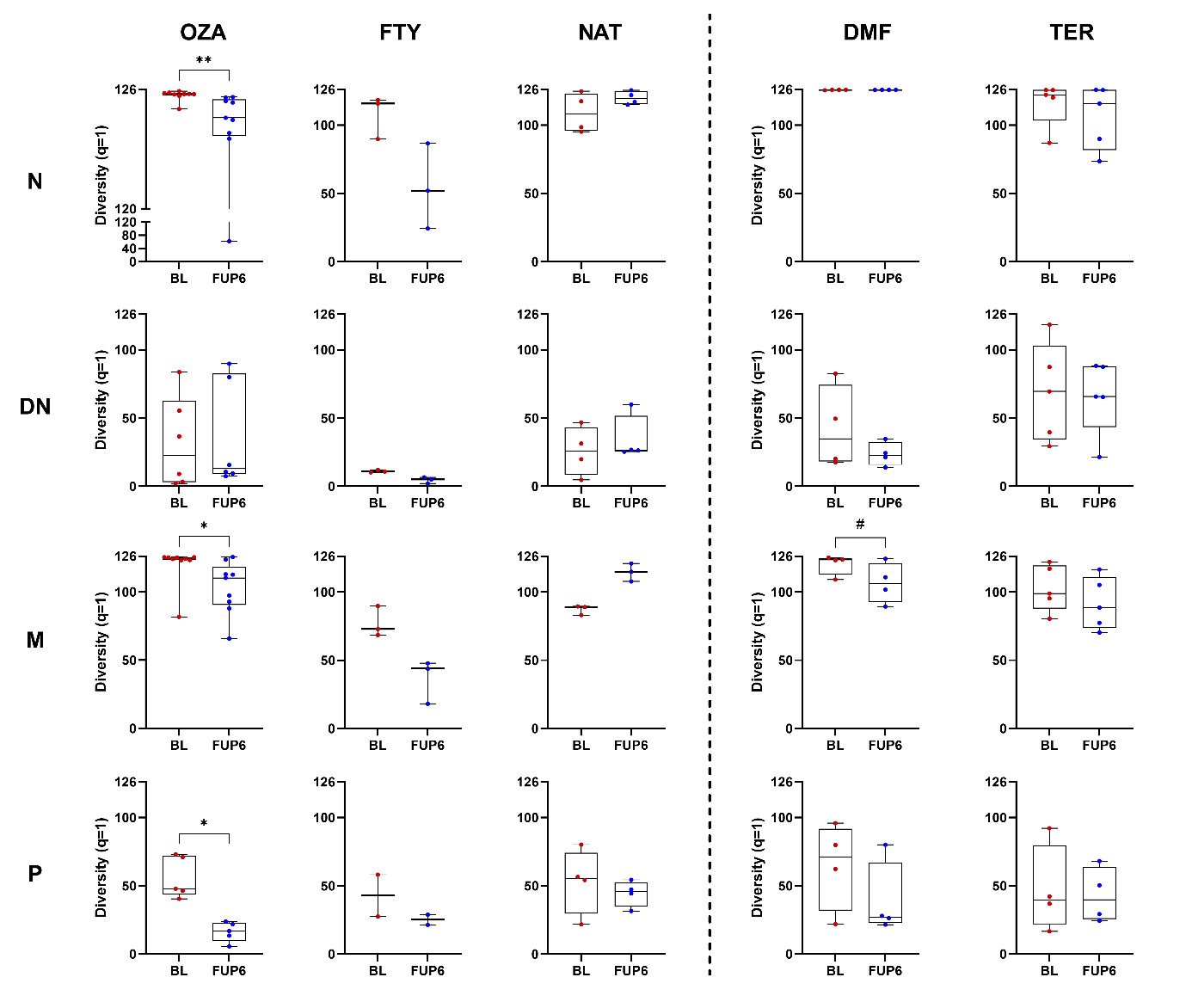
Suppl. Figure 9:** Clonal diversity expressed as Hill numbers for q = 1, accounting for the total number of clones weighted by clone size. The clonal diversity is shown for each subject per treatment group (OZA, FTY, NAT, DMF, TER) and B cell subset (N, DN, M, P) and compared longitudinally between the time points (BL, FUP6). For statistical analyses the Wilcoxon Rank-Sum paired test was performed (^#^p<0.1; *p<0.05; **p<0.01).


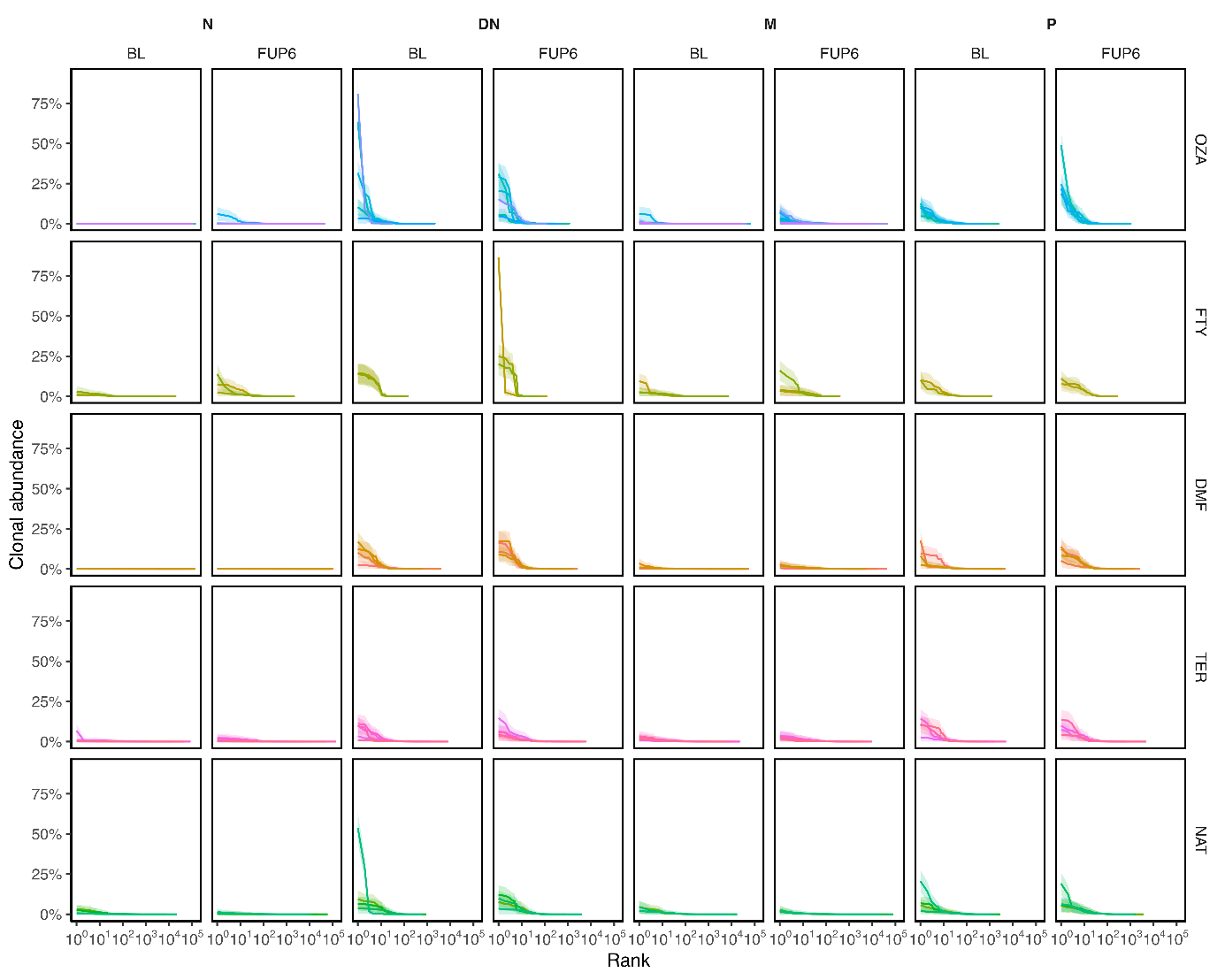
**Suppl. Figure 10:** Clonal abundance curves of B cell receptor sequences shown for each longitudinal treatment group (OZA, FTY, DMF, TER, NAT). Clonal abundance curves are shown for each subject and B cell subset (N, DN, M, P).

**Suppl. Figure 11:** Diversity curves shown for each longitudinal treatment group (OZA, FTY, DMF, TER, NAT). Diversity values were obtained on a bootstrap sample of n = 126 sequences with 200 repetitions. Diversity curves were plotted as function of the Hill number q for each subject and B cell subset (N, DN, M, P).
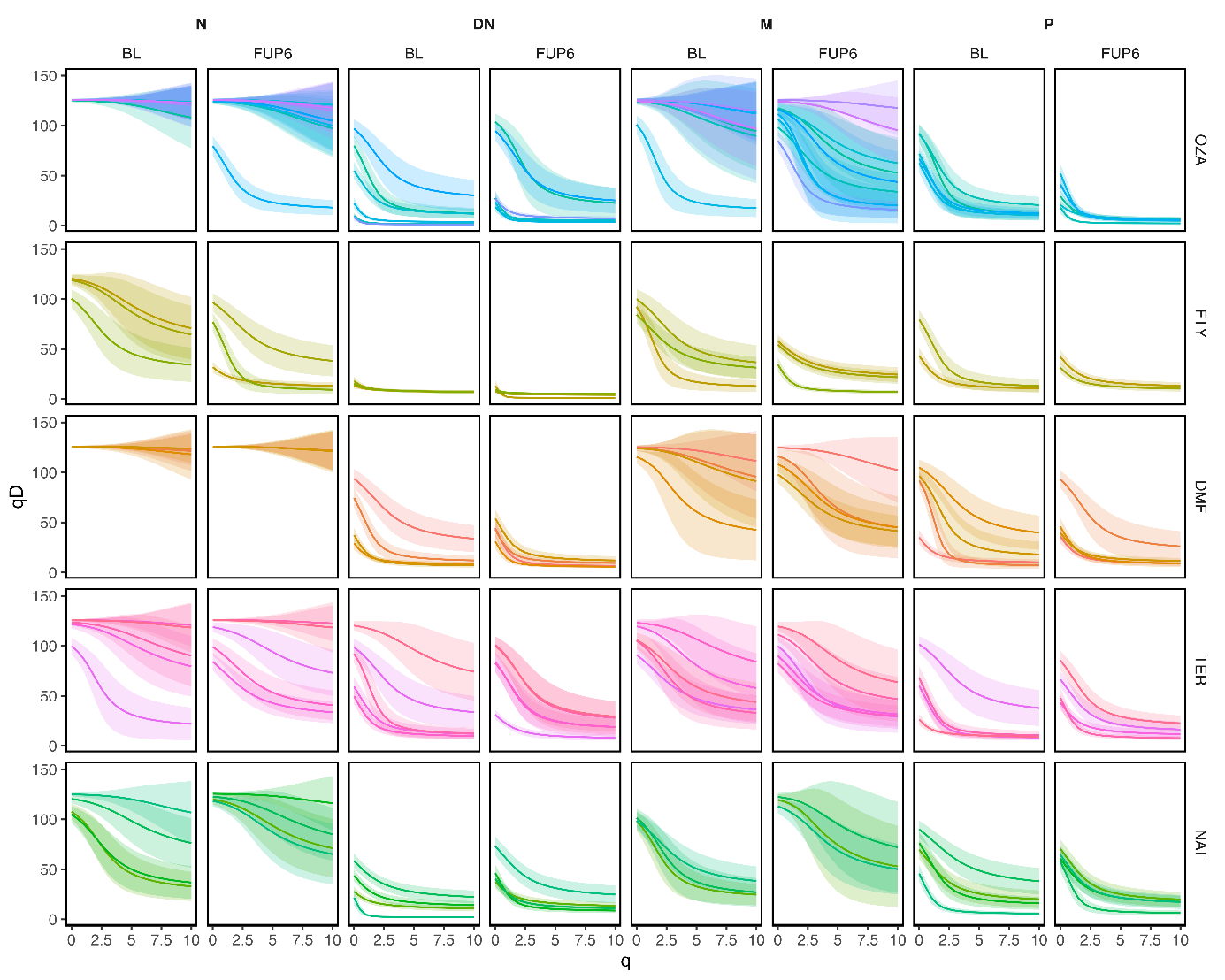


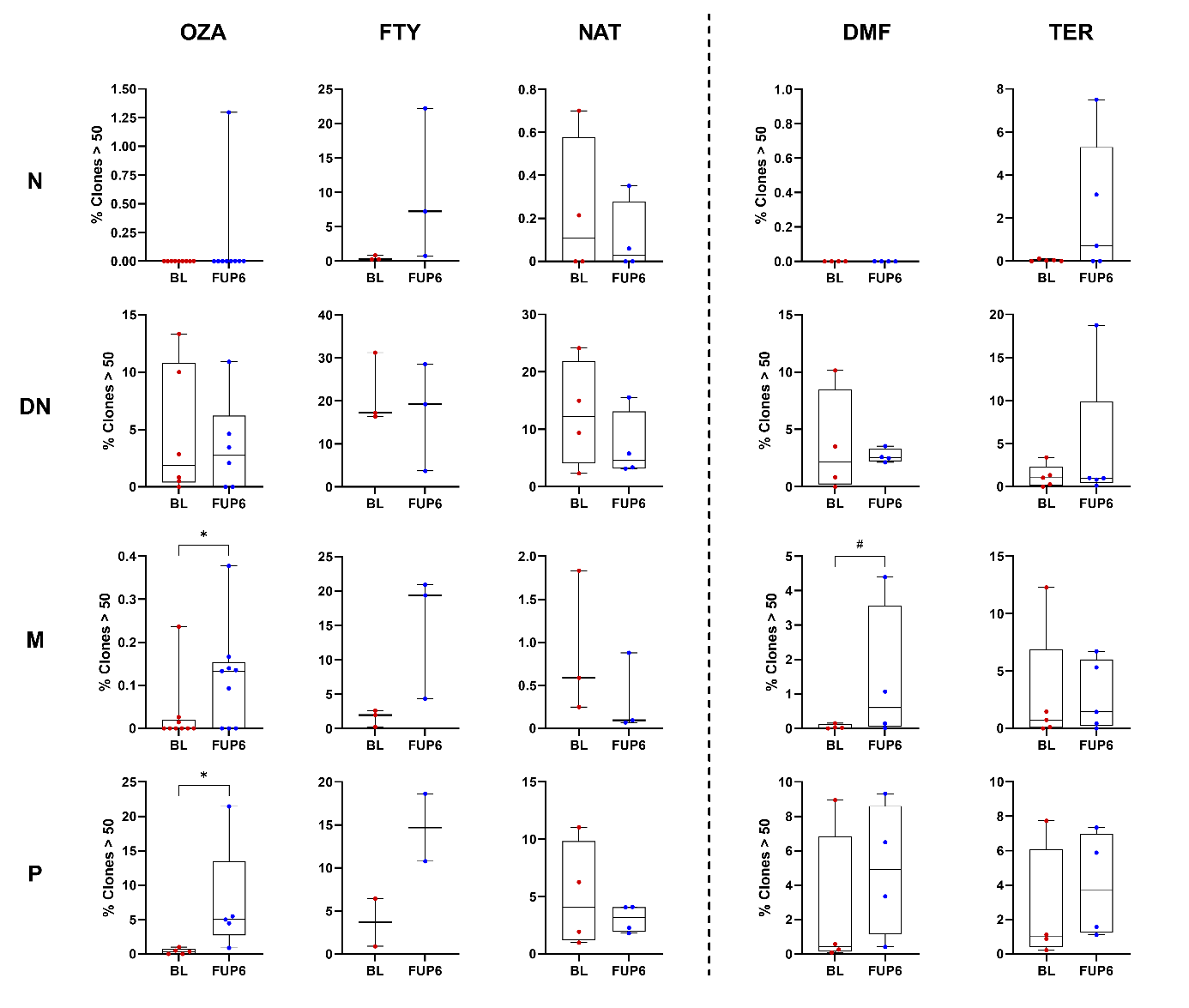
**Suppl. Figure 12:** Percentage of large clones comprising more than 50 unique sequences per clone are shown for each subject per treatment group (OZA, FTY, NAT, DMF, TER) and B cell subset (N, DN, M, P) and compared longitudinally between the time points (BL, FUP6). For statistical analyses the Wilcoxon Rank-Sum paired test was performed (^#^p<0.1; *p<0.05).

**Suppl. Figure 13:** Longitudinal comparison of somatic hypermutation (SHM) frequency between the two time points (BL, FUP6) when following a certain treatment (OZA, FTY, NAT, DMF, TER) shown for each subject and cell subset (N, DN, M, P). For statistical analyses the Wilcoxon Rank-Sum paired test was performed.
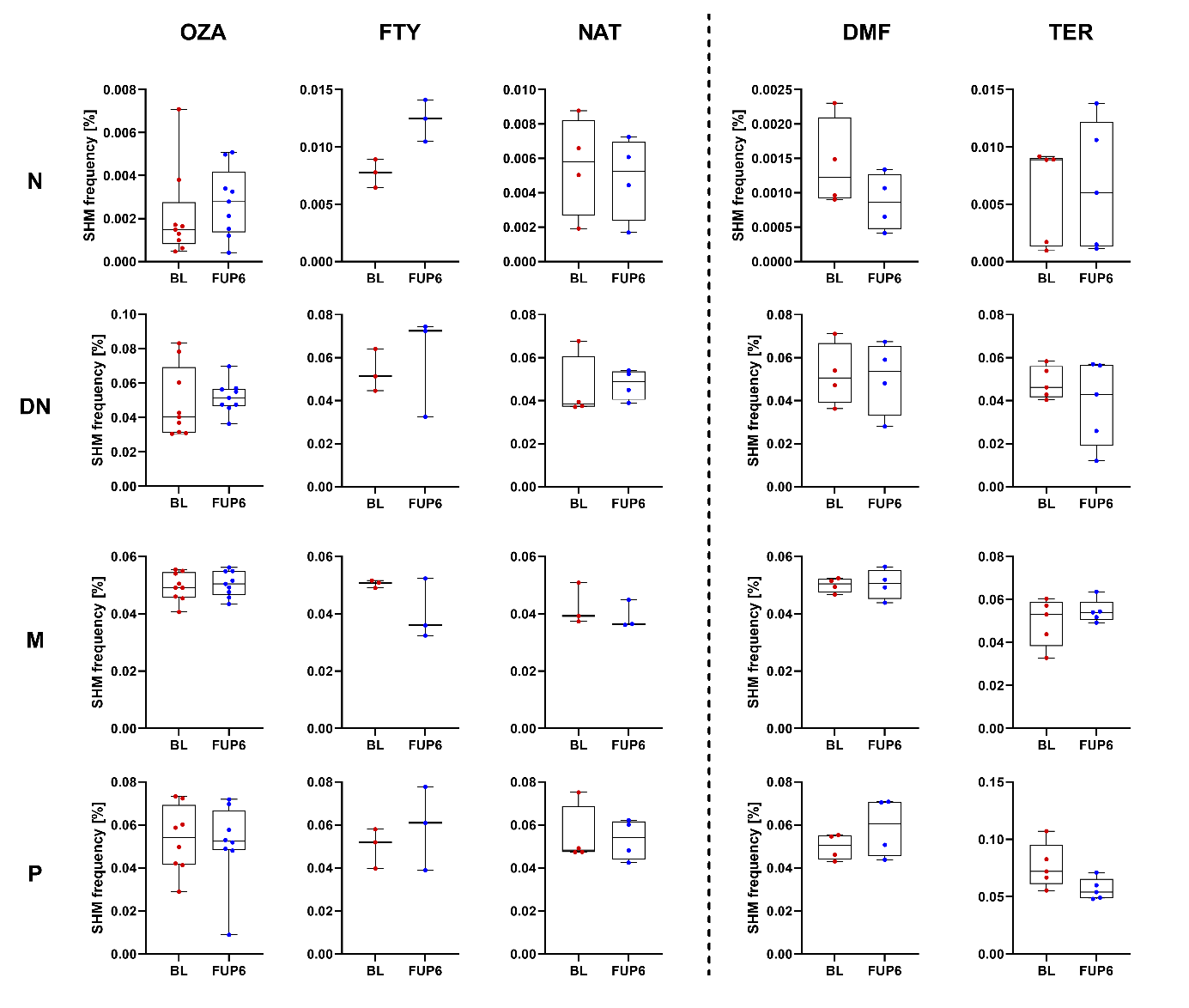


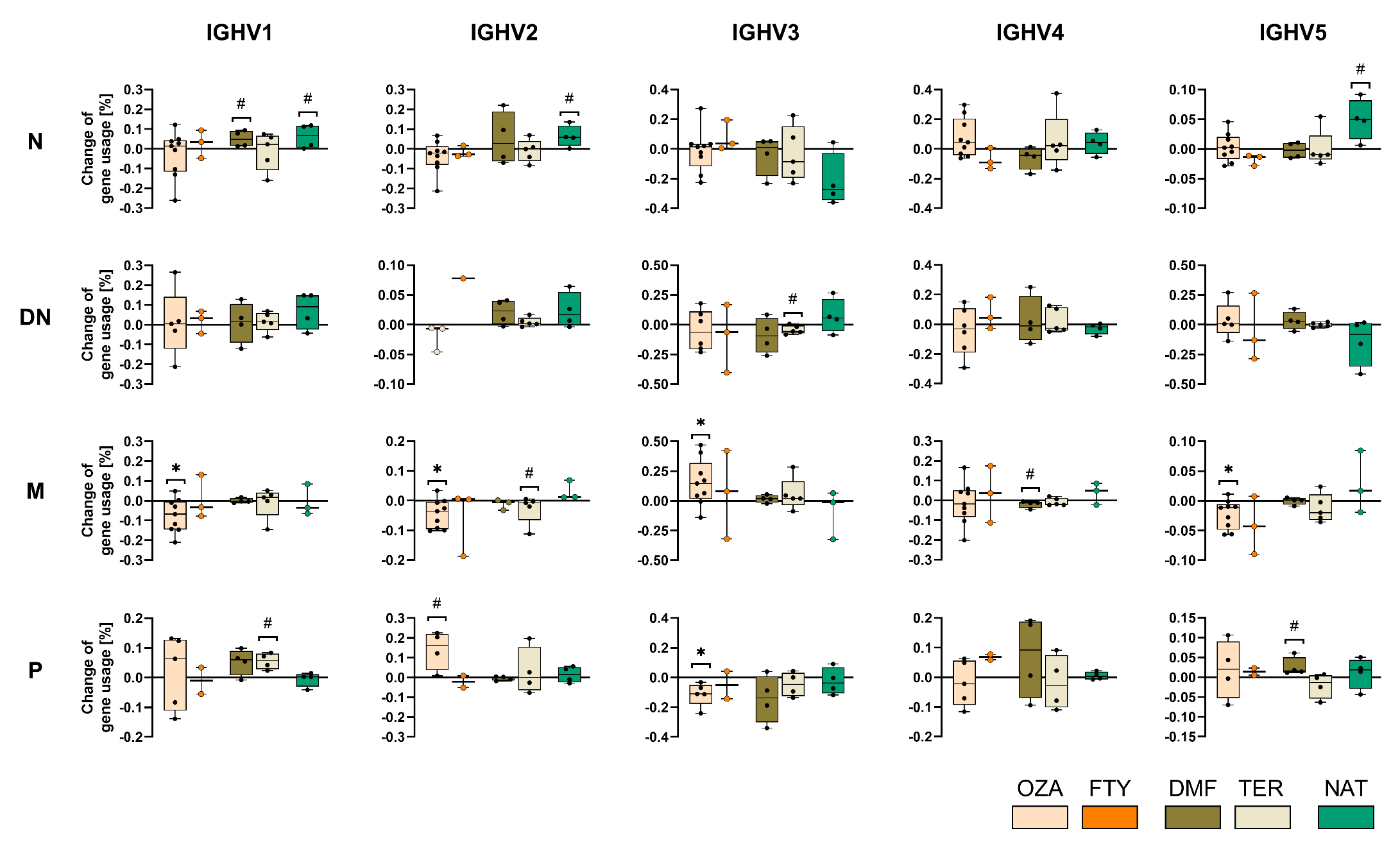
**Suppl. Figure 14:** The change in gene usage in % after six months of a certain treatment (OZA, FTY, NAT, DMF, TER) is shown for the VH family genes IGHV1-IGHV5 for each B cell subset (N, DN, M, P). For statistical analyses the Wilcoxon Rank-Sum paired test was performed within one treatment group for each gene, cell subset and between both time points (BL vs. FUP6), (^#^p<0.1; *p<0.05).


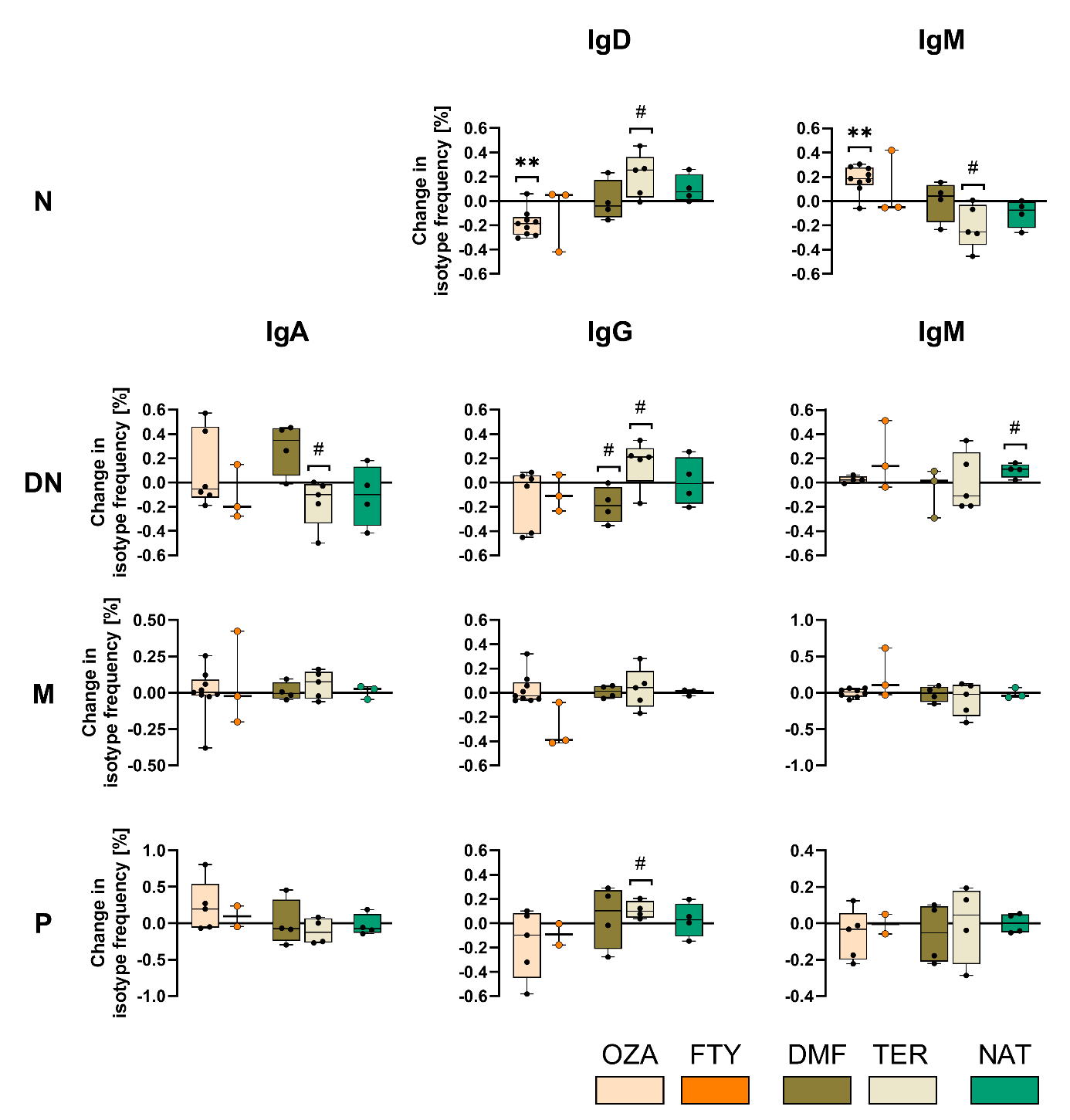
**Suppl. Figure 15:** The change in isotype frequency in % after six months of a certain treatment (OZA, FTY, NAT, DMF, TER) is shown for the isotypes IgD and IgM for the naive B cells and for the isotypes IgA, IgG and IgM for the remaining B cell subsets (DN, M, P). For statistical analyses the Wilcoxon Rank-Sum paired test was performed within one treatment group for each isotype, cell subset and between both time points (BL vs. FUP6), (^#^p<0.1; **p<0.01).

**
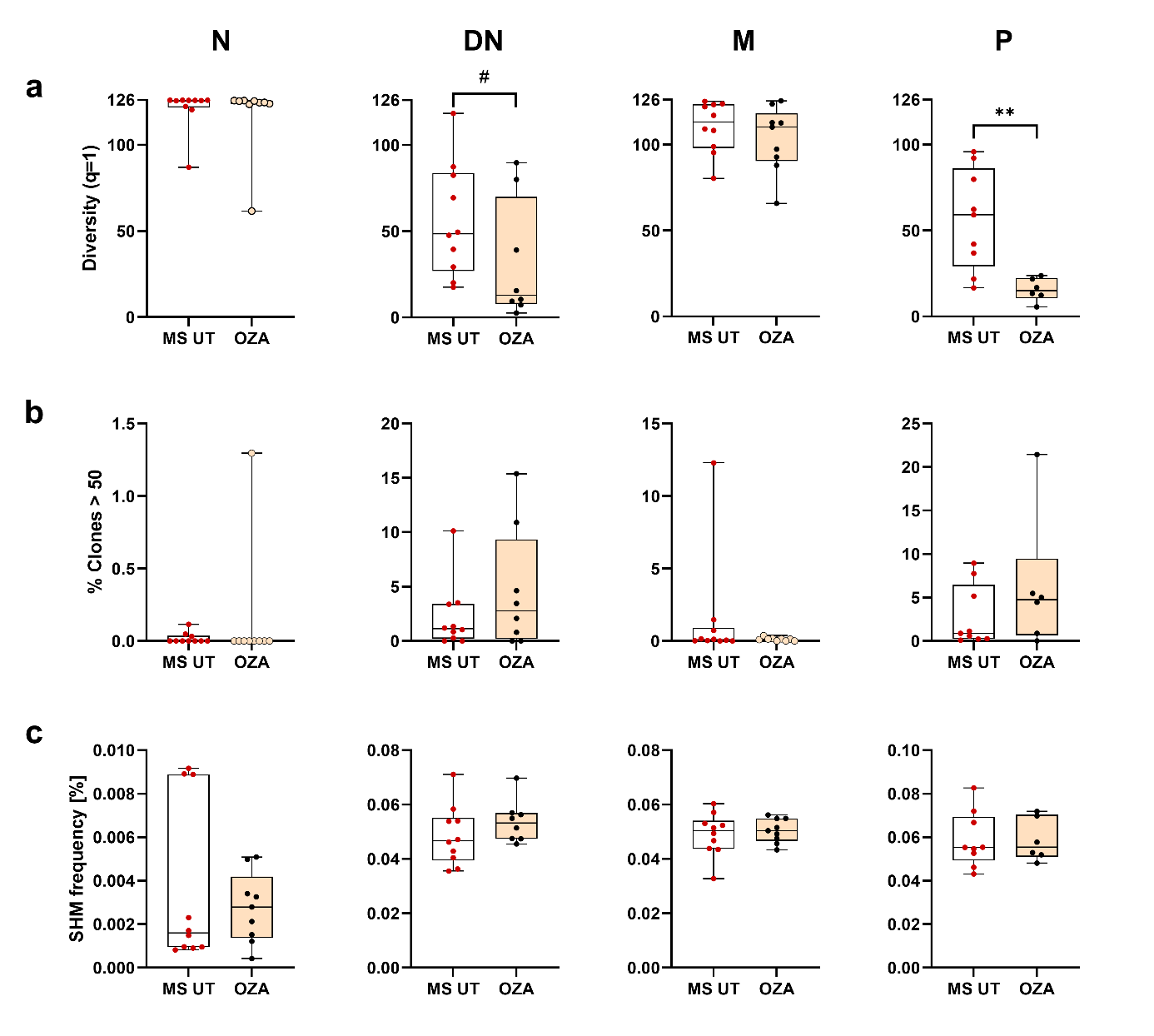
Suppl. Figure 16:** Clonal analysis for the cross-sectional comparison between OZA treated patients (following six months of treatment) and treatment-naïve MS patients (MS UT). **(a)** Clonal diversity expressed as Hill numbers for q=1, accounting for the total number of clones weighted by clone size. **(b)** Percentage of large clones comprising more than 50 unique sequences per clone. **(c)** Somatic hypermutation frequency (SHM). Per metric, data is shown for each subject per comparison group and B cell subset (N, DN, M, P). For statistical analyses the Mann-Whitney test was performed (^#^p<0.1; *p<0.05).

**
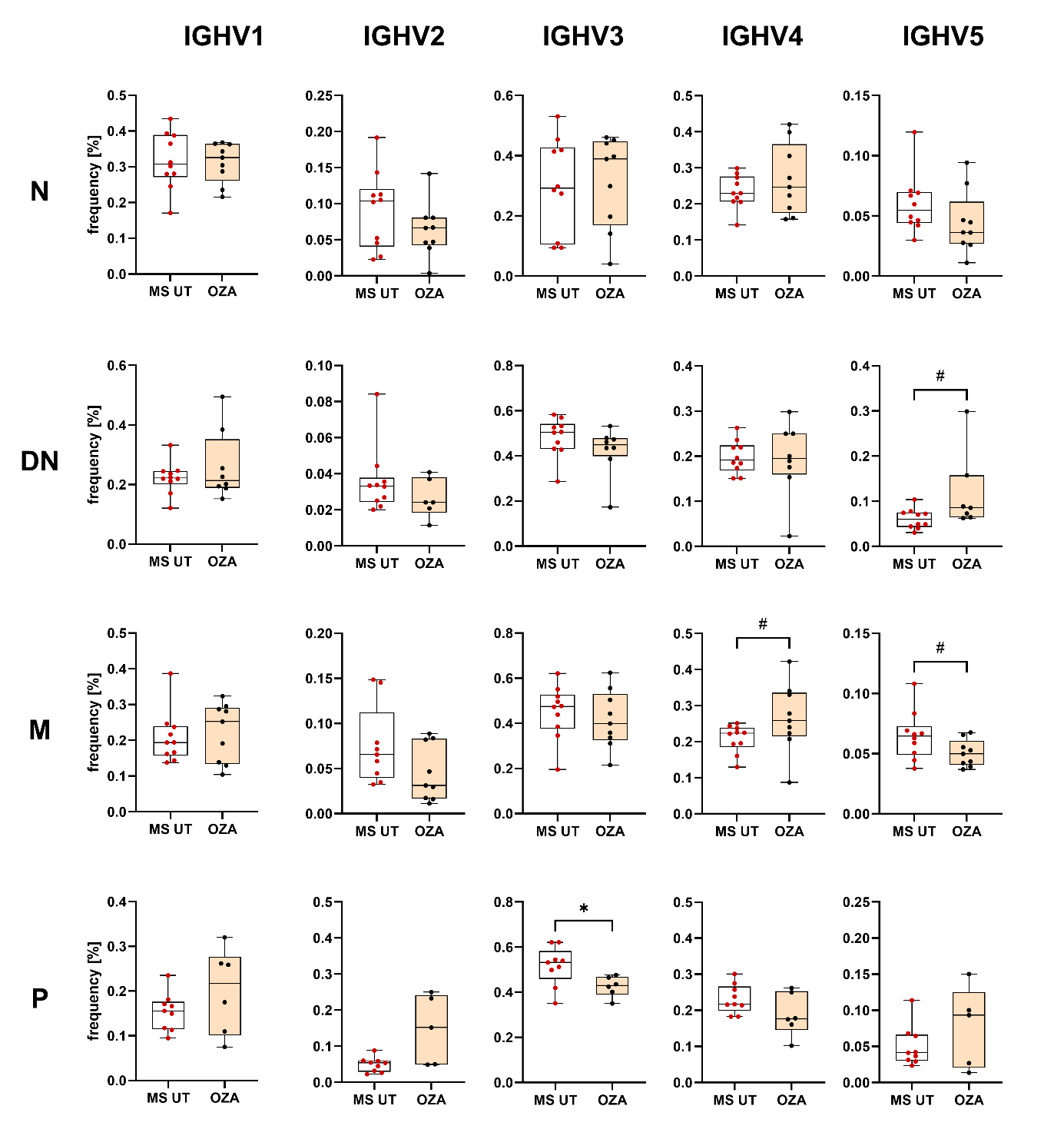
Suppl. Figure 17:** Gene usage compared cross-sectionally between OZA treated patients (following six months of treatment) and treatment-naïve MS patients (MS UT). Comparisons are shown for the VH family genes IGHV1-IGHV5 for each B cell subset (N, DN, M, P). For statistical analyses the Mann Whitney test was performed (^#^p<0.1; *p<0.05).

**
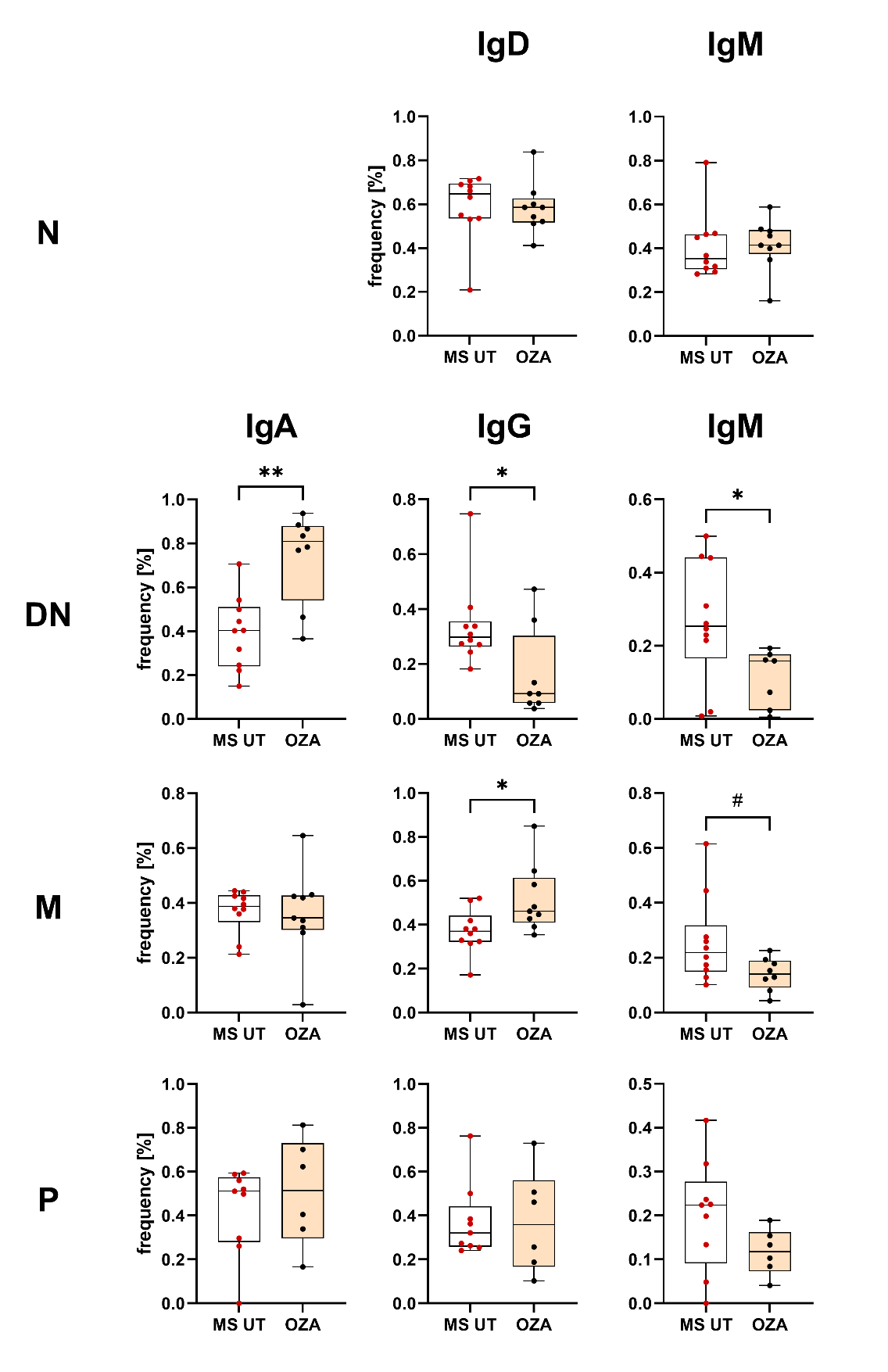
Suppl. Figure 18:** Isotype frequency compared cross-sectionally between OZA treated patients (following six months of treatment) and treatment-naïve MS patients (MS UT). Comparisons are shown for the isotypes IgD and IgM for the naive B cells and for the isotypes IgA, IgG and IgM for the remaining B cell subsets (DN, M, P). For statistical analyses the Mann Whitney test was performed (^#^p<0.1; *p<0.05; **p<0.01).

**
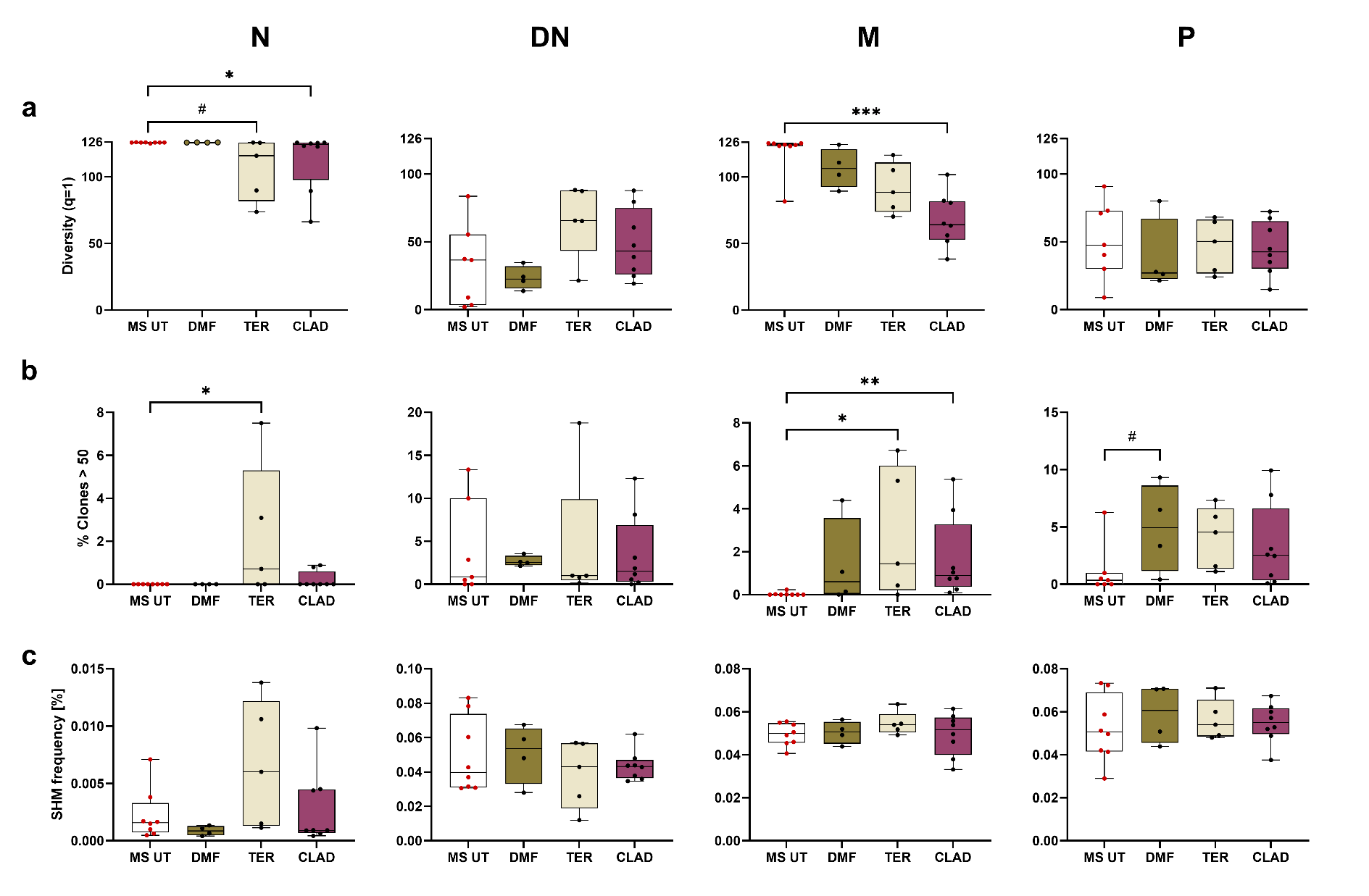
Suppl. Figure 19:** Clonal analysis for the cross-sectional comparison between DMF, TER and CLAD treated patients (following six months of treatment) and treatment-naïve patients (MS UT) from the OZA cohort (baseline). **(a)** Clonal diversity expressed as Hill numbers for q=1, accounting for the total number of clones and the clonal size. **(b)** Percentage of large clones comprising more than 50 unique sequences per clone. **(c)** Somatic hypermutation frequency (SHM). Per metric, data is shown for each subject per comparison group and B cell subset (N, DN, M, P). For statistical analyses the Kruskal-Wallis test was performed (^#^p<0.1; *p<0.05; ***p<0.001).

**
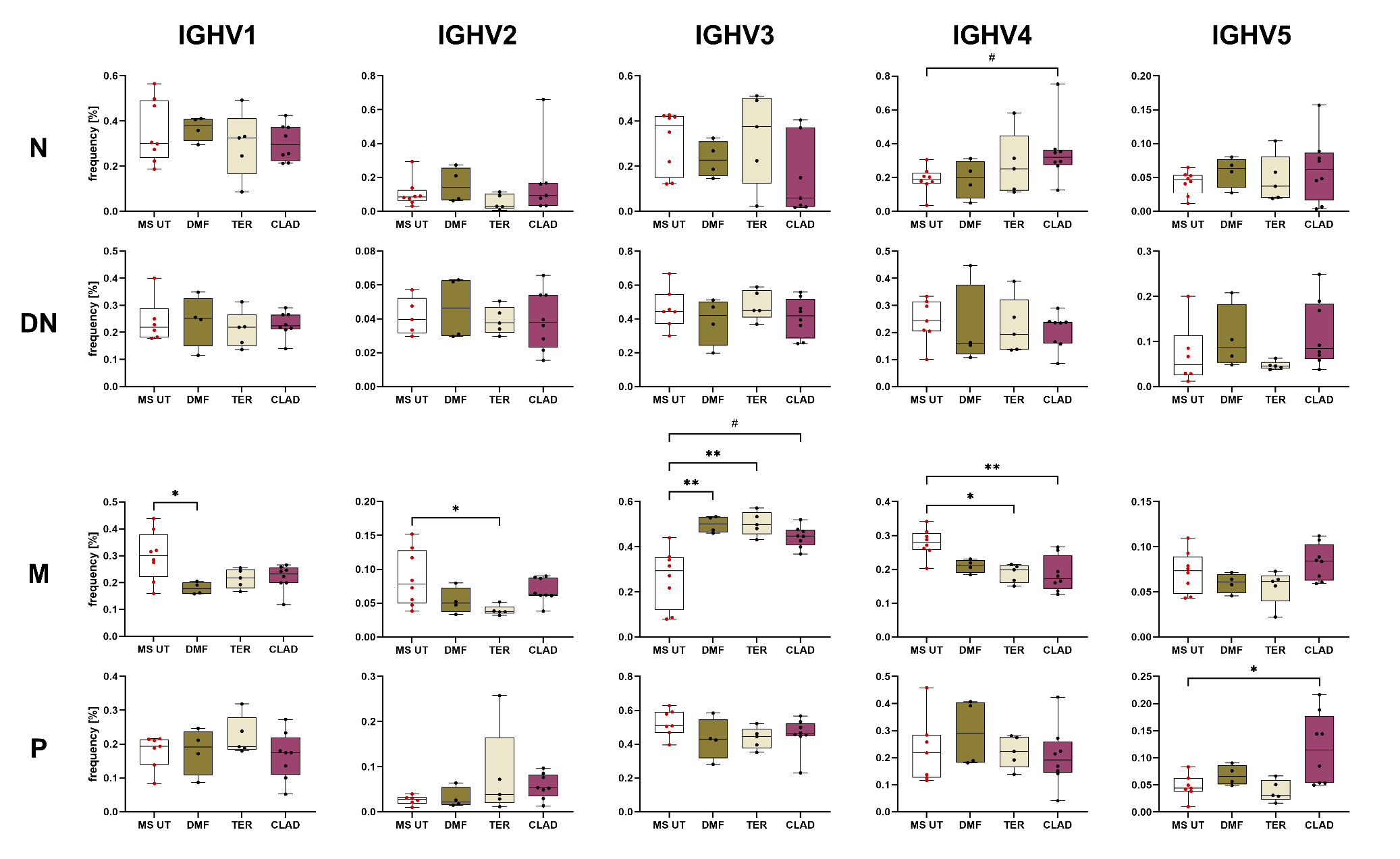
Suppl. Figure 20:** Gene usage compared cross-sectionally between DMF, TER and CLAD treated patients (following 6 months of treatment) and treatment-naïve patients (MS UT) from the OZA cohort (baseline). Comparisons are shown for the VH family genes IGHV1-IGHV5 for each B cell subset (N, DN, M, P). For statistical analyses the Kruskal-Wallis test was performed (^#^p<0.1; *p<0.05; **p<0.01).

**
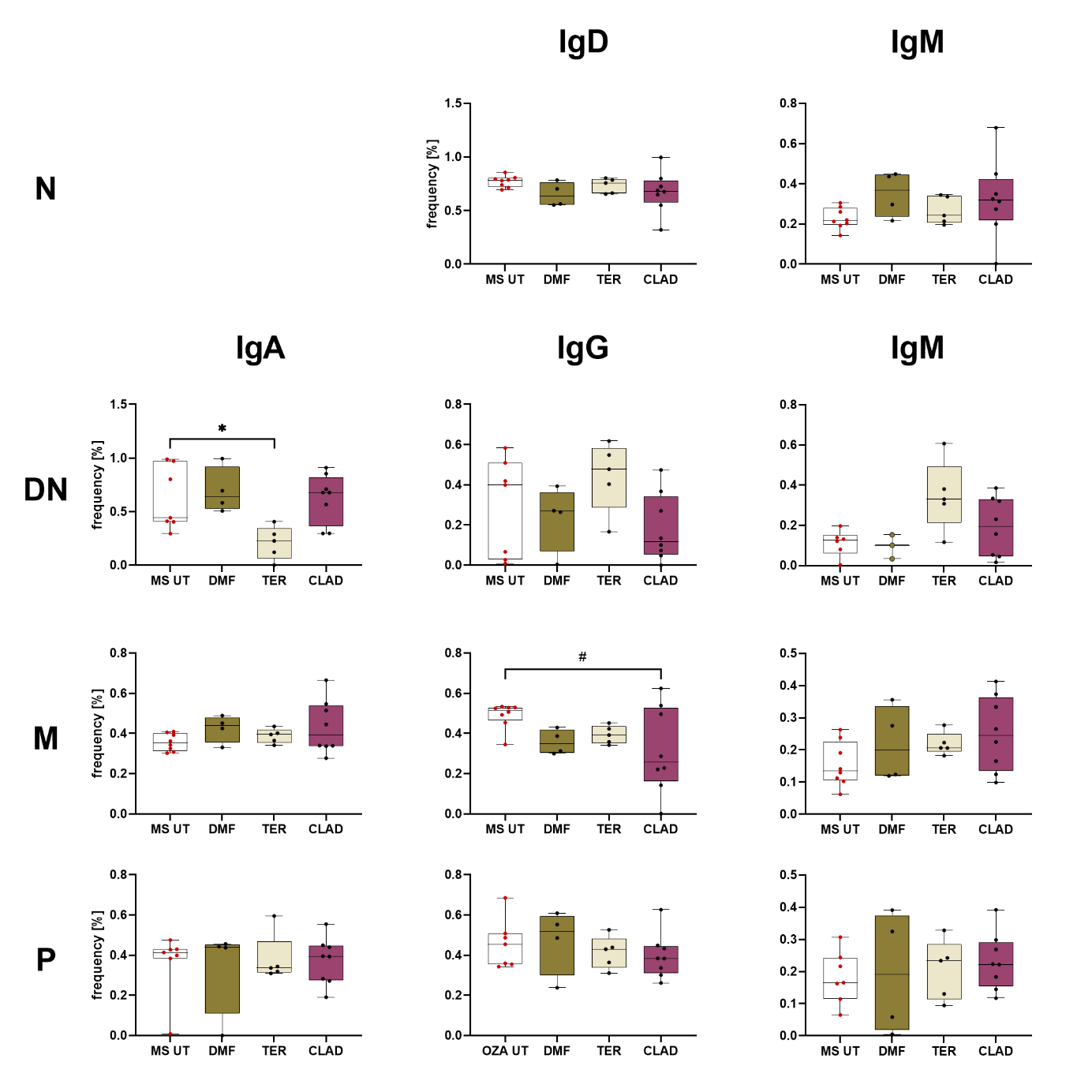
Suppl. Figure 21:** Isotype frequency compared cross-sectionally between DMF, TER and CLAD treated patients (following six months of treatment) and treatment-naïve patients (MS UT) from the OZA cohort (baseline). Comparisons are shown for the isotypes IgD and IgM for the naive B cells and for the isotypes IgA, IgG and IgM for the remaining B cell subsets (DN, M, P). For statistical analyses the Kruskal-Wallis test was performed (^#^p<0.1; *p<0.05).

**
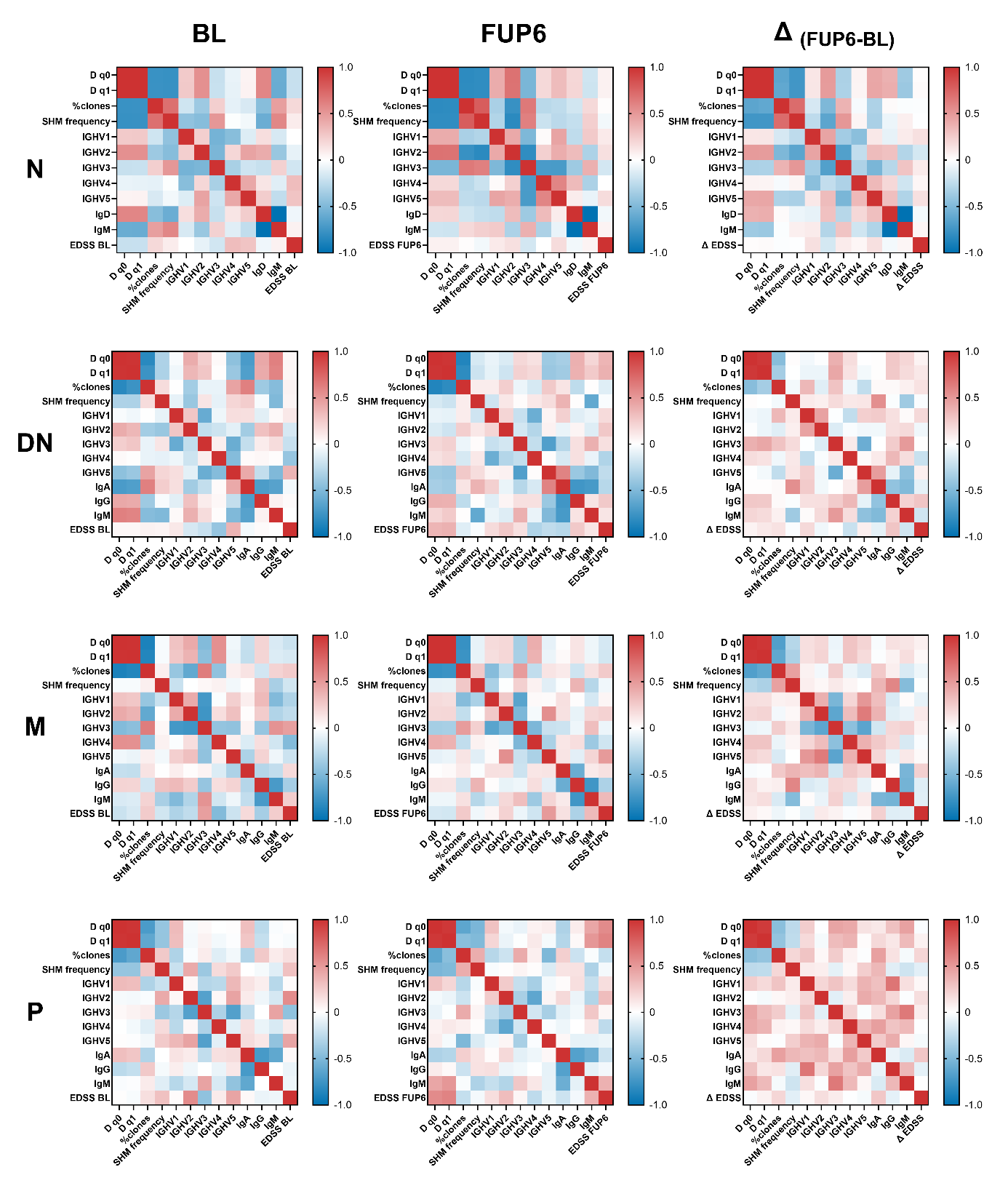
Suppl. Figure 22:** **Correlation analysis between clonal and clinical metrics.** Correlation analyses are shown between all clonal metrics including diversity q0/q1, percentage of clones > 50 sequences, SHM, IGHV family gene usage and isotype frequency, and, EDSS as clinical metric. Correlation analyses were performed for each cell subset (N, DN, M, P) and timepoint separately (BL, FUP6), and, in addition the Δ changes between BL and FUP6 were determined for each metric and also correlated with each other. For statistical analyses the Spearman correlation test was performed. N, naive B cells; DN, double negative B cells; M, memory B cells; P, plasmablasts; BL, baseline; FUP6, follow-up six months; D, diversity; SHM, somatic hypermutation; EDSS: expanded disability status scale.

**
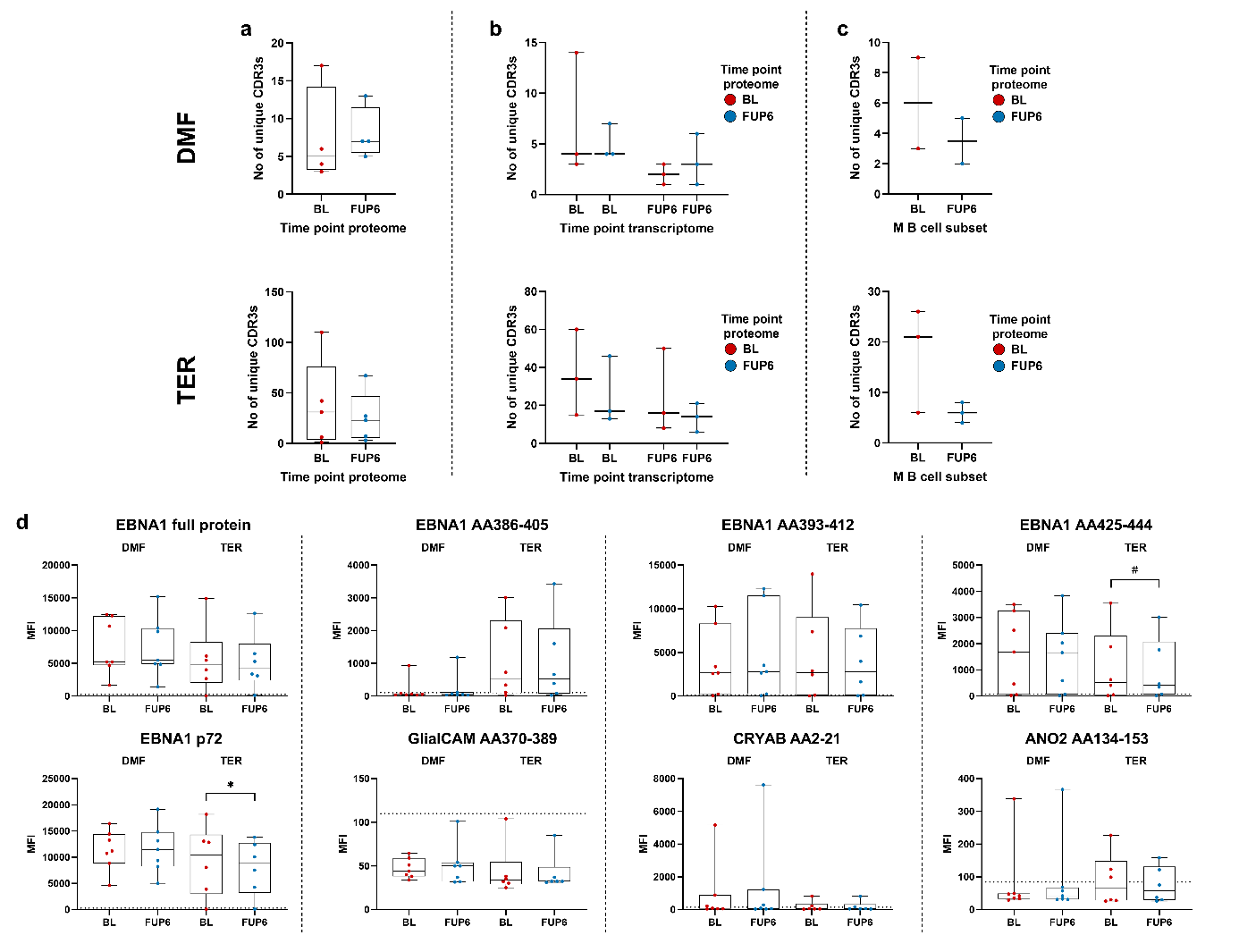
Suppl. Figure 23:** Longitudinal immunoglobulin proteome analysis for the dimethyl fumarate (DMF) and teriflunomide (TER) patient cohorts. Alignment of Ig proteome (Px) libraries to the Ig transcriptome (Tx) libraries with emphasis on **(a)** the total number of recovered unique CDR3 sequences per time point (BL, FUP6), **(b)** matching Ig sequences within one time point and between both time points, and **(c)** matching Ig sequences within the memory B cell population. **(d)** Serum IgG antibody levels at baseline (BL) and after six months of DMF and TER treatment (FUP6) against EBNA1 (full protein, p72), specific EBNA1-peptides (AA386-405, AA393-412, AA425-444) and their respective molecular mimicry peptides GlialCAM (AA370-389), CRYAB (AA2-21) and ANO2 (AA134-153). The horizontal dotted line indicates the cut-off for IgG seropositivity. For statistical analyses the Wilcoxon Rank-Sum paired test and the Friedman-test with Dunn’s correction were performed (#p<0.1; *p<0.05).

**
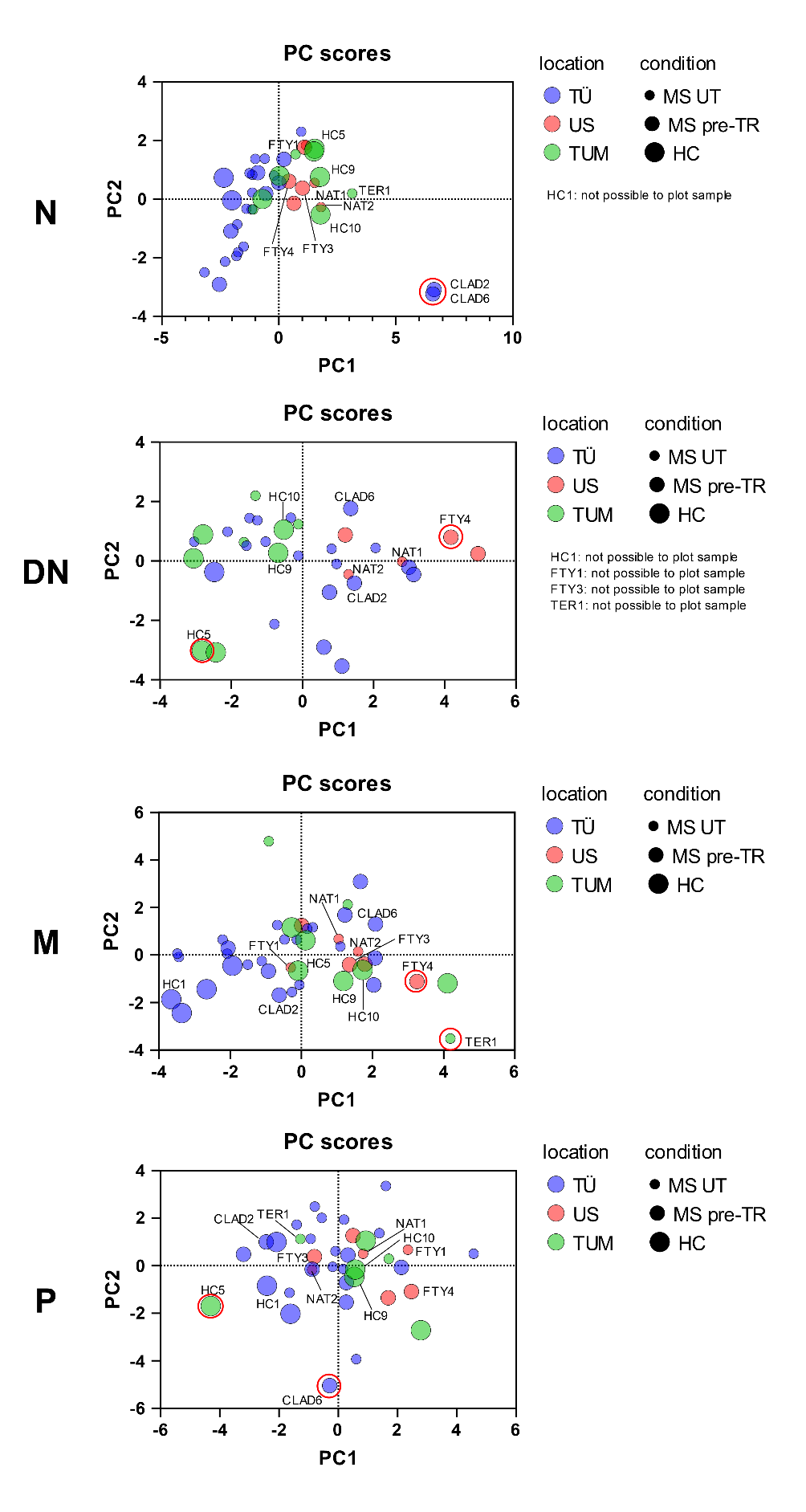
Suppl. Figure 24:** **Principal component analysis.** PCA was conducted for each B cell subset separately with information on diversity q0/q1, percentage of clones > 50 sequences, SHM, IGHV family gene usage and isotype frequency. Visualized samples are separated by corresponding location of data acquisition and condition. From the ROUT method preselected outliers are labelled in each plot and those samples from the preselected outliers that are clearly outlying are circled in red. PC, principal component; N, naive B cells; DN, double negative B cells, M, memory B cells; P, plasmablasts; TÜ, Tübingen; US, United States; TUM, Technical University of Munich; MS UT, MS untreated/treatment-naïve; MS pre-TR, MS pretreated; HC, healthy control.

**
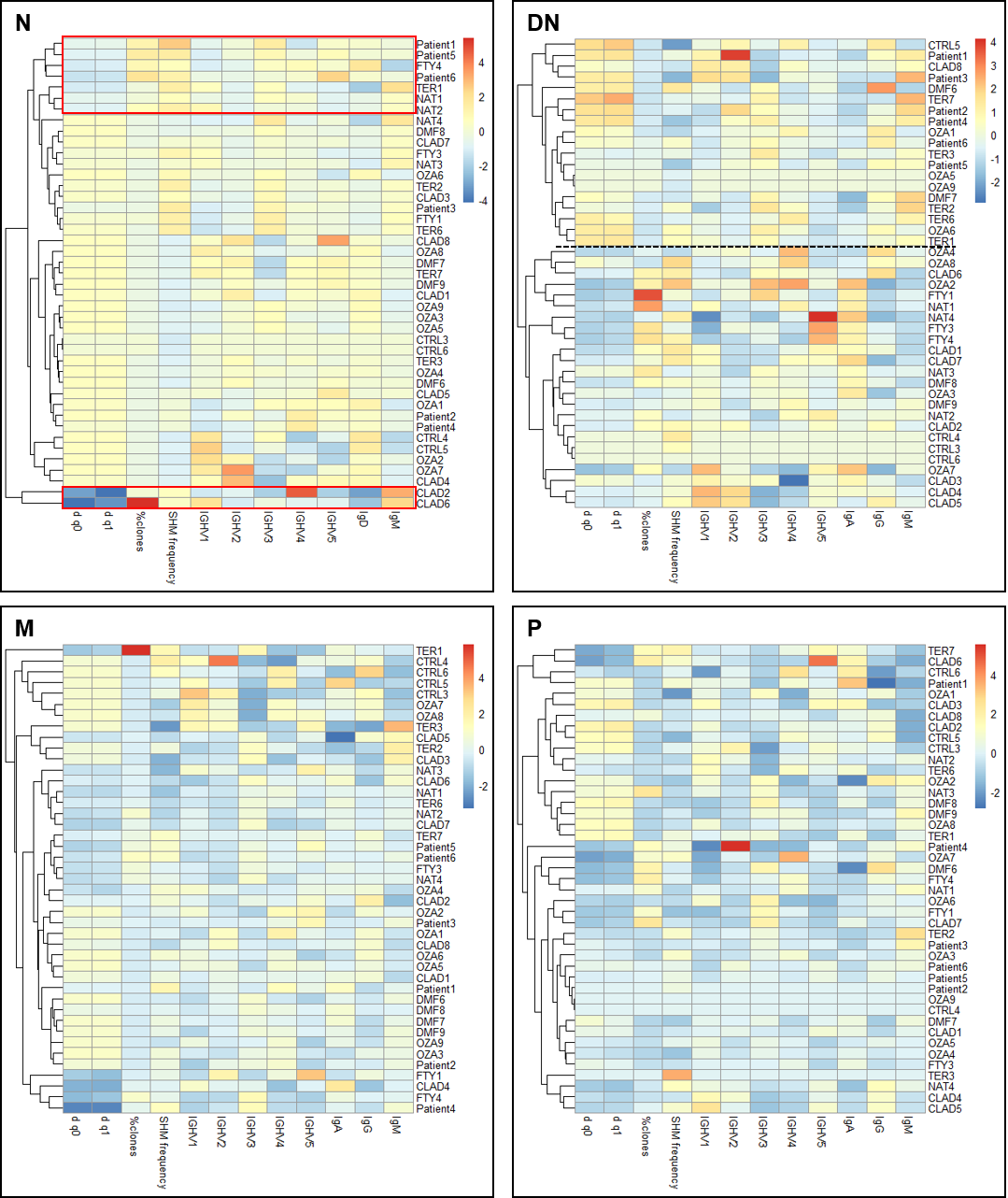
Suppl. Figure 25:** **Hierarchical Clustering.** Clustering was conducted for each B cell subset separately with information on diversity q0/q1, percentage of clones > 50 sequences, SHM, IGHV family gene usage and isotype frequency. Outlying samples are framed in red. N, naive B cells; DN, double negative B-cells, M, memory B-cells; P, plasmablasts; OZA, ozanimod; FTY, fingolimod; NAT, natalizumab; DMF, dimethyl fumarate; TER, teriflunomide; CLAD, cladribine; HC, healthy control; CTRL3 ≙ HC1; CTRL4 ≙ HC2; CTRL5 ≙ HC3; CTRL6 ≙ HC4; Patient1 ≙ HC5; Patient2 ≙ HC6; Patient3 ≙ HC7; Patient4 ≙ HC8; Patient5 ≙ HC9; Patient6 ≙ HC10. For samples with missing values for clonal analysis, due to not passing the filtering threshold of minimum n > 100 sequences, mean values were calculated automatically when applying hierarchical clustering method.

**
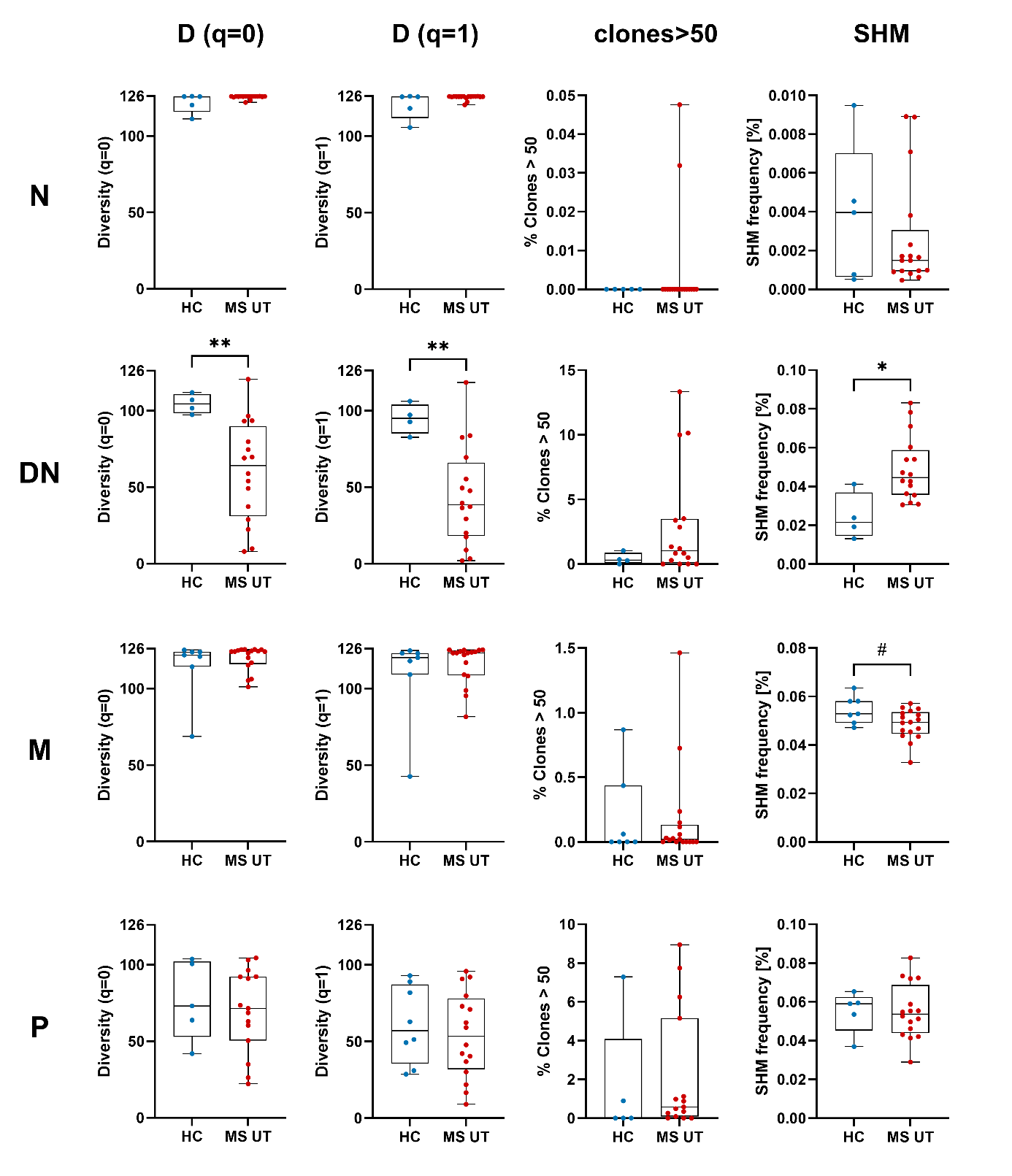
Suppl. Figure 26:** **Clonal analysis for the cross-sectional comparison between healthy controls and treatment-naïve pwMS after outlier removal.** The HC cohort after removing HC5, HC9 and HC10 is compared cross-sectionally to the MS UT cohort after removing TER1 in addition to the FTY and NAT data set. Each column represents one metric including clonal diversity expressed as Hill numbers for q = 0 and q = 1, accounting for the total number of clones and weighted by clone size, respectively, percentage of clones comprising more than 50 unique sequences per clone, and, somatic hypermutation frequency. Comparison data are shown per row for one B cell subset (N, DN, M, P). For statistical analyses the Mann Whitney test was performed (^#^p<0.1; **p<0.01). N, naive B cells; DN, double negative B cells; M, memory B cells; P, plasmablasts; HC, healthy control; MS UT, treatment-naïve pwMS; D, diversity; SHM, somatic hypermutation.

**
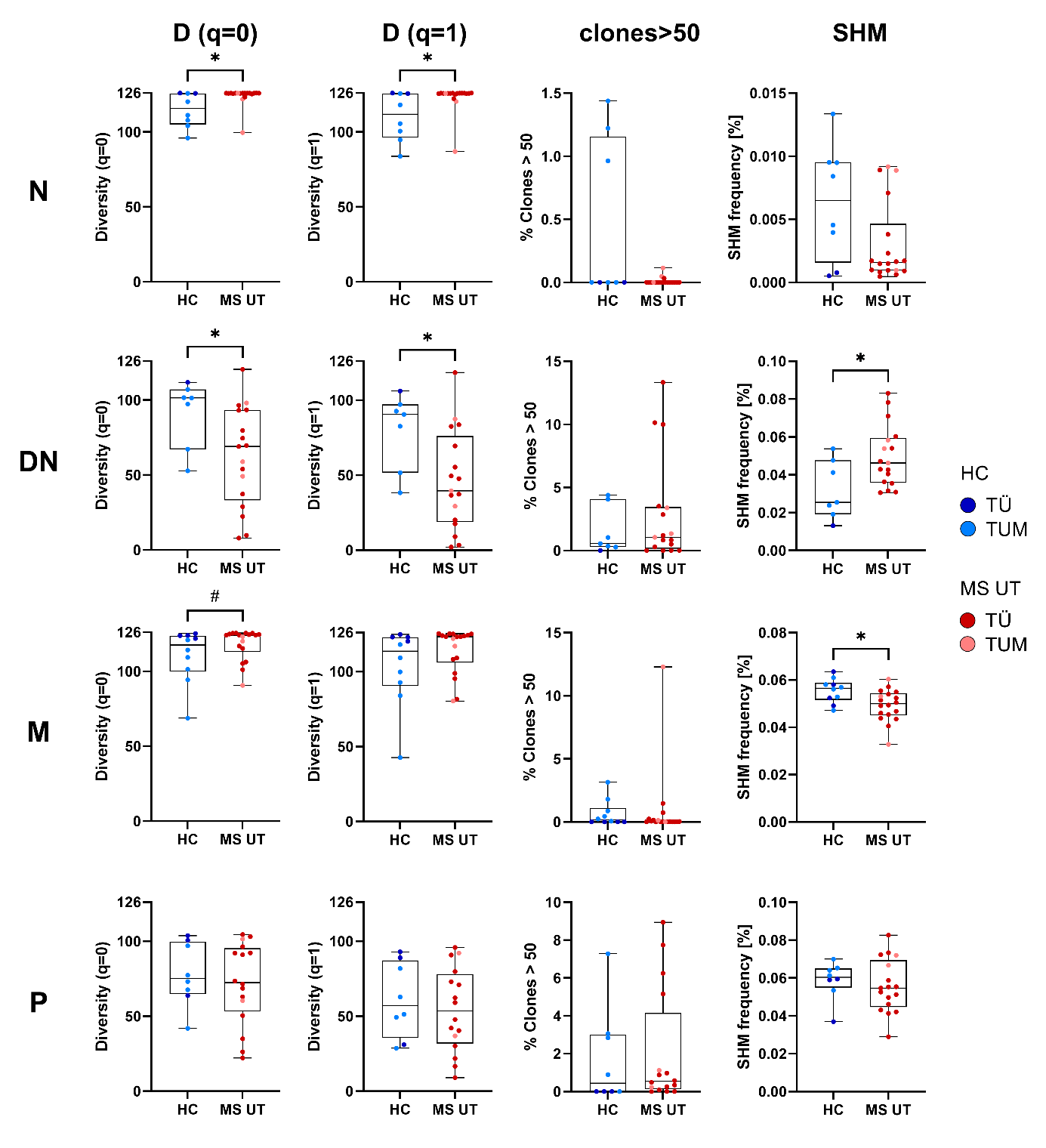
Suppl. Figure 27:** **Clonal analysis for the cross-sectional comparison between healthy controls and treatment-naïve pwMS separated by TÜ and TUM batch.** Each column represents one metric including clonal diversity expressed as Hill numbers for q = 0 and q = 1, accounting for the total number of clones and weighted by clone size, respectively, percentage of clones comprising more than 50 unique sequences per clone, and, somatic hypermutation frequency. Comparison data are shown per row for one B cell subset (N, DN, M, P). The HC and MS UT data sets are separated by batch: TÜ samples are coloured in dark blue and dark red and TUM samples are coloured in light blue and light red. For statistical analyses the Mann Whitney test was performed (^#^p<0.1; **p<0.01). N, naive B cells; DN, double negative B cells; M, memory B cells; P, plasmablasts; HC, healthy control; MS UT, treatment-naïve pwMS; D, diversity; SHM, somatic hypermutation; TÜ, Tübingen; TUM, Technical University of Munich.
